# Development and application of a PBPK modeling strategy to support antimalarial drug development

**DOI:** 10.1002/psp4.13013

**Published:** 2023-08-16

**Authors:** Nada Abla, Eleanor Howgate, Karen Rowland‐Yeo, Maurice Dickins, Mackenzie C. Bergagnini‐Kolev, Kuan‐Fu Chen, Savannah McFeely, Jennifer J. Bonner, Laura G. A. Santos, Nathalie Gobeau, Howard Burt, Zoe Barter, Hannah M. Jones, David Wesche, Susan A. Charman, Jörg J. Möhrle, Jeremy N. Burrows, Lisa M. Almond

**Affiliations:** ^1^ Medicines for Malaria Venture Geneva Switzerland; ^2^ Certara UK Ltd, Simcyp Division Sheffield UK; ^3^ Certara USA, Integrated Drug Development Grand Rapids Michigan USA; ^4^ Monash University Melbourne Victoria Australia

## Abstract

As part of a collaboration between Medicines for Malaria Venture (MMV), Certara UK and Monash University, physiologically‐based pharmacokinetic (PBPK) models were developed for 20 antimalarials, using data obtained from standardized in vitro assays and clinical studies within the literature. The models have been applied within antimalarial drug development at MMV for more than 5 years. During this time, a strategy for their impactful use has evolved. All models are described in the supplementary material and are available to researchers. Case studies are also presented, demonstrating real‐world development and clinical applications, including the assessment of the drug–drug interaction liability between combination partners or with co‐administered drugs. This work emphasizes the benefit of PBPK modeling for antimalarial drug development and decision making, and presents a strategy to integrate it into the research and development process. It also provides a repository of shared information to benefit the global health research community.

**Study Highlights**

**WHAT IS THE CURRENT KNOWLEDGE ON THIS TOPIC?**

Physiologically‐based pharmacokinetic (PBPK) models are routinely used to predict drug–drug interactions (DDIs) and pharmacokinetics in special populations. Artemisinin‐based combination therapies are currently recommended for the treatment of uncomplicated malaria and new fixed‐dose combinations are being developed; hence understanding DDI liability is key.

**WHAT QUESTION DID THIS STUDY ADDRESS?**

How can PBPK modeling support the optimal use of marketed antimalarial drugs and the development of new combinations?

**WHAT DOES THIS STUDY ADD TO OUR KNOWLEDGE?**

PBPK models were developed for the most commonly used antimalarial drugs (and 2 compounds in development) and verified against available clinical data. They have been used for various applications and a PBPK modeling strategy for supporting antimalarial drug development has been implemented.

**HOW MIGHT THIS CHANGE DRUG DISCOVERY, DEVELOPMENT, AND/OR THERAPEUTICS?**

These PBPK models can be used to inform potential dose adjustment of fixed dose combinations when a DDI is anticipated between combination partners or with co‐administered drugs.

## INTRODUCTION

Malaria is a public health imperative. In 2021, the estimated number of malaria deaths worldwide was 619,000, with ~76% being children aged under 5 years. The current World Health Organization (WHO) recommendation for the treatment of uncomplicated malaria caused by *Plasmodium falciparum* is artemisinin‐based combination therapies (ACTs).^1^ Existing ACTs are fixed‐dose combinations (FDCs) and are given as three or six doses over 3 days. The ideal strategy for the future is to give single‐dose FDCs.^2^

When partnering drugs in FDCs, a quantitative understanding of the pharmacokinetic (PK) behavior and assessment of drug–drug interaction (DDI) liability accounting for victim and perpetrator properties of each drug is required for dose optimization. Such DDIs may occur between combination partners, but also with concomitant medications in patients suffering from other diseases. Any DDIs should be risk assessed in terms of safety and therapeutic failure. Moreover, a quantitative understanding of how drug exposure may be affected by intrinsic patient factors, including malaria infection, age, or pregnancy, is required to avoid suboptimal dosing that can be experienced in these populations,^3^ or during off‐label dosing when limited information is available.^4^

Physiologically‐based pharmacokinetic (PBPK) modeling combines drug parameters (such as physicochemical properties and in vitro determinations of intrinsic clearance) with system parameters (such as demographics and physiology) to predict PKs across populations of virtual subjects.^5^ With this approach, the impact of intrinsic and extrinsic factors on drug disposition can be assessed. The use of PBPK modeling to predict DDI liability, informing wording on drug labels, and replacing the need for some clinical DDI studies, is now commonplace within drug development.^6^, ^7^, ^8^, ^9^, ^10^ Furthermore, PBPK has been increasingly used to guide clinical practice and drug combinations in some disease areas, such as HIV, cystic fibrosis, tuberculosis (TB), and coronavirus disease 2019 (COVID‐19).^11^, ^12^, ^13^, ^14^, ^15^, ^16^, ^17^, ^18^ In addition, PBPK modeling enables the prediction of drug PKs in different populations and has the potential to guide dose adjustment.^19^, ^20^, ^21^ Therefore, the development of PBPK models for antimalarial drugs will enable efficient evaluation of DDI liabilities and dose extrapolation in specific populations.

Despite decades of clinical use to treat malaria, many legacy antimalarial drugs do not have comprehensive state‐of‐the‐art data packages that are typically available for drugs developed and approved using modern‐day drug discovery and development processes. Such data packages include in vitro data alongside clinical data, such as single and multiple ascending dose studies, DDI studies, food effect, and more recently mass balance studies, all of which can be readily used to develop PBPK models. The development of models for legacy antimalarials thus relied on both in vitro and clinical data within the literature, which were incomplete in many instances. For this reason, in vitro drug metabolism and pharmacokinetic (DMPK) data were generated using state‐of‐the‐art assays to fill the gaps and give protocol harmonization among the data packages for the different drugs.^22^

In this paper, we summarize a collaborative effort among Medicines for Malaria Venture (MMV), Certara, and Monash University to develop and verify PBPK models for drugs to treat neglected tropical diseases, of which malaria was the main focus. The developed models and associated supplementary information have been made available freely to software users in a PBPK model repository (Simcyp Global Health repository).

Besides presenting the construction of drug models, we share our experiences, including application of the models in the development and clinical uses of antimalarials, where many older legacy drugs remain the cornerstone of therapy and effective combination therapy is the goal. We also present a strategy for including PBPK modeling in the research and development process and for providing continuous and cross‐functional support to the antimalarial drug portfolio.

## METHODS

The major “legacy” antimalarial compounds were included in this work: artemether, dihydroartemisinin (administered as such or formed after administration of artesunate or artemether), lumefantrine, piperaquine, pyronaridine, mefloquine, amodiaquine (including its active metabolite: desethylamodiaquine), sulfadoxine, pyrimethamine, atovaquone, proguanil (including its metabolite responsible for antimalarial activity cycloguanil), chloroquine, azithromycin, doxycycline, quinine, primaquine (including its metabolite carboxyprimaquine), and tafenoquine, as well as two new antimalarial compounds MMV048 and DSM265 (including its metabolite: DSM450), both of which reached phase II clinical development.^23^

### Approach for compound model development and verification

**FIGURE 1:**
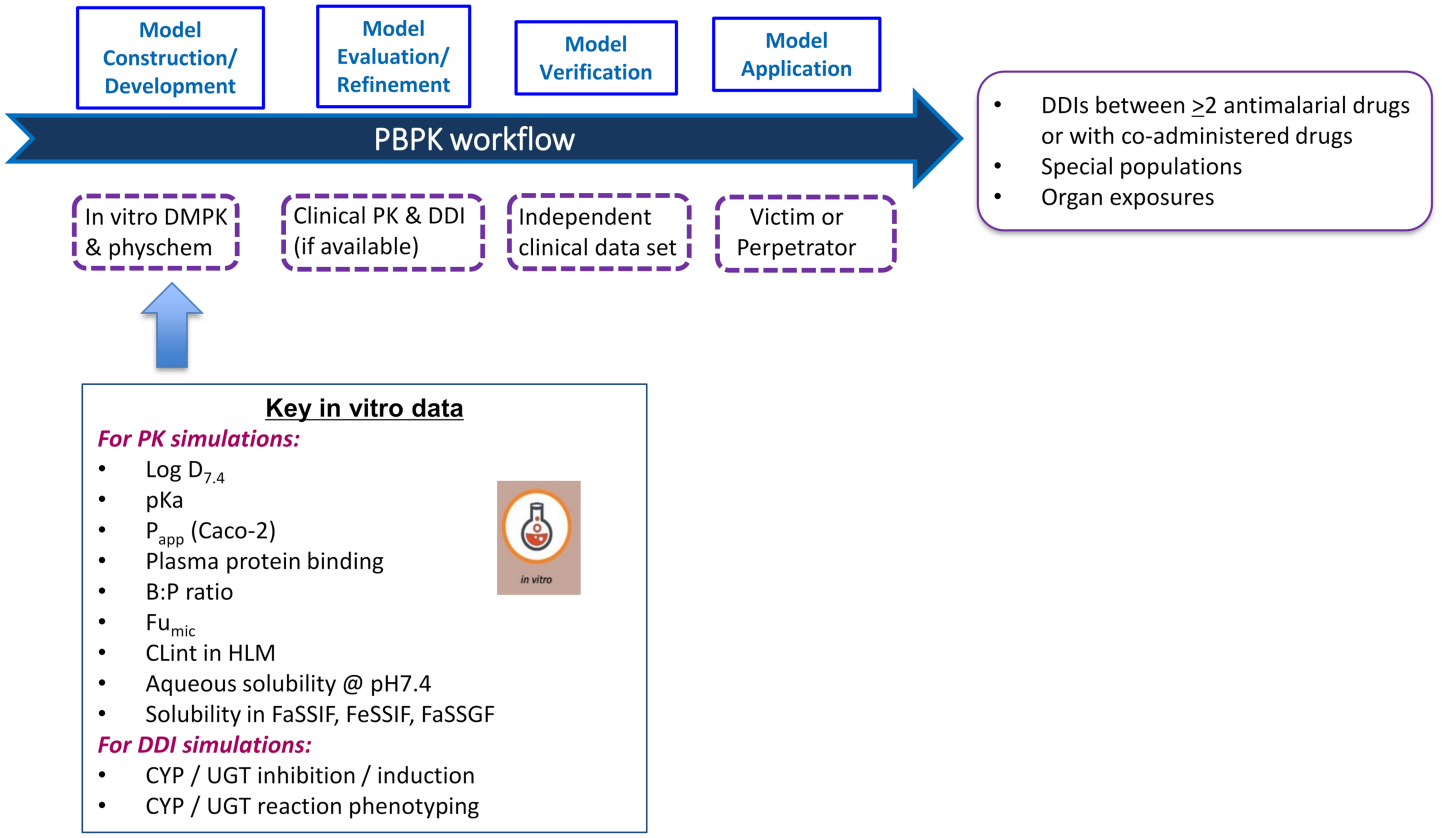
PBPK workflow for DDI risk assessment of antimalarial drugs and other applications. B:P, blood‐to‐plasma ratio; DDI, drug‐drug interaction; DMPK, drug metabolism and pharmacokinetic; FaSSGF, fasted state simulated gastric acid fluid; FaSSIF, fasted state simulated intestinal fluid; FeSSIF, fed state simulated intestinal fluid; Fu_mic_, fraction of drug that is free from non‐specific binding in the HLM assay; HLM, human liver microsomes; P_app_, apparent permeability; PBPK, physiologically‐based pharmacokinetic; PK, pharmacokinetic.

The PBPK models for each compound were built following an approach generally adopted by the pharmaceutical industry.^24^, ^25^, ^26^ Figure 1 summarizes this approach. Additional information on model development and validation, simulation design, and the virtual populations selected can be found in Appendix S1. The model development and verification strategies are outlined in the Supplemental Information (Appendix S2), input parameters for all compounds are listed in Table S1 and model verification is provided in Figures [Link], [Link], and Tables S2 and S3.

### Model applications

Once the PBPK models were adequately verified and deemed “fit‐for‐purpose,” they were applied to answer various questions relating to the development and management of antimalarial drug therapy. Here, we provide three example applications where the available PBPK models were used to address questions relating to DDI assessment. The predicted DDI was considered clinically significant if the ratio of exposures (area under the curve [AUC] and/or maximum plasma concentration [*C*_max_]) in the presence versus in the absence of the perpetrator was less than or equal to 0.8 (for inducers) or greater than or equal to 1.25 (for inhibitors).^27^, ^28^ Additional simulation methods are provided in Appendix S1.

#### Application 1: Investigation of the victim and perpetrator potential of pyronaridine

Pyronaridine is an antimalarial drug used in combination with artesunate as an FDC (pyronaridine‐artesunate, Pyramax) for the treatment of *Plasmodium falciparum* uncomplicated malaria and for the blood stage of *Plasmodium vivax* malaria. Here, we share how the PBPK model for pyronaridine was applied to assess two types of DDI risks raised during the regulatory submission process. The first one was pyronaridine as a P‐gp inhibitor where we evaluated the impact of co‐administration of pyronaridine on the exposure of the P‐gp substrates dabigatran etexilate and digoxin. The second one was the assessment of the liability of pyronaridine as a victim of CYP3A4‐mediated induction, using rifampicin as a typical inducer. Simulations were performed using the dose of pyronaridine given to patients weighing 65 kg or more (720 mg tetraphosphate, equivalent to 410 mg free base).

#### Application 2: Investigation of the impact of rifabutin on the widely used artemisinin‐based combination therapy artemether‐lumefantrine

In addition to having the highest burden of malaria cases, Africa also accounts for the highest (25%) number of TB cases globally.^29^ Rifampicin is one of the main drugs used to treat TB. However, its potent enzyme inducing properties give rise to DDIs with drugs metabolized by CYP enzymes potentially reducing the efficacy of therapy. The combination of artemether and lumefantrine is used to treat malaria in adults and children. Both drugs are metabolized by CYP3A4 and co‐administration with strong inducers, such as rifampicin, is contraindicated on the label. Rifabutin is a rifamycin, which, like rifampicin, works via inhibition of DNA‐dependent RNA synthesis in prokaryotes. Rifabutin is a moderate CYP3A4 inducer and can be a useful option when DDIs with rifampicin are a likely concern, such as in HIV or malaria‐infected patients that are co‐infected with TB. There is a call for global access to rifabutin for this reason^30^ and for cases where there is resistance to rifampicin.^31^ Rifabutin could potentially be a useful treatment option in these cases, however, the CYP3A4‐mediated interaction between rifabutin and artemether‐lumefantrine has not been investigated clinically. Simulations were, therefore, used to assess the likely magnitude of DDI and the suitability of this combination in patients co‐infected with malaria and TB. The effect of chronic rifabutin dosing (300 mg once a day for 28 days) on artemether‐lumefantrine treatment (3 days of treatment with twice a day administration of a FDC of artemether [80 mg] and lumefantrine [480 mg] starting 10 days after rifabutin) was simulated.

#### Application 3: Guidance on dose adjustment when two developmental compounds are co‐administered (potential UGT‐mediated DDI)

A combination therapy dosing strategy was assessed real‐time for two compounds, DSM265 and MMV048, during the drug development process. MMV048 is a UGT1A1 substrate (fraction metabolized [*f*_m_] = 0.9) whereas the metabolite of DSM265, DSM450, inhibits UGT1A1 in vitro with a *K*_*i*_ ~ 1 μM. The aim of this simulation was to determine whether a dose adjustment would be needed for MMV048, when given in combination with DSM265. The dosing regimen used for this simulation was a single dose of 120 mg of MMV048 and 400 mg of DSM265 given concomitantly, the regimen intended to be used in patients.

## RESULTS

### Model development and verification for DDI predictions

Twenty‐four model files were developed and verified (including relevant metabolites, when applicable).

Key victim and perpetrator properties for the prioritized legacy compounds are shown in Table 1. These data are mainly based on in vitro determinations. The table highlights cases of overlapping enzymes in victim and perpetrator properties and hence gives a useful qualitative flag. PBPK modeling is then used to relate the dynamic unbound concentrations of the perpetrator to its measured inhibition/induction properties (e.g., *K*_*i*_ in the case of inhibition) in order to quantify DDI liability.

**TABLE 1:** In vitro flags for victim and perpetrator properties conferring DDI liability.

Available PBPK model	Risk as a victim	Risk as a perpetrator
Amodiaquine	Substrate of CYP2C8	CYP2D6 inhibitor
Artemether	Substrate of CYP2B6 and CYP3A4	CYP2B6 inducer
Atovaquone	Cleared by biliary excretion	BCRP inhibitor
Azithromycin	Cleared by biliary excretion	CYP3A4 time‐dependent inhibitor
Chloroquine	Substrate of CYP2C8 and CYP3A4; renally cleared	CYP2D6 inhibitor
Dihydroartemisinin (DHA)	Substrate of UGT1A9 and UGT2B7	CYP1A2 inhibitor
Doxycycline	Cleared by renal and biliary excretion	None identified
Lumefantrine	Substrate of CYP3A4	CYP2D6 inhibitor
Mefloquine	Substrate of CYP3A4	CYP2D6 inhibitor
Piperaquine	Substrate of CYP3A4, CYP2C9 and CYP2C19	CYP3A4 time‐dependent inhibitor
Primaquine	Substrate of MAO and CYP2D6	CYP1A2 inhibitor
Proguanil	Substrate of CYP2C19 and CYP3A4	CYP2D6 inhibitor
Pyrimethamine	Incomplete information	OCT2 and MATE inhibitor
Pyronaridine	Substrate of CYP1A2, CYP2B6, CYP2C8, CYP2D6 and CYP3A4	CYP2D6 and P‐gp inhibitor
Quinine	Substrate of CYP3A4	CYP2D6 inhibitor
Sulfadoxine	Renally cleared	None identified
Tafenoquine	Incomplete information	CYP3A4 and CYP2C9 inhibitor

Abbreviations: DDI, drug‐drug interaction; PBPK, physiologically‐based pharmacokinetic.

### Case studies – Prediction of antimalarial DDI liabilities

In the first application, the pyronaridine PBPK model was first verified against clinically observed findings and was able to recover observed plasma concentration‐time profiles and exposure levels after single oral doses of 307 mg (free base) and 3.42–8.54 mg/kg in healthy Thai and Korean subjects that were within 1.5‐ and 2.0‐fold of observed data, respectively^32^, ^33^ (Figure S1). The PBPK model was also able to generate PK parameters for pyronaridine after three multiple doses of 410 mg free base q.d. that were within 1.6‐fold of the observed data in healthy White subjects.^34^ The verified model was then used prospectively to predict the likely outcome of DDI with the P‐gp substrates, dabigatran etexilate and digoxin, and with the CYP3A4 inducer, rifampicin.

**FIGURE 2:**
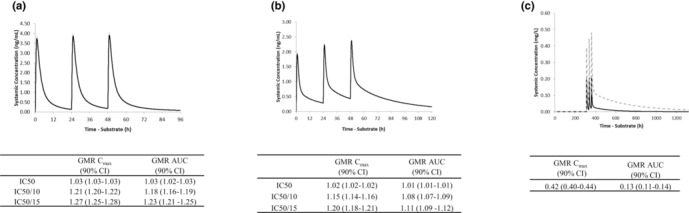
Simulated mean (a) dabigatran etexilate and (b) digoxin plasma concentration‐time profiles in the absence of (black line) and in combination with (dashed line) pyronaridine dosing (410 mg pyronaridine free base q.d. for 3 days) in healthy subjects. Note the dashed line is not visible as it is superimposed to the black line in both cases. The geometric mean ratios (GMRs) for dabigatran and digoxin in the presence and absence of pyronaridine are also shown below the corresponding figures. Simulated mean pyronaridine plasma concentration‐time profiles (c) and geometric mean *C*_max_ and AUC ratios following repeat dosing (410 mg free base q.d. for 3 days in the absence [gray dashed line] and in combination with [black line] rifampicin dosing [600 mg q.d. for 55 days]) in healthy subjects. AUC, area under the curve; CI, confidence interval; *C*_max_, maximum plasma concentration; IC_50_, half‐maximal inhibitory concentration.

Using the measured in vitro P‐gp inhibition data as input to the model, the predicted DDI with dabigatran etexilate and digoxin was minimal (dabigatran etexilate *C*_max_ ratio 1.03 and AUC ratio 1.03; digoxin *C*_max_ ratio 1.02 and AUC ratio 1.01). An additional sensitivity analysis was performed to evaluate the impact of *K*_*i*_ on the estimated DDI magnitude. DDI simulations were repeated after reducing the *K*_*i*_ values by up to 15‐fold. The objective of this sensitivity analysis was to account for the “worst‐case” scenario of potentially inaccurate pyronaridine *K*_*i*_ estimates from in vitro transporter inhibition experiments as is routinely done.^35^, ^36^ The results of these analyses are shown in Figure 2a,b. Prospective use of the pyronaridine model to predict the likely interaction with rifampicin (600 mg q.d.) following three oral doses of 410 mg free base q.d. of pyronaridine (corresponding to 720 mg of the tetraphosphate salt) predicted that there would be a decrease in pyronaridine exposure with geometric mean ratios of 0.42 for *C*_max_ and 0.13 for AUC (Figure 2c). This analysis indicates the likelihood of an interaction with the P‐gp substrates dabigatran etexilate and digoxin is low even after applying sensitivity analysis (<1.28‐fold for dabigatran etexilate and <1.20‐fold for digoxin). Simulations with the strong CYP3A4 inducer (rifampicin) predicted a strong interaction (AUC decreased by 87%).

**FIGURE 3:**
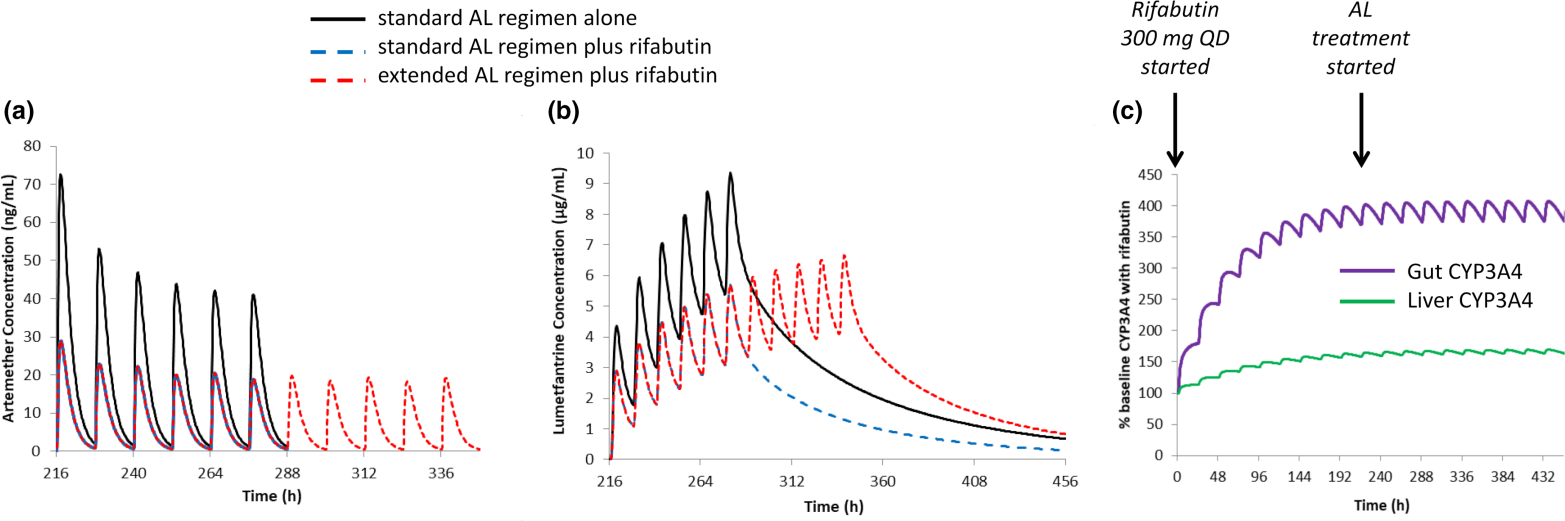
Simulations of (a) artemether (standard regimen 80 mg b.i.d. for 6 doses; extended regimen 80 mg b.i.d. for 11 doses), (b) lumefantrine (standard regimen 480 mg b.i.d. for 6 doses; extended regimen 480 mg b.i.d. for 11 doses), and (c) simulated CYP3A4 expression in patients receiving rifabutin for 28 days (300 mg q.d. starting 10 days before malaria treatment). AL, artemether‐lumefantrine.

The second application shows a prospective DDI simulation between a marketed antimalarial combination, artemether‐lumefantrine (AL), and the rifamycin antibiotic, rifabutin, a moderate CYP3A4 inducer, which can be used to treat TB. A comparison of simulated and observed PK profiles for artemether and lumefantrine under different dosing regimens are shown in Figures S2 and S3, respectively. The simulated AUC and *C*_max_ ratios in the presence and the absence of a typical therapeutic dose of rifabutin (300 mg q.d.) were 0.49 and 0.36 for artemether, and 0.55 and 0.61 for lumefantrine, respectively (Figure 3), suggesting a significant impact of the co‐administration of rifabutin on the PKs of AL, which may lead to suboptimal exposure. This is primarily a concern for loss of efficacy but also potentially for the risk of selecting strains resistant to this antimalarial combination. The models were applied to propose possible dose adjustments to maintain AL exposures once rifabutin treatment is initiated. In the case of enzyme induction, there are potential safety concerns with increasing absolute doses, especially when *C*_max_ driven toxicity, such as QTc prolongation, has been reported, like for lumefantrine.^37^ It is also known that the intestinal absorption of lumefantrine becomes saturated at higher doses.^38^ For these reasons, an alternative approach increasing the length of the dosing course to ensure equivalent exposure was explored rather than simply increasing the dose. Simulations showed that an increase from the standard 3‐day regimen (6 doses) to a 5.5‐day regimen (11 doses) when co‐administered with rifabutin (300 mg q.d.) resulted in total AUC of AL similar to that observed when AL was dosed alone. This is in line with the dose adjustment that has been suggested for AL in pregnant women (where the exposure to both drugs is reduced^38^) and in HIV‐co‐infected patients treated with efavirenz, where a 5‐day regimen has been suggested.^39^, ^40^

**FIGURE 4:**
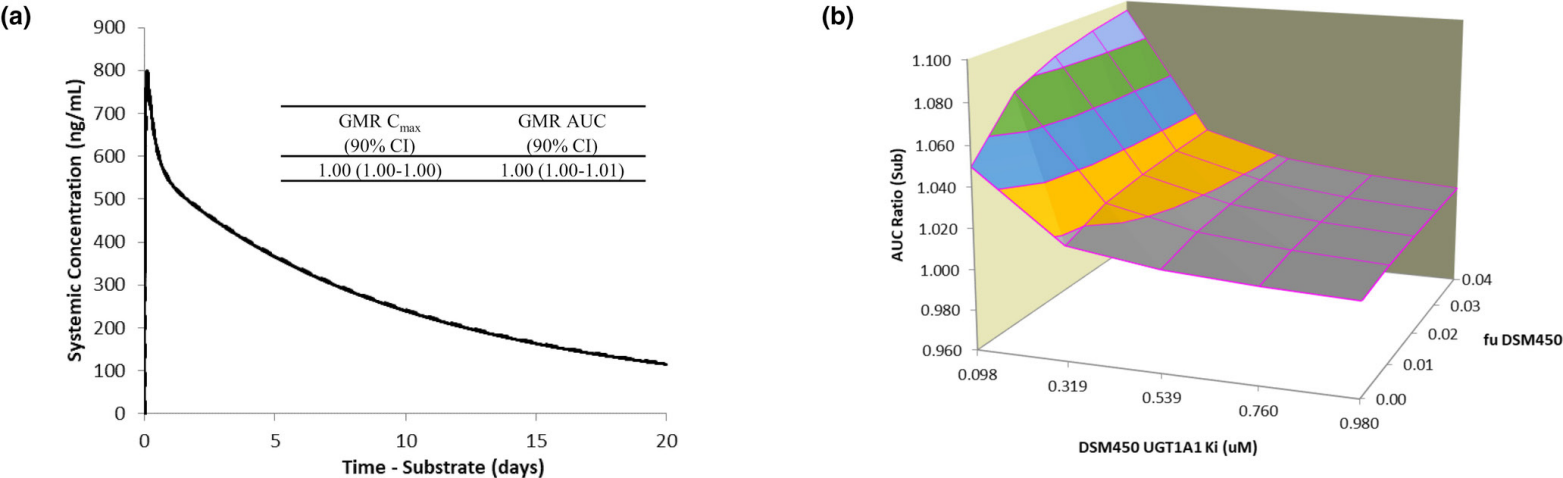
(a) Simulated mean MMV048 (120 mg single dose) plasma concentration‐time profiles in the absence (black line) and in combination with (dashed line) DSM265 dosing (400 mg single dose) in healthy volunteers for 20 days and (b) sensitivity analysis assessing the interaction potential when the *f*_u_ for the inhibitory metabolite DSM450 is 10‐fold higher and the *K*_*i*_ is 10‐fold lower. AUC, area under the curve; CI, confidence interval; *C*_max_, maximum plasma concentration; *f*_u_, unbound fraction; GMR, geometric mean ratio; *K*_i_, inhibition constant.

The third application was a prospective simulation of the human PK profiles of two antimalarial compounds under development, DSM265 and MMV048, to inform on whether a dose adjustment should be made when combining these two new compounds in an FDC. MMV048 is mainly metabolized by UGT1A1 (*f*_m_ 0.9), and DSM450, the primary metabolite of DSM265, was shown in vitro to inhibit UGT1A1 with a *K*_*i*_ of ~1 μM. Simulation of the PK of MMV048 after a single dose of 120 mg given with or without 400 mg of DSM265 suggests that the DDI will not be significant in vivo (Figure 4a), with AUC and *C*_max_ ratios of ~1. A sensitivity analysis was performed on the DSM450 parameters with higher uncertainty (unbound fraction [*f*_u_] and inhibition constant [*K*_*i*_]) and showed that even with a 10‐fold decrease in *K*_*i*_ and 10‐fold increase in the *f*_u_, the AUC ratio is still only 1.1, and hence not clinically relevant (Figure 4b).

## DISCUSSION

This work was a collaborative effort to develop and publish PBPK models relevant to the development and life‐cycle management of antimalarial drugs. Twenty‐four drug models were developed and validated for the 18 most widely used antimalarials (and 3 associated metabolites) along with two antimalarial drugs in development (and 1 metabolite). The models have been made available in a global health PBPK model repository (https://members.certara.co.uk/Simcyp/GlobalHealthRepository), together with slide decks summarizing the input parameters and the clinical studies used to validate the models, as well as the robustness and the scope of use. Models can be accessed freely following a registration step.

To facilitate the investigation of real‐life scenarios on comorbidities, the repository also contains PBPK models for TB drugs,^14^ anti‐HIV drugs, and virtual disease populations describing altered physiology. The aim of the repository is that models should be considered “live” and “off‐the‐shelf,” hence it is good practice to assess and expand the models when new data become available, and report improved versions so that the community of antimalarial research can benefit from the latest knowledge.

Through the development and application of these models we have sought to establish a practical strategy for the use of legacy drug models within development programs, particularly in diseases like malaria where old drugs remain the cornerstone of combination drug therapy. The models presented in this work were developed after market approval (for legacy compounds) or during development (for DSM265 and MMV048). The models were developed in 2017 (Simcyp Version 16), re‐assessed/updated in Simcyp Version 19, and have been widely used at MMV since then. In the first instance, the models were used to perform DDI simulations and have now evolved to include wider applications. This work has had significant impact on our antimalarial drug development programs, for example, by facilitating the choice of the combination partner, allowing de‐risking of an interaction, or informing whether and how to conduct a clinical DDI study. This is well‐illustrated by the case studies: the simulations with pyronaridine allowed de‐risking the administration with P‐gp substrates and confirmed the risk of reduced efficacy with CYP3A4 inducers. A potential next step would be to re‐visit the prescribing information which currently lists P‐gp substrates as potential victim drugs of pyronaridine; the work on artemether and lumefantrine illustrates how the model can be used to support clinical management by simulating untested clinical scenarios (using confidence built from available clinical DDI data in the case of legacy drugs) and investigating potential dose adjustments; finally, the DDI assessment for DSM265 and MMV048 gave confidence to project teams that the two drugs could be administered without dose adjustments. Although the development of both compounds was halted for other reasons, and the potential DDI could not be tested clinically, these simulations demonstrate how PBPK can be used to assess novel antimalarial combinations and support the design of clinical studies, even before the extent of the DDI has been verified clinically.

The virtual healthy volunteer population was used in all three case studies, assuming the same magnitude of interaction would be observed in malaria‐infected individuals. Work is ongoing to verify DDI predictions in a virtual malaria population to account for changes in blood binding and CYP3A4 expression to incorporate this into our routine DDI strategy going forward.

**FIGURE 5:**
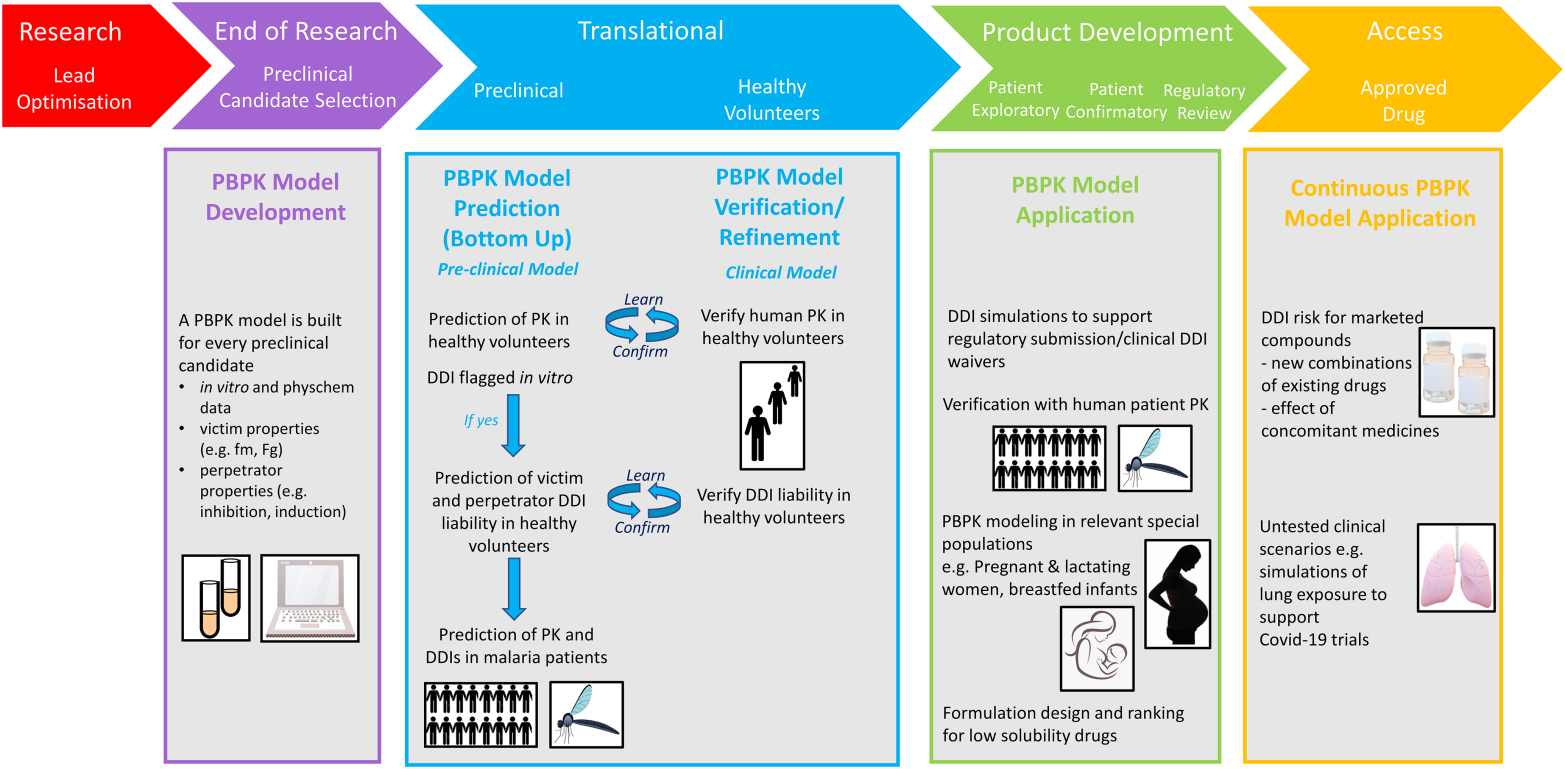
A schematic representation of the cross‐functional PBPK support to antimalarial drug development highlighting the key questions that need to be addressed across Translation, Product Development, and Access phases. The PBPK DDI strategy for antimalarial drug development has been designed based on regulator guidance (Clinical Drug Interaction Studies — Cytochrome P450 Enzyme‐ and Transporter‐Mediated Drug Interactions Guidance for Industry, 2020^27^) and refined based on in house experience over the last 5 years. The PBPK modeling process starts in Preclinical candidate selection where a model is built for every preclinical candidate based on all available physicochemical and in vitro DMPK data. Where a DDI is flagged qualitatively from in vitro data, the PBPK model is used to quantify the DDI risk both as a victim and perpetrator. Once clinical data are available the initial model is assessed, refined (if required) and verified before it is further applied across Product Development and Access stages. Although models are applied at all stages for different purposes, the schematic uses the term “application” for those toward the end of the Drug Development Process that use the final model. COVID‐19, coronavirus disease 2019; DDI, drug‐drug interaction; DMPK, drug metabolism and pharmacokinetic; *f*_m_, fraction of systemic clearance by a particular drug metabolizing enzyme; *f*_g_, fraction escaping gut metabolism on first pass; PBPK, physiologically‐based pharmacokinetic; PK, pharmacokinetic.

Building on our experience, we are presenting here a customized PBPK strategy for antimalarial drug development that is summarized in Figure 5. This strategy is based on the standard PBPK workflow that is followed for drug development in general^24^ but includes some features that are unique to malaria, such as the early search for combination partners in FDCs, the specific target populations (e.g., young children and pregnant women) that require an early understanding of the clearance pathways, and the fact that we still rely heavily on legacy drugs and the older literature studies in which they are described. We believe similar strategies could be adopted and customized for other neglected tropical diseases and areas of global health. For malaria, DDI simulations support each phase of antimalarial drug development, from preclinical to phase III, and after registration. PBPK modeling hence plays a cross‐functional role and allows routine DDI assessment. Building models early on allows the integration of information and the early assessment of potential DDIs, within an antimalarial drug combination or with co‐administered drugs, to treat comorbidities such as TB and HIV. This supports early ranking of combination partners and decision making. Furthermore, being aware of potential interactions enables precautions to be taken to avoid unexpected toxicity or loss of efficacy due to increased or decreased exposure, respectively. It is also considered as a potential opportunity for FDCs, when one compound in the combination increases the exposure of the partner drug, potentially allowing a dose reduction of the latter. For this reason, MMV have now included as a prerequisite for reaching phase 0 and, therefore, as part of the Discovery candidate selection criteria, the generation of all the key in vitro DMPK and physicochemical assays listed in Figure 1 as well as a Simcyp preclinical compound file based on these data. Importantly, these assays have been standardized and preferred providers of DMPK services have been identified for discovery projects led by MMV.

One challenge associated with PBPK modeling is the requirement of robust in vitro DMPK data to fully characterize the physicochemical properties, binding properties and metabolism of each compound. It is key to understand the existing data, limitations of the existing models, and be aware of knowledge gaps. Even though the legacy drugs have been used clinically for years, in most cases, key data such as the contribution of enzymes to the elimination of a drug (*f*_m_) was missing from the literature or values for parameters such as *f*_u_ in plasma or in vitro assays were unavailable or when available, frequently highly variable. In this work, in vitro methodology was standardized, new data generated across the compound dataset,^22^ and independent published clinical studies used to verify the models (Tables S2 and S3) to ensure the PBPK models were developed with robust and transparent methodology. Despite these efforts there remain some drugs for which there is still a poor understanding of clearance pathways. This limits the application of the model, for example, prohibiting use for victim DDI or pediatric assessments. However, if a model can capture the observed clinical plasma concentration‐time profile, it may be used as a DDI perpetrator file. In addition, sharing of the model can facilitate its valid use and future expansion. An example of this is pyrimethamine, where the clearance pathways have not been characterized well enough to enable a good understanding of its victim DDI risk. However, the file has been used as an inhibitor of MATE and OCT2 transporters to help verify the PBPK model for metformin as a substrate of MATE and OCT2 and assess the OCT2 mediated victim liability of a drug in development without the need to generate new data (unpublished work). In this work, sensitivity analysis has also been key to assess the impact of uncertain parameters.

For antimalarial compounds under development, the challenge is slightly different. In these cases, in vitro parameters are measured as standard within drug discovery and development, but the target drug space that is targeting an efficacious once daily antimalarial medicines^2^ also gives us specific challenges for in vitro determinations. For example, obtaining in vitro information on the intrinsic clearance or metabolic pathways (e.g., *f*_m_) for compounds with long half‐lives is a technical challenge despite the evolution of the latest generation of low clearance assays (3D cultures or stabilization of the hepatocytes to prolong their viability in the assay). Characterizing the metabolic pathways is key for predicting the PKs in malaria target populations, such as children and pregnant women, where enzyme maturation and expression can differ from healthy adult volunteers. Therefore, additional effort is currently ongoing to improve the reliability of metabolism assays for low clearance compounds. Another area of assay improvement is generating plasma protein binding data for highly bound compounds, which is an issue for several antimalarial compounds. Many antimalarials also have low solubility which leads to formulation challenges and potential cost increases. For legacy compounds where the initial focus was metabolic DDI, the fraction absorbed (*f*_a_) was defined in the PBPK model based on clinical information. However, the prediction of *f*_a_ is clearly important for drugs in development requiring in vitro solubility data so that modeling can be used to support formulation design and reduce the number of clinical studies required to assess formulation. Integrating in vitro studies on formulation in PBPK in this chemical space is underway. This approach has been taken on one of our recent development compounds, artefenomel (manuscript pending), and including these assessments as part of our routine strategy is a future opportunity.

Many antimalarial compounds have very high plasma protein binding, and therefore, may not reach free plasma concentrations which are high enough to inhibit an enzyme clinically. However, owing to uncertainty in measuring *f*_u_ values lower than 0.01 and in *K*_*i*_ determination itself (for example when different probe substrates affect the *K*_*i*_ ^41^ or when there are observed differences between in vitro and in vivo *K*_*i*_), simulations should be accompanied by appropriate sensitivity analyses.^36^

The next step after developing these compound files is to use the most appropriate population to perform DDI simulations. A healthy adult volunteer population is a good place to start, especially when the goal is to inform on the design of a phase I clinical DDI study. However, our ultimate aim is to include predictions of the outcome in patients with malaria using a disease‐specific virtual population incorporating physiological differences observed in patients with malaria, such as reduced CYP3A4 expression and changes in blood binding. To this end, development and verification of a virtual population is underway.

Although the initial focus of this study was DDI assessment, the range of application of the models and our proposed strategy proved to be broader. Figure 5 includes more innovative applications of the models that are currently being conducted at MMV, such as predictions of PKs in special populations relevant to malaria and organ exposures. For example, a number of the models have been applied by us and independent researchers to predict lung concentrations as part of prioritizing candidates for drug re‐purposing to treat COVID‐19.^17^, ^42^, ^43^, ^44^, ^45^ In these cases, existing “off‐the‐shelf” models were evaluated and the mechanistic lung model was incorporated allowing us to rapidly react to the pandemic prioritizing and planning clinical studies. Models are also being used to predict drug exposures in pregnant women and the milk passage in lactating women, in order to support early inclusions of these populations in malaria clinical research and support MMV's Malaria in Mothers and Babies (MiMBa) initiative.^46^

We hope this strategy that has been developed for antimalarial drug development will inspire scientists developing drugs for other global health diseases to use PBPK modeling, as many similarities exist in terms of target populations and DDI risk.

## CONCLUSIONS

This collaborative work has established a repository of PBPK models and developed a strategy for their use to support antimalarial drug development. The suite of drug models can be readily used to address a variety of drug development and clinical application questions to inform optimal drug dosing. We would like to encourage pharmacology and clinical research communities to evaluate and apply these models and, where relevant, to update them once new data become available. We also welcome feedback and sharing of refined models.

## AUTHOR CONTRIBUTIONS

N.A., E.H., and L.M.A. wrote the manuscript. N.A., E.H., K.R.‐Y., N.G., D.W., S.A.C., J.J.M., J.N.B., and L.M.A. designed the research. N.A., E.H., K.R.‐Y., M.D., M.C.B.‐K., K.‐F.C., S.M., J.N.B., L.G.A.S., H.B., Z.B., H.M.J., and L.M.A. performed the research. N.A., E.H., K.R.‐Y., M.D., M.C.B.‐K., K.‐F.C., S.M., J.N.B., L.G.A.S., H.B., Z.B., H.M.J., and L.M.A. analyzed the data.

## FUNDING INFORMATION

This work was funded by the Bill and Melinda Gates Foundation (INV‐019894 and INV‐040110). The views expressed do not reflect official views of the Bill & Melinda Gates Foundation.

## CONFLICT OF INTEREST STATEMENT

E.H., K.R.‐Y., M.C.B.‐K., K.‐F.C., S.M., J.J.B., L.G.A.S., H.B., Z.B., H.M.J., D.W., and L.M.A. are employees of Certara and may hold stocks or shares. At the time of research, M.D. was an employee of Certara. N.A., N.G., J.J.M., and J.N.B. are employees of Medicines for Malaria Venture (MMV). S.A.C. declared no competing interests for this work. As Deputy Editor‐in‐Chief of CPT: Pharmacometrics & Systems Pharmacology, K.R.‐Y. was not involved in the review or decision process for this paper.

## Supporting information


Table S1

Table S2

Table S3

Appendix S1

Appendix S2

Figure S1

Figure S2

Figure S3

Figure S4

Figure S5
