# Supplementary material for: Development and application of a PBPK modeling strategy to support antimalarial drug development
Source: CPT Pharmacometrics Syst Pharmacol. 2023 Aug 16;12(9):1335–46. doi: 10.1002/psp4.13013 (PMC10508484; doi:10.1002/psp4.13013)
Supplement: Supplementary file 1 — Table S1 [file PSP4-12-1335-s009.pdf]

Table S1 PBPK Input parameters

	VALUE	REFERENCE
**Amodiaquine**		
MW	355.86	PubChem (salt MW 464.86)
fu	0.089	Charman et al., 2020 [1]
B:P	1.1	Charman et al., 2020 [1]
Log P	3.51	Calculated from Log D of 2.95 reported by Charman et al., 2020 [1]
Main binding protein	HSA	Hietala, 2009 [2]
Compound type	Diprotic Base	
pKa(s)	7.59, 6.25	Charman et al., 2020 [1]
fa	1	Assumed (fa predicted from Caco-2 data = 0.87, fa predicted from PSA and HBD = 0.93) Yu et al., 1999 [3], Winiwarter et al., 1998 [4]
ka (h^{-1})	0.25	Optimised to recover t_{max}
Q_{gut} (L/h)	9.11	Predicted from Caco-2 data using method previously described Yang et al., 2007 [5]
fu_{gut}	1	Assumed
Caco-2 (P_{app} A-B) 10^{-6} cm/s	9.8	Nair et al., 2012 [6]
V_{ss} (L/kg) (Full PBPK)	14.1	Predicted using Method 2 with Kp scalar, Rodgers et al., 2006 [7]
Kp scalar	1.5	Optimised
CL_{int} CYP2C8 (µL/min/pmol)	290	Derived from *in vivo* clearance (2500 L/h) using retrograde calculations (Equation 3) and assuming fmCYP2C8 of 72 % based on observed DDI study with trimethoprim, Akande et al., 2015 [8]. In vitro estimation of fmCYP2C8 was 90-95 % but this over predicted the clinically observed DDI. The *in vivo* clearance was selected as a reasonable estimate within the clinically observed range, Winstanley et al., 1987 [9]
Additional HLM CL_{int} (µL/min/mg)	2091	
CL renal (L/h)	3.5	Calculated from Winstanley et al., 1987 [9]
CYP2D6 Ki (µM)	0.45	Calculated using the Cheng Prusoff approximation from IC_{50} of 0.88 µM, Charman et al., 2020 [1], Cheng et al., 1973 [10]
fu_{mic} (for CYP2D6 Ki)	0.9	Charman et al., 2020 [1]
**Artemether**		
MW	298.38	PubChem
fu	0.038	Charman et al., 2020 [1]
B:P	0.8	Colussi et al., 1999 [11]
Log P	3.7	Charman et al., 2020 [1]
Main binding protein	HSA	Assumed from physicochemical properties
Compound type	Neutral	
pKa(s)	n/a	
fa	1	Assumed for fed state
ka (h^{-1})	0.5	Optimised to more accurately recover t_{max}
tlag (h)	0.5	Optimised to more accurately recover t_{max}

Q_{gut} (L/h)	15.1	Predicted from Caco-2 data, Yang et al., 2007 [5]; predicted Fg value = 1.0
fu_{gut}	1	Assumed
Caco-2 (P_{app} A-B) 10^{-6} cm/s	42.7	Calibrated against Propranolol (P_{app} A-B 41 x 10^{-6} cm/s), Charman et al., 2020 [1]
V_{ss} (L/kg) (Full PBPK)	0.898	Predicted using Simcyp Method 3 (based on the Rodgers and Rowland method, Rodgers et al., 2006 [7] with additional consideration of the permeation of the ionised form) for the prediction of tissue:plasma partition coefficients) plus optimised adipose Kp = 0.5 and Kp scalar = 0.5
CL renal (L/h)	0	Assumed
CYP2B6 Vmax (pmol/min/pmol)	42.5	Optimised from reported value of 17.9 pmol/min/pmol, Honda et al., 2011 [12], to recover single dose CL/F; fmCYP2B6 = 47% (without induction)
CYP2B6 Km (µM)	1.95	Honda et al., 2011 [12]
CYP3A4 V_{max} (pmol/min/pmol)	31.3	Optimised from reported value of 12.3 pmol/min/pmol, Honda et al., 2011 [12], to recover single dose CL/F; fmCYP3A4 = 53%
CYP3A4 Km (µM)	8.24	Honda et al., 2011 [12]
CYP2B6 Ind_{max} (max fold induction)	500	Optimised to recover *in vivo* exposure
CYP2B6 $IndC_{50}$ (µM) (concentration yielding 50% max induction)	0.1	Optimised to recover *in vivo* exposure
**Atovaquone**		
MW	366.84	PubChem
fu	0.001	Mepron Prescribing Information, 2008, Nixon et al., 2013 [13], Rolan et al., 1997 [14], GlaxoSmithKline, [15]
B:P	0.55	Rolan et al., 1997 [14], Beerahee, 1999 [16], Charman et al., 2020 [1]
Log P	8.4	Calculated from Log D 5.3 Charman et al., 2020 [1] and pKa
Main binding protein	HSA	Nixon et al., 2013 [13]
Compound type	Monoprotic Acid	
pKa	4.28	Charman et al., 2020 [1]
fa	0.24	Oral bioavailability, Lavelle et al., 1994 [17]
ka (h^{-1})	1	Optimised to capture t_{max}
Tlag (h)	1.5	Optimised to capture t_{max} (CV 15%)
Q_{gut} (L/h)	10.6	Predicted from Caco-2 data, Yang et al., 2007 [5]
fu_{gut}	1	Assumed
Caco-2 (P_{app} A-B) 10^{-6} cm/s	300	Calibrated against Propranolol (P_{app} A-B 101 x 10^{-6} cm/s) using plasma as medium, Charman et al., 2020 [1]. Propranolol values obtained from personal communication
V_{ss} (L/kg) (Full PBPK)	0.88	Predicted Method 2 with Kp scalar, Rodgers et al., 2006 [7]

Kp scalar	0.04	Optimised to recover clinical observed findings, Lavelle et al., 1994 [17]
CL_{int} biliary (µL/min/million cells)	54.04	Derived from *in vivo* clearance (CL IV, 0.62 L/h; Lavelle et al., 1994 [17]) using retrograde calculations (Equation 1, Supplementary Information)
CL renal (L/h)	0	< 0.6% of parent excreted in urine, Rolan et al., 1997 [14]
**Azithromycin**		Previously published in Johnson et al., 2016 [18]; Rowland Yeo et al., 2020 [19]
MW	747.9	PubChem
fu	0.69	Foulds et al., 1990 [20]
B:P	2.28	Based on *in vivo* data, Pene Dumitrescu et al., 2013 [21]
Log P	4.02	PubChem/DrugBank
Main binding protein	AGP	Assumed from physicochemical properties
Compound type	Monoprotic base	
pKa(s)	8.74	PubChem/DrugBank
fa	1	assumed
ka (h^{-1})	0.5	Optimised to recover observed profile
fu_{gut}	1	Assumed
V_{ss} (L/kg) (Full PBPK)	25.4	Predicted via Method 3, Rodgers et al., 2005 [22]
CL_{int} biliary (uL/min/million cells)	9.25	Calculated from CL IV using Simcyp retrograde Model (Equation 2)
CL renal (L/h)	8.67	Lalak et al., 1993 [23]
CYP3A4 K_I (µM)	105	Rowland Yeo et al., 2011 [24]
CYP3A4 K_{inact} (1/hr)	0.61	Rowland Yeo et al., 2011 [24]
fu_{mic} (for CYP3A4 Kapp)	1	Assumed
**Carboxyprimaquine**		
MW	274.32	PubChem
fu	0.04	Predicted from phys chem
B:P	1	Assumed to equal that of parent
Log P	2.66	Calculated, ACD Percepta
Main binding protein	HSA	assumed
Compound type	Ampholyte	
pKa(s)	4.6, 3.4	Calculated, ACD Percepta
fu_{gut}	1	Assumed
V_{ss} (L/kg) (Full PBPK)	0.13	Fitted to *in vivo* data Ward et al., 1985 [25]
Kp Scalar	1	Fitted to *in vivo* data Ward et al., 1985 [25]
CL IV (L/h)	0.5	Fitted to *in vivo* data Ward et al., 1985 [25]
CL renal (L/H)	0	Mihaly et al., 1985 [26]
**Chloroquine** (previously published in Rowland Yeo et al., 2020 [19])		
MW	319.87	PubChem
fu	0.4	Ofori-Adjei et al., 1986 [27], Walker et al., 1983 [28]
B:P	3.5	Charman et al., 2020 [1]
Log P	4.37	Calculated from log D 0.93 Charman et al., 2020 [1] and pKa
Main binding protein	HSA	Assumed from physicochemical properties
Compound type	Diprotic base	

pKa(s)	9.94, 8.40	Charman et al., 2020 [1]
fa	1	Walker et al., 1987 [29]
ka (h^{-1})	0.5	Optimised to recover t_{max} of 4 hours, Walker et al., 1987 [29,] Gustafsson et al., 1983 [30]
Q_{gut} (L/h)	17.4	Predicted from PSA (25.69) and HBD (1) Winiwarter et al., 1998 [4]; predicted Fg = 0.96
fu_{gut}	1	assumed
V_{ss} (L/kg) (Minimal PBPK)	85.7	Predicted using the Method 3 (based on the Rodgers and Rowland method, Rodgers et al., 2006 [7] with additional consideration of the permeation of the ionised form) for the prediction of tissue:plasma partition coefficients
Sub-Cellular Distribution	ON	For Kidney and Liver
CL_{int} CYP2C8 (µL/min/pmol)	0.27	Derived from CL IV (39.84, Walker et al., 1987 [29,] Gustafsson et al., 1983 [30] using retrograde calculations (Equation 1 & 2) and assuming % Hep CL_{int} 50 %, Kim et al., 2003 [31,] Projean et al., 2003 [32]; fmCYP2C8 = 11%
CL_{int} CYP3A4 (µL/min/pmol)	0.028	Derived using retrograde calculations (Equation 1 & 2) and assuming % Hep CL_{int} 30 %, Kim et al., 2003 [31,] Projean et al., 2003 [32]; fmCYP3A4 = 19%
CL_{int} additional (µL/min/mg protein)	2.58	Derived using retrograde calculations (Equation 1 & 2) and assuming % Hep CL_{int} 20 %, Kim et al., 2003 [31,] Projean et al., 2003 [32]; fm = 8%
CL renal (L/h)	24.78	Walker et al., 1987 [29]
CYP2D6 Ki (µM)	3.15	Calculated from IC_{50}, Charman et al., 2020 [1]
fu_{mic} (for CYP2D6 Ki)	0.797	Extrapolated from a measured fu_{mic} of 0.5 at a protein concentration of 0.4 mg/mL (Charman et al., 2020 [1]) to that of 0.1 mg/mL using the method by Austin et al., 2002 [33]
**Cycloguanil**		
MW	251.72	PubChem
fu	0.75	Charman et al., 2020 [1]
B:P	0.71	Charman et al., 2020 [1]
Log P	1.86	Calculated from Log $D_{pH\ 7.4}$ of 1.1 and pKa, Charman et al., 2020 [1]
Main binding protein	AGP	Assumed from physicochemical properties
Compound type	Monoprotic Base	
pKa	11.4	Charman et al., 2020 [1]
fu_{gut}	1	Assumed
V_{ss} (L/kg) (full PBPK)	0.76	Predicted using Method 2, Rodgers et al., 2006 [7]
CL IV (L/h)	20	Optimised to recover observed cycloguanil plasma concentration-time profiles in EM subjects, Jeppesen et al., 1997 [34]
CL renal (L/h)	0	Assumed in the absence of information
CYP2D6 Ki (µM)	3.7	Charman et al., 2020 [1]
fu_{mic} (for CYP2D6 Ki)	0.911	Extrapolated from a measured fu of 0.72 at protein concentration 0.4 mg/mL (Charman et al., 2020 [1]) to a

		protein concentration of 0.1 mg/mL using the method of Austin et al., 2002 [33]
**DEAQ**		
MW	327.81	PubChem
fu	0.26	unpublished data
B:P	3	Winstanley et al., 1987 [9]
Log P	2.6	Calculated from Log $D_{pH\ 7.4}$ 1.3 and pKa, Charman et al., 2020 [1]
Main binding protein	AGP	Hietala, 2009 [2]
Compound type	Diprotic Base	
pKa(s)	8.48, 7.13	Charman et al., 2020 [1]
fu_{gut}	1	Assumed
V_{ss} (L/kg) (Minimal PBPK)	34.3	Predicted using Rodgers et al., 2006 [7]
CL PO (L/h)	32	Akande et al., 2015 [8,] Scarsi et al., 2014 [35,] Tarning et al., 2012 [36,] Orrell et al., 2008 [37]
CL renal (L/h)	1.4	Winstanley et al., 1987 [9]
CYP2D6 Ki (μM)	1.6	Charman et al., 2020 [1]
fu_{mic} (for CYP2D6 Ki)	0.9	Calculated from phys chem properties for protein concentration 0.4 mg/mL, Turner et al., 2006 [38]
**DHA (administered as artesunate)**		
		Administration of artesunate leads to the rapid conversion to DHA. For simplicity, fa, ka and tlag were modified to recover observed DHA concentrations. All other parameters were the same as for the main DHA file (described below)
fa	1	Relative fa (ratio of *in vivo* fasted AUC / high fat fed AUC reported in Reuter et al., 2015 [39])
ka (h^{-1})	1.75	Optimised to recover the tmax value observed from Diem Thuy et al., 2008 [40]
tlag (h)	0.55	
**DHA (administered as DHA)**		
MW	284.35	
fu	0.105	Healthy volunteers, Batty et al., 2004 [41]
B:P	1	Assumed
Log P	2.3	Charman et al., 2020 [1]
Main binding protein	AGP	Batty et al., 2004 [41]
Compound type	Neutral	
pKa	n/a	
fa	0.71	Relative fa (ratio of *in vivo* fasted AUC / high fat fed AUC that are reported in Reuter et al., 2015 [39])
ka (h^{-1})	0.54	Optimised to recover the typical t_{max} value observed in the fasted state, using initial estimates from Na-Bangchang et al., 2004 [42]
tlag (h)	0.55	Optimised to recover the typical t_{max} value observed in the fasted state, using initial estimates from Na-Bangchang et al., 2004 [42]
Q_{gut} (L/h)	16.6	Predicted from Caco-2 data, Yang et al., 2007 [5]

Caco-2 (P_{app} A-B) 10^{-6} cm/s	49	Calibrated against Propranolol (P_{app} A-B 30 x 10^{-6} cm/s), Charman et al., 2020 [1] Propranolol values obtained from personal communication
fu_{gut}	1	Assumed
V_{ss} (L/kg) (Full PBPK)	0.50	Predicted, Rodgers et al., 2006 [7]
Kp scalar	0.5	Optimised to recover *in vivo* concentration-time profiles Na-Bangchang et al., 2004 [42]
UGT1A9 (µL/min/mg protein)	287.56	Calculated from CL PO (273.2 L/h, Na-Bangchang et al., 2004 [42]) using the Retrograde model (Equation 3) and fmUGT1A9 = 50%, Isozymes identified by Ilett et al., 2002 [43] but in the absence of tissue-specific UGT scalars to scale up to a whole organ CL_{int}, equal contribution was assumed, Ilett et al., 2002 [43]
UGT2B7 (µL/min/mg protein)	287.56	Calculated from CL PO (273.2 L/h, Na-Bangchang et al., 2004 [42]) using the Retrograde model (Equation 3) and fmUGT2B7 = 50%, Isozymes identified by Ilett et al., 2002 [43] but in the absence of tissue-specific UGT scalars to scale up to a whole organ CL_{int}, equal contribution was assumed, Ilett et al., 2002 [43]
UGT tissue scalars liver	1	In the absence of measured tissue scalars, judgement based on the relative expression of enzymes in tissues led to the conclusion that liver was likely to be the major cite of metabolism. The model, therefore, assumes hepatic metabolism for simplicity.
Intestine	0	
Kidney	0	
CL renal (L/h)	0	The excretion of unchanged active substance in human urine is negligible for artemisinin derivatives, EMA Website, [44]
CYP1A2 Ki (µM)	4.38	Charman et al., 2020 [1]
fu_{mic} (for CYP1A2 Ki)	0.964	fu scaled from that measured at 0.4 mg/mL (Charman et al., 2020 [1]) to 0.1 mg/mL using the method of Austin et al., 2002 [33]
**Doxycycline**		
MW	444.43	PubChem
fu	0.23	Charman et al., 2020 [1]
B:P	0.78	Charman et al., 2020 [1]
Log P	2.34	Calculated from measured Log $D_{pH\ 7.4}$ -0.2 and pKa, Charman et al., 2020 [1]
Main binding protein	HSA	Assumed from physicochemical properties
Compound type	Ampholyte	
pKa(s)	pKa 1 (acidic): 4.7, pKa 2 (basic) 7.8	Calculated with ACD/Percepta
fa	0.95	Fabre et al., 1966 [45,] Saivin et al., 1988 [46]
ka (h^{-1})	0.96	Optimised
fu_{gut}	1	Assumed
V_{ss} (L/kg) (Full PBPK)	0.653	Predicted by Simcyp Method 2, Rodgers et al., 2006 [7] and refined using a Kp scalar

Kp scalar	0.3	Fitted to recover observed data, Grahnen et al., 1994 [47]
CL biliary (µL/min/10^6cells)	0.796	Calculated using weighted mean CL IV (2.77 L/h, Raghuram et al., 1982 [48], Nguyen et al., 1989 [49]) the retrograde model within Simcyp (Equations 1 & 2)
CL renal (L/h)	0.87	Weighted mean from IV studies, Raghuram et al., 1982 [48], Nguyen et al., 1989 [49]
Additional CL_{int} (liver) (µL/min/mg protein)	0.033	Calculated using the retrograde model within Simcyp (Equation 1 & 2)
**Lumefantrine**		
MW	528.94	PubChem
fu	0.003	CDER submission 22-269
B:P	0.55	0.55 = no uptake into RBC for a haematocrit of 45%; Charman et al., 2020 [1] measured 0.48
Log P	9.1	Calculated from Log $D_{pH\ 7.4}$ of 6.84 (ACD labs value) and pKa, Charman et al., 2020 [1]
Main binding protein	HSA	Assumed
Compound type	Monoprotic base	
pKa	9.8	Calculated (ACD labs value), Charman et al., 2020 [1]
fa	1	Assumed due to administration with food
ka (h^{-1})	0.35	Optimised to more accurately recover t_{max}
tlag (h)	2	White et al., 1999 [50]
fu_{gut}	1	Assumed
Q_{gut}	17.6	Predicted from physicochemical properties, Winiwarter et al., 1998 [4]; predicted Fg = 0.82
PSA	23.47	PubChem
HBD	1	PubChem
V_{ss} (L/kg) (Full PBPK)	2.18	Predicted using Method 1, Poulin et al., 2002 [51] plus optimised adipose Kp = 7 and Kp scalar = 0.04
CYP3A4 CL_{int} (µL/min/pmol)	0.9	Calculated from CL = 3.5 L/h (Lefevre et al., 2002 [52], Hoglund et al., 2015 [53]) using the retrograde model (Equation 3) and fmCYP3A4 = 40% (required to capture DDI data with ketoconazole)
Additional HLM CL_{int} (µL/min/mg protein)	185	Calculated from CL PO = 3.5 L/h (Lefevre et al., 2002 [52], Hoglund et al., 2015 [53]) using the retrograde model (Equation 3) and fm = 60%
CL renal (L/h)	0	Assumed
CYP2D6 Ki (µM)	1.8	Charman et al., 2020 [1]
fu_{mic} (for CYP2D6 Ki)	0.002	Unpublished data
**Mefloquine**		
MW	378.3	PubChem
fu	0.015	Charman et al., 2020 [1]
B:P	1.1	Charman et al., 2020 [1]
Log P	3.86	Calculated from Log $D_{pH\ 7.4}$ 2.7 and pKa, Charman et al., 2020 [1]
Main binding protein	AGP	Zsila et al., 2008 [54]
Compound type	Monoprotic base	
pKa	8.53	Charman et al., 2020 [1]

fa	0.7	Assumed to be the maximum fa as indicated by Frel (fed AUC/fasted AUC) observed by Crevoisier et al., 1997 [55]
ka (h^{-1})	0.24	Optimised using sensitivity analysis to recover observed t_{max}
Q_{gut} (L/h)	14.6	Predicted from Caco-2 data using method described by Yang et al., 2007 [5]; predicted Fg = 0.95
fu_{gut}	1	Assumed
Caco-2 (P_{app} A-B) 10^{-6} cm/s	66	Calibrated against Propranolol (P_{app} A-B 72 x 10^{-6} cm/s), Charman et al., 2020 [1] Propranolol values obtained from personal communication
V_{ss} (L/kg) (Full PBPK)	6.52	Predicted using Method 2, Rodgers et al., 2006 [7]
CL_{int} CYP3A4 (uL/min/pmol)	0.168	Derived using CL PO (2.09 L/h, Charles et al., 2007 [56]) in retrograde calculations (Equation 3) and assuming % Hep CL_{int} of 100 % (fmCYP3A4 = 95%)
CL renal (L/h)	0.07	Average fe = 5%, Schwartz et al., 1982 [57]
CYP2C9 Ki (µM)	260	Karbwang et al., 1988 [58]
fu_{mic} (for CYP2C9 Ki)	0.026	Scaled for protein content, Charman et al., 2020 [1]
CYP2D6 Ki (µM)	10	Charman et al., 2020 [1]
fu_{mic} (for CYP2D6 Ki)	0.174	Scaled for protein content, Charman et al., 2020 [1]
CYP3A4 Ki (µM)	41	From Grace et al., 1998 [56] (Ki = 41 µM x fu_{mic} 0.095), Charman et al., 2020 [1], Grace et al., 1998 [59]
fu_{mic} (for CYP3A4 Ki)	0.095	Scaled for protein content from measured value, Charman et al., 2020 [1]
**Piperaquine**		
MW	535.52	PubChem
fu	0.006	Unpublished data
B:P	0.9	Unpublished data
Log P	5.27	Calculated, Charman et al., 2020 [1]
Main binding protein	AGP	Assumed from physicochemical properties
Compound type	Diprotic Base	
pKa(s)	8.8, 7.4	Calculated (ChemAxon value), Charman et al., 2020 [1]
fa - 960 mg fasted	0.4	Relative fa *in vivo* fasted AUC / high fat fed AUC) for the 2 doses, EMA Website, [44]
fa - 1280 mg fasted	0.33	
fa - 960 mg fed	1	
fa - 1280 mg fed	1	
ka (h^{-1})	0.6	Optimised to recover observed clinically observed data
tlag (h)	1	
Q_{gut} (L/h)	18.6	Predicted from Caco-2 data, Yang et al., 2007 [5]; predicted Fg = 0.98
Caco-2 (P_{app} A-B) 10^{-6} cm/s	233	Calibrated against Propranolol using plasma as matrix (P_{app} A-B 72 x 10^{-6} cm/s), Charman et al., 2020 [1] Propranolol values obtained from personal communication
fu_{gut}	0.006	Set to equal fu
V_{ss} (L/kg) (Minimal PBPK)	47.4	Predicted with Kp scalar Method 2, Rodgers et al., 2006 [7]
Kp scalar	3	Optimised to recover concentration time profile in rat, Tarning et al., 2008 [60]

CL_{int} CYP3A4 (µL/min/pmol)	25.3	HLM CL_{int} 31.6 µL/min/mg with 80% assigned to CYP3A4 based on chemical inhibition data (unpublished data); thus fmCYP3A4 = 80%
CL_{int} CYP2C9 (µL/min/pmol)	3.16	HLM CL_{int} 31.6 µL/min/mg with 20% split equally between CYP2C9 and CYP2C19, cited as minor enzymes, EMA Website, [44]; thus fmCYP2C9 = 10%, fmCYP2C19 = 10%
CL_{int} CYP2C19 (µL/min/pmol)	3.16	
fu_{mic} (for all CL_{int})	0.013	Measured at 0.4 mg/mL protein concentration, Charman et al., 2020 [1]
CL renal (L/h)	0	Assumed
CYP3A4 Ki (µM)	3.0	Charman et al., 2020 [1]
CYP3A4 k_{inact} (h^{-1})	1.25	Derived from IC_{50} shift data (i.e. using a single time point), (unpublished data)
CYP3A4 K_I (µM)	0.12	
**Primaquine**		
MW	259.35	PubChem
fu	0.26	Charman et al., 2020 [1]
B:P	0.82	Charman et al., 2020 [1]
Log P	3.13	Calculated from experimental Log $D_{pH\ 7.4}$ 0.54 and pKa, Charman et al., 2020 [1]
Main binding protein	AGP	Assumed from physicochemical properties
Compound type	Diprotic Base	
pKa(s)	10.2, 3.3	Charman et al., 2020 [1]
fa – user input	0.98	Fitted to *in vivo* data from Mihaly et al., 1985 [26]
ka (h^{-1}) – user input	0.35	Fitted to *in vivo* data from Mihaly et al., 1985 [26]
Q_{gut} (L/h)	15.2	Predicted from Caco-2, Yang et al., 2007 [5]
Caco-2 (P_{app} A-B) 10^{-6} cm/s	29	Calibrated against Propranolol (P_{app} A-B 27 x 10^{-6} cm/s), Charman et al., 2020 [1] Propranolol values obtained from personal communication.
fu_{gut}	1	Assumed
V_{ss} (L/kg) (Full PBPK)	1.15	Optimised to recover *in vivo* data from Mihaly et al., 1985 [26]
Kp Scalar	0.55	Optimised to recover *in vivo* data from Mihaly et al., 1985 [26]
CYP2D6 CL_{int} (µL/min/pmol)	0.496	Calculated from CL IV (24.2 L/h, Mihaly et al., 1985 [26]) using the Retrograde model (Equation 1 & 2) and fmCYP2D6 = 11%, Pybus et al., 2012 [61]
MAO (µL/min/mg)	30.8	Calculated using the Retrograde model (Equation 1 & 2), fmMAO = 89%. User UGT used as a surrogate entry
CL renal (L/h)	0	fe <0.01%, White, 1985 [62]
CYP1A2 Ki (µM)	0.09	IC_{50} < 0.25 µM, Charman et al., 2020 [1]. Ki was calculated using the Cheng Prusoff approximation and assuming IC_{50} = 0.25uM Cheng et al., 1973 [10]
fu_{mic} (for CYP1A2 Ki)	0.84	fu_{mic} scaled to 0.1 mg/mL from that measured at 0.4 mg/mL, Charman et al., 2020 [1] using the method described by Austin et al., 2002 [33]
**Proguanil**		
MW	253.73	PubChem
fu	0.25	Wattanagoon et al., 1987 [63]

B:P	4.1	Weighted mean of data from Helsby et al., 1993 [61] and Wattanagoon et al., 1987 [63]
Log P	3.19	Calculated from Log $D_{pH\ 7.4}$ 0.27 nad pKa, Charman et al., 2020 [1]
Main binding protein	AGP	Assumed from physicochemical properties
Compound type	Diprotic Base	
pKa(s)	11.1, 2.1	Calculated using ACD Percepta
fa	1	Ke et al., 2014 [64]
ka (h^{-1})	0.417	Optimised to recover clinically observed t_{max}
tlag (h)	0.5	Optimised to recover clinically observed t_{max}
Q_{gut} (L/h)	6.21	Predicted from Caco-2 data, Charman et al., 2020 [1]; predicted Fg = 0.90
Caco-2 (P_{app} A-B) 10^{-6} cm/s	3.5	Calibrated against Propranolol (P_{app} A-B 35 x 10^{-6} cm/s), Charman et al., 2020 [1] Propranolol values obtained from personal communication
fu_{gut}	1	Assumed
V_{ss} (L/kg) (Full PBPK)	9.17	Predicted with modified Kp scalar, Rodgers et al., 2006 [7]
Kp scalar	0.228	Optimised to recover *in vivo* concentration-time profiles, Jeppesen et al., 1997 [34]
CL_{int} CYP2C19-mediated formation of Cycloguanil (µL/min/pmol)	2.23	Derived using CL PO (86.4 L/h, Jeppesen et al., 1997 [34]) in retrograde calculations (Equation 3) and assuming fmCYP2C19 = 17% (% Hep CL_{int} 27.6 %) derived from observed differences in metabolite formation and residual clearance, Jeppesen et al., 1997 [34]
CL_{int} CYP2C19-mediated formation of 4-Chlorphenylbiguanide (CPBG) (µL/min/pmol)	1.07	Derived using retrograde calculations (Equation 3) and assuming fmCYP2C19 = 8% (% Hep CL_{int} 13.2 %) derived from observed differences in metabolite formation and residual clearance, Jeppesen et al., 1997 [34]
CL_{int} CYP2C19 - additional (µL/min/pmol)	1.25	Derived using retrograde calculations (Equation 3) and assuming fmCYP2C19 = 9 % (% Hep CL_{int} 15.5 %) derived from observed differences in metabolite formation and residual clearance, Jeppesen et al., 1997 [34]; total fmCYP2C19 (all routes) = 36%
CL_{int} CYP3A4 formation of cycloguanil (µL/min/pmol)	0.013	Derived using retrograde calculations (Equation 3) and assuming fmCYP3A4 = 3 % (% Hep CL_{int} 5.2 %) derived from observed differences in metabolite formation and residual clearance, Jeppesen et al., 1997 [34]
Additional Undefined metabolism (µL/min/pmol)	13.46	Derived using retrograde calculations (Equation 3) and assuming fm = 25 % (% Hep CL_{int} 38.5 %) derived from observed differences in metabolite formation and residual clearance Jeppesen et al., 1997 [34]
CL renal (L/h)	26.64	Fe = 36% (extensive metabolisers), Jeppesen et al., 1997 [34]
CYP2D6 Ki (µM)	2.2	Charman et al., 2020 [1]
fu_{mic} (for CYP2D6 Ki)	0.66	Extrapolated from measured value at protein concentration 0.4 mg/mL, Charman et al., 2020 [1] to that of 0.1 mg/mL using the method described by Austin et al., 2002 [33]
**Pyrimethamine**		
MW	248.71	PubChem

fu	0.095	Charman et al., 2020 [1]
B:P	0.84	Charman et al., 2020 [1]
Log P	2.52	Calculated from Log $D_{pH\ 7.4}$ 2.41 and pKa, Charman et al., 2020 [1]
Main binding protein	AGP	Assumed from physicochemical properties
Compound type	Monoprotic Base	
pKa	6.86	Charman et al., 2020 [1]
fa	0.25	Fitted to clinically observed data, Cavallito et al., 1978 [65]
ka (h^{-1})	1.01 (CV 38%)	Ahmad et al., 1980 [66]
Q_{gut} (L/h)	17.2	Predicted from Caco-2 permeability, Yang et al., 2007 [5]
Caco-2 (P_{app} A-B) 10^{-6} cm/s	59.8	Calibrated against Propranolol (P_{app} A-B 35 x 10^{-6} cm/s), Charman et al., 2020 [1] Propranolol values obtained from personal communication
fu_{gut}	1	Assumed
V_{ss} (L/kg) (Full PBPK)	0.563	Predicted with modified Kp scalar, Rodgers et al., 2006 [7]
Kp scalar	0.5	Fitted to clinically observed data, Cavallito et al., 1978 [65]
CL_{int} (µL/min/mg)	0.26	Optimised (7-fold reduction from experimentally determined value 1.8 µL/min/mg) to recover clinically observed profile, Almond et al., 2000 [67]
fu_{mic} (for metabolism)	0.54	Charman et al., 2020 [1]
CL renal (L/h)	0.0989	Cavallito et al., 1978 [65]
OCT1 Ki (µM)	2.3	Ito et al., 2010 [68,] Panfen et al., 2019 [69] (average)
OCT2 Ki (µM)	10	Kusuhara et al., 2011 [70]
MATEs Ki (µM)	0.124	Miyake et al., 2021 [71] (average of MATE1 and MATE2-Ki)
**Pyronaridine**		
MW	518.03	PubChem
fu	0.058	Unpublished data
B:P	1.5	Unpublished data
Log P	4.52	Calculated from Log $D_{pH\ 7.4}$ 0.23 and pKa, Charman et al., 2020 [1]
Main binding protein	AGP	Assumed from physicochemical properties
Compound type	Diprotic Base	
pKa(s)	10.2, 9.2	Calculated (ChemAxon value), Charman et al., 2020 [1]
fa	0.74	1st order model – entered value derived from mass balance study (15% coefficient of variation) Morris et al., 2015 [72]
ka (h^{-1})	1.08	Fitted, Jittamala et al., 2015 [73]
PSA/HBD	79/2	Charman et al., 2020 [1]
Q_{gut} (L/h)	6.7	Predicted from PSA and HBD, Winiwarter et al., 1998 [4]; predicted Fg = 0.79
fu_{gut}	1	assumed
Vss (L/kg) (Full PBPK)	78.0	Predicted using the Method 3 (based on the Rodgers and Rowland method, Rodgers et al., 2006 [7] with additional consideration of the permeation of the ionised form) for the prediction of tissue:plasma

		partition coefficients. Subcellular distribution switched on
Kp muscle	182	Optimised to recover the concentration time profiles published by Jittamala et al., 2015 [73]
Kp scalar	0.7	
CL_{int} CYP1A2 (µL/min/pmol)	0.09	Derived using CL PO, blood (22.1 L/h, Jittamala et al., 2015 [73]) in retrograde calculations (Equation 3) and assuming CYP1A2 % Hep CL_{int} of 4.7 % derived *in vitro* using rCYP; fmCYP1A2 = 4.7%
CL_{int} CYP2B6 (µL/min/pmol)	0.47	Derived using CL PO, blood (22.1 L/h, Jittamala et al., 2015 [73]) in retrograde calculations (Equation 3) and assuming CYP2B6 % Hep CL_{int} of 7.8 % derived *in vitro* using rCYP; fmCYP2B6 = 7.7%
CL_{int} CYP2C8 (µL/min/pmol)	0.19	Derived using CL PO, blood (22.1 L/h, Jittamala et al., 2015 [73]) in retrograde calculations (Equation 3) and assuming CYP2C8 % Hep CL_{int} of 4.9 % derived *in vitro* using rCYP; fmCYP2C8 = 4.8%
CL_{int} CYP2D6 (µL/min/pmol)	1.33	Derived using CL PO, blood (22.1 L/h, Jittamala et al., 2015 [73]) in retrograde calculations (Equation 3) and assuming CYP2D6 % Hep CL_{int} of 20.8 % derived *in vitro* using rCYP; fmCYP2D6 = 20.6%
CL_{int} CYP3A4 (µL/min/pmol)	0.42	Derived using CL PO, blood (22.1 L/h, Jittamala et al., 2015 [73]) in retrograde calculations (Equation 3) and assuming CYP3A4 % Hep CL_{int} of 61.7 % derived *in vitro* using rCYP; fmCYP3A4 = 61.1%
CL renal (L/h)	0.16	Fe = 1%, Wattanavijitkul, 2010 [74]
CYP2D6 Ki (µM)	0.15	Optimised based on interaction with metoprolol, Morris et al., 2014 [75]
fu_{mic} (for CYP2D6 Ki)	0.62	Measured
P-gp Ki (µM)	32.7	Equal to IC_{50}, unpublished data<br>Users should also assess DDI when IC_{50} is reduced 15-fold due to potential differences between *in vitro* and *in vivo* IC_{50}, Howgate et al., [76]
**Quinine**		
MW	324.42	PubChem
fu	0.37	Charman et al., 2020 [1]
B:P	0.67	Charman et al., 2020 [1]
Log P	2.95	Calculated from Log $D_{pH\ 7.4}$ 1.8 and pKa, Charman et al., 2020 [1]
Main binding protein	AGP	Assumed based on physicochemical properties
Compound type	Diprotic base	
pKa(s)	8.49, 4.15	Charman et al., 2020 [1]
fa	0.99	Predicted from Caco-2 permeability data, Yu et al., 1999 [3]
ka (h^{-1})	5.7	Predicted from Caco-2 permeability data, Yu et al., 1999 [3]
Caco-2 (P_{app} A-B) 10^{-6} cm/s	39	Charman et al., 2020 [1]
fu_{gut}	1	Assumed
Q_{gut}	16.37	Predicted from Caco-2 permeability data, Yang et al., 2007 [5]; predicted Fg = 0.99

V_{ss} (L/kg) (Minimal PBPK)	2.98	Predicted in Simcyp using Method 1, Poulin et al., 2002 [51]
CL_{int} CYP3A4 µL/min/pmol)	0.043	Calculated from clinically observed CL IV (8.4 L/h White et al., 1983 [77,] Paintaud et al., 1993 [78]) using retrograde approach (Equation 1 & 2) and assuming; fmCYP3A4 = 45% (% Hep CL_{int} = 50 %) based on observed DDI study with troleandomycin, Wanwimolruk et al., 2002 [79]. *In vitro* (Mirghani et al., 2002 [80]) CYP3A4 was 87% of hepatic CL_{int} (fmCYP3A4 = 78%) but this over predicted the clinically observed DDIs.
HLM CL_{int} (µL/min/mg protein)	5.48	Calculated from clinically observed CL IV (8.4 L/h White et al., 1983 [77,] Paintaud et al., 1993 [78]) using retrograde approach (Equation 1 & 2)
CL renal (L/h)	0.89	Wanwimolruk et al., 2002 [79,] Ho et al., 1999 [81,] Mirghani et al., 1999 [82]
CYP2D6 Ki (µM)	2.6	Calculated from IC_{50} of 6 µM, Charman et al., 2020 [1] using Cheng-Prusoff equation Cheng et al., 1973 [10]
**Sulfadoxine**		
MW	310.3	PubChem
fu	0.036	Charman et al., 2020 [1]
B:P	0.57	Charman et al., 2020 [1]
Log P	0.54	Calculated from Log $D_{pH\ 7.4}$ -0.78 and pKa, Charman et al., 2020 [1]
Main binding protein	HSA	Assumed from physicochemical properties
Compound type	Monoprotic Acid	
pKa	6.2	Charman et al., 2020 [1]
fa	0.92	Predicted from Caco-2 data, Charman et al., 2020 [1]
ka (h^{-1}) -	0.90	Predicted from Caco-2 data, Yu et al., 1999 [3]
Q_{gut} (L/h)	10.2	Predicted from Caco-2 data, Yang et al., 2007 [5]
Caco-2 (P_{app} A-B) 10^{-6} cm/s	15	Calibrated against Propranolol (P_{app} A-B 35 x 10^{-6} cm/s), Charman et al., 2020 [1] Propranolol values obtained from personal communication
fu_{gut}	1	Assumed
V_{ss} (L/kg) (Minimal PBPK)	0.14	Predicted using Method 2 with modified Kp scalar, Rodgers et al., 2006 [7]
CL_{int} HLM (µL/min/mg)	0.03	Calculated using CL_{PO} (0.039 L/h, Edstein, 1987 [83]) and assuming fe = 90 %; thus fm = 10%
CL renal (L/h)	0.035	Edstein, 1987 [83]
**Tafenoquine**		Published in Dow et al., 2020 [84]
MW	463.49	PubChem
fu	0.005	Dow et al., 2020 [84]
B:P	1.3	Charman et al., 2020 [1]
Log P	5.61	Calculated from Log $D_{pH\ 7.4}$ 4.24 and pKa Charman et al., 2020 [1]
Main binding protein	HSA	Also AGP, Zsila et al., 2008 [54]
Compound type	Diprotic base	
pKa(s)	8.74, 6.0	Charman et al., 2020 [1]
fa	1	Assumed from fasted vs. fed

ka (h^{-1})	0.1	Fitted from clinical data
fu_{gut}	1	Assumed
V_{ss} (L/kg) (Minimal PBPK)	19.2	Predicted using Simcyp Method 3 (based on the Rodgers and Rowland method, Rodgers et al., 2006 [7] with additional consideration of the permeation of the ionised form) for the prediction of tissue:plasma partition coefficients.
Kp scalar	1.5	Fitted based on clinical data
CL_{int} (uL/min/mg protein)	170.3	Derived using CL PO (3.08 L/h, Green et al., 2016 [85]) in retrograde calculations (Equation 3)
CL renal (L/h)	0	Assumed in the absence of information
CYP2C9 Ki (µM)	10	Charman et al., 2020 [1]
CYP3A4 Ki (M)	2.3	Charman et al., 2020 [1]
**PROPRIETARY COMPOUNDS**		
**DSM265**		
MW	415.33	
fu	0.001	Unpublished MMV in house data (Investigators Brochure)
B:P	0.70	Unpublished MMV in house data
Log P	4.03	Charman et al., 2020 [1]
Main binding protein	HSA	Assumed
Compound type	Neutral	
fa	0.7	Dose specific fa should be used based on those derived from ascending dose studies<br>Fa = 0.7 for doses 400 mg and above, fa = 1 for doses 25 – 250 mg, McCarthy et al., 2017 [86]
ka (h^{-1})	1	Optimised to recover clinically observed t_{max}, McCarthy et al., 2017 [86]
Q_{gut} (L/h)	16.6	Predicted from Caco-2 data, Yang et al., 2007 [5]
Caco-2 (P_{app} A-B) 10^{-6} cm/s	61	Calibrated against Propranolol (P_{app} A-B 36 x 10^{-6} cm/s), Charman et al., 2020 [1]
fu_{gut}	1	assumed
V_{ss} (L/kg) (Minimal PBPK)	0.61	Predicted, Rodgers et al., 2006 [7]
Single Adjusting Compartment (SAC) kin (h^{-1})	0.09	Fitted to clinical data (25 mg arm) McCarthy et al., 2017 [86]
SAC kout (h^{-1})	0.33	
Vsac (L/kg)	0.26	
CL_{int} CYP2C8 (µL/min/pmol)	2.384	Unpublished MMV in house data (estimated fmCYP2C8=70%; fmCYP2C19=30%)
CL_{int} CYP2C19 (µL/min/pmol)	1.751	
CL renal (L/h)	0	
CYP2C8 Ki (µM)	3.10	Unpublished MMV in house data
fu_{mic}	0.44	extrapolated from a measured value at protein concentration of 0.4mg/mL to that at protein 0.2 mg/mL using method by Austin et al., 2002 [33]
CYP2D6 Ki (µM)	4.4	Charman et al., 2020 [1]

fu_{mic}	0.61	extrapolated from a measured value at protein concentration of 0.4mg/mL to that at protein 0.1 mg/mL using method by Austin et al., 2002 [33]
**DSM450**		
MW	431.24	
fu	0.004	Unpublished in-house data
B:P	0.678	Unpublished in-house data
Log P	3.87	Calculated
Main binding protein	HSA	Assumed
Compound type	Neutral	
V_{ss} (L/kg) (Minimal PBPK)	1.46	Predicted using Method 2, Rodgers et al., 2006 [7]
CL PO	0.85	Fitted to clinical data (400 mg SD), McCarthy et al., 2017 [86]
CL renal (L/h)	0	Assumed
CYP2C8 Ki (μM)	4.46	Unpublished in-house data
fu_{mic} for CYP2C8	0.72	Calculated for a protein concentration of 0.2 mg/mL Turner et al., 2006 [38]
UGT1A1 Ki (μM)	0.98	Unpublished in-house data
fu_{mic} for UGT1A1	0.91	Calculated for a protein concentration of 0.05mg/mL Turner et al., 2006 [38]
**MMV048**		
MW	393.4	
fu	0.145	Unpublished MMV in-house data
B:P	0.9	Unpublished MMV in-house data
Log P	2.5	Calculated from Log $D_{pH\ 7.4}$ of 2.50 and pKa Charman et al., 2020 [1]
Main binding protein	HSA	Assumed
Compound type	Base	
pKa	4.0	Charman et al., 2020 [1]
fa	0.82	Optimised
ka (h^{-1})	2	Fitted to clinical data
Q_{gut} (L/h)	16.6	Predicted from Caco-2, Charman et al., 2020 [1]
Caco-2 (P_{app} A-B) 10^{-6} cm/s	44	Calibrated against Propranolol- in-house unpublished data
fu_{gut}	1	Assumed
V_{ss} (L/kg) (Minimal PBPK)	1.87	Predicted, Rodgers et al., 2006 [7]
SAC kin (h^{-1})	0.05	Fitted to in-house clinical data Sinxadi et al., 2020 [87] describing PL of MMV048 following a single oral 40 mg dose)
SAC kout (h^{-1})	0.10	
Vsac (L/kg)	0.42	
CL_{int} CYP3A4 (μL/min/pmol)	0.13	Derived from CL PO 0.82 L/h Sinxadi et al., 2020 [87] and fm CYP3A4 = 10% using retrograde calculations (Equation 3)
CL_{int} UGT1A1 (μL/min/pmol)	1.13	Derived from CL PO 0.82 L/h Sinxadi et al., 2020 [87] and fm CYP3A4 = 90% using retrograde calculations (Equation 3)
CL renal (L/h)	0	0.2 % of dose recovered in rat urine after IV dose

References

1. Charman SA, Andreu A, Barker H, et al. An in vitro toolbox to accelerate anti-malarial drug discovery and development. *Malar J.* 2020;19:1.

2. Hietala SF. Clinical pharmacokinetics and pharmacodynamics of antimalarial combination therapy. PhD thesis, University of Gothenburg, Intellecta Infolog AB, V Frölunda, Sweden, 2009.

3. Yu LX, Amidon GL. A compartmental absorption and transit model for estimating oral drug absorption. *Int J Pharm.* 1999;186:119-125.

4. Winiwarter S, Bonham NM, Ax F, et al. Correlation of human jejunal permeability (in vivo) of drugs with experimentally and theoretically derived parameters. A multivariate data analysis approach. *J Med Chem.* 1998;41:4939-4949.

5. Yang J, Jamei M, Yeo KR, Tucker GT, Rostami-Hodjegan A. Prediction of intestinal first-pass drug metabolism. *Curr Drug Metab.* 2007;8:676-684.

6. Nair A, Abrahamsson B, Barends DM, et al. Biowaiver monographs for immediate release solid oral dosage forms: amodiaquine hydrochloride. *J Pharm Sci.* 2012;101:4390-4401.

7. Rodgers T, Rowland M. Physiologically based pharmacokinetic modelling 2: predicting the tissue distribution of acids, very weak bases, neutrals and zwitterions. *J Pharm Sci.* 2006;95:1238-1257.

8. Akande AA, Olugbenga SJ, Adebanjo AJ, Toyin ASa, Ogbona OC. Effects of co-trimoxazole co-administration on the pharmacokinetics of amodiaquine in healthy volunteers. *International Journal of Pharmacy and Pharmaceutical Sciences.* 2015;7:272-276.

9. Winstanley P, Edwards G, Orme M, Breckenridge A. The disposition of amodiaquine in man after oral administration. *Br J Clin Pharmacol.* 1987;23:1-7.

10. Cheng Y, Prusoff WH. Relationship between the inhibition constant (K1) and the concentration of inhibitor which causes 50 per cent inhibition (I50) of an enzymatic reaction. *Biochem Pharmacol.* 1973;22:3099-3108.

11. Colussi D, Parisot C, Legay F, Lefevre G. Binding of artemether and lumefantrine to plasma proteins and erythrocytes. *Eur J Pharm Sci.* 1999;9:9-16.

12. Honda M, Muroi Y, Tamaki Y, et al. Functional characterization of CYP2B6 allelic variants in demethylation of antimalarial artemether. *Drug Metab Dispos.* 2011;39:1860-1865.

13. Nixon GL, Moss DM, Shone AE, et al. Antimalarial pharmacology and therapeutics of atovaquone. *J Antimicrob Chemother.* 2013;68:977-985.

14. Rolan PE, Mercer AJ, Tate E, Benjamin I, Posner J. Disposition of atovaquone in humans. *Antimicrob Agents Chemother.* 1997;41:1319-1321.

15. GlaxoSmithKline. Mepron® (Atovaquone) Suspension: Prescribing Information. https://www.accessdata.fda.gov/drugsatfda_docs/label/2008/020500s010lbl.pdf. Accessed December 1, 2022

16. Beerahee M. Clinical pharmacology of atovaquone and proguanil hydrochloride. *J Travel Med.* 1999;6 Suppl 1:S13-17.

17. Lavelle J, Trapnell C, Byrne R, et al. The absolute bioavailability of atovaquone tablets and suspension in HIV-Seropositive Volunteers. *Clin Pharmacol Ther.* 1994;55:192.

18. Johnson TN, Jamei M, Rowland-Yeo K. How Does In Vivo Biliary Elimination of Drugs Change with Age? Evidence from In Vitro and Clinical Data Using a Systems Pharmacology Approach. *Drug Metab Dispos.* 2016;44:1090-1098.

19. Rowland Yeo K, Zhang M, Pan X, et al. Impact of Disease on Plasma and Lung Exposure of Chloroquine, Hydroxychloroquine and Azithromycin: Application of PBPK Modeling. *Clin Pharmacol Ther.* 2020;108:976-984.

20. Foulds G, Shepard RM, Johnson RB. The pharmacokinetics of azithromycin in human serum and tissues. *J Antimicrob Chemother.* 1990;25 Suppl A:73-82.

21. Pene Dumitrescu T, Anic-Milic T, Oreskovic K, et al. Development of a population pharmacokinetic model to describe azithromycin whole-blood and plasma concentrations over time in healthy subjects. *Antimicrob Agents Chemother.* 2013;57:3194-3201.

22. Rodgers T, Leahy D, Rowland M. Physiologically based pharmacokinetic modeling 1: predicting the tissue distribution of moderate-to-strong bases. *J Pharm Sci.* 2005;94:1259-1276.

23. Lalak NJ, Morris DL. Azithromycin clinical pharmacokinetics. *Clin Pharmacokinet.* 1993;25:370-374.

24. Rowland Yeo K, Walsky RL, Jamei M, Rostami-Hodjegan A, Tucker GT. Prediction of time-dependent CYP3A4 drug-drug interactions by physiologically based pharmacokinetic modelling: impact of inactivation parameters and enzyme turnover. *Eur J Pharm Sci.* 2011;43:160-173.

25. Ward SA, Mihaly GW, Edwards G, et al. Pharmacokinetics of primaquine in man. II. Comparison of acute vs chronic dosage in Thai subjects. *Br J Clin Pharmacol.* 1985;19:751-755.

26. Mihaly GW, Ward SA, Edwards G, et al. Pharmacokinetics of primaquine in man. I. Studies of the absolute bioavailability and effects of dose size. *Br J Clin Pharmacol.* 1985;19:745-750.

27. Ofori-Adjei D, Ericsson O, Lindstrom B, Sjoqvist F. Protein binding of chloroquine enantiomers and desethylchloroquine. *Br J Clin Pharmacol.* 1986;22:356-358.

28. Walker O, Birkett DJ, Alvan G, Gustafsson LL, Sjoqvist F. Characterization of chloroquine plasma protein binding in man. *Br J Clin Pharmacol.* 1983;15:375-377.

29. Walker O, Salako LA, Alvan G, Ericsson O, Sjoqvist F. The disposition of chloroquine in healthy Nigerians after single intravenous and oral doses. *Br J Clin Pharmacol.* 1987;23:295-301.

30. Gustafsson LL, Walker O, Alvan G, et al. Disposition of chloroquine in man after single intravenous and oral doses. *Br J Clin Pharmacol.* 1983;15:471-479.

31. Kim KA, Park JY, Lee JS, Lim S. Cytochrome P450 2C8 and CYP3A4/5 are involved in chloroquine metabolism in human liver microsomes. *Arch Pharm Res.* 2003;26:631-637.

32. Projean D, Baune B, Farinotti R, et al. In vitro metabolism of chloroquine: identification of CYP2C8, CYP3A4, and CYP2D6 as the main isoforms catalyzing N-desethylchloroquine formation. *Drug Metab Dispos.* 2003;31:748-754.

33. Austin RP, Barton P, Cockroft SL, Wenlock MC, Riley RJ. The influence of nonspecific microsomal binding on apparent intrinsic clearance, and its prediction from physicochemical properties. *Drug Metab Dispos.* 2002;30:1497-1503.

34. Jeppesen U, Rasmussen BB, Brosen K. Fluvoxamine inhibits the CYP2C19-catalyzed bioactivation of chloroguanide. *Clin Pharmacol Ther.* 1997;62:279-286.

35. Scarsi KK, Fehintola FA, Ma Q, et al. Disposition of amodiaquine and desethylamodiaquine in HIV-infected Nigerian subjects on nevirapine-containing antiretroviral therapy. *J Antimicrob Chemother.* 2014;69:1370-1376.

36. Tarning J, Chotsiri P, Jullien V, et al. Population pharmacokinetic and pharmacodynamic modeling of amodiaquine and desethylamodiaquine in women with Plasmodium vivax malaria during and after pregnancy. *Antimicrob Agents Chemother.* 2012;56:5764-5773.

37. Orrell C, Little F, Smith P, et al. Pharmacokinetics and tolerability of artesunate and amodiaquine alone and in combination in healthy volunteers. *Eur J Clin Pharmacol.* 2008;64:683-690.

38. Turner DB, Rostami-Hodjegan A, Tucker GT, Yeo KR. Prediction of Non-Specific Hepatic Microsomal Binding from Readily Available Physicochemical Properties. *9th European ISSX Meeting*. Manchester, UK; 2006.

39. Reuter SE, Evans AM, Shakib S, et al. Effect of food on the pharmacokinetics of piperaquine and dihydroartemisinin. *Clin Drug Investig.* 2015;35:559-567.

40. Diem Thuy LT, Ngoc Hung L, Danh PT, Na-Bangchang K. Absence of time-dependent artesunate pharmacokinetics in healthy subjects during 5-day oral administration. *Eur J Clin Pharmacol.* 2008;64:993-998.

41. Batty KT, Ilett KF, Davis TM. Protein binding and alpha : beta anomer ratio of dihydroartemisinin in vivo. *Br J Clin Pharmacol.* 2004;57:529-533.

42. Na-Bangchang K, Krudsood S, Silachamroon U, et al. The pharmacokinetics of oral dihydroartemisinin and artesunate in healthy Thai volunteers. *Southeast Asian J Trop Med Public Health.* 2004;35:575-582.

43. Ilett KF, Ethell BT, Maggs JL, et al. Glucuronidation of dihydroartemisinin in vivo and by human liver microsomes and expressed UDP-glucuronosyltransferases. *Drug Metab Dispos.* 2002;30:1005-1012.

44. EMA Website. Eurartesim: EPAR - Public assessment report. https://www.ema.europa.eu/en/documents/assessment-report/eurartesim-epar-public-assessment-report_en.pdf. Accessed December 8, 2022

45. Fabre J, Pitton JS, Kunz JP. Distribution and excretion of doxycycline in man. *Chemotherapy.* 1966;11:73-85.

46. Saivin S, Houin G. Clinical pharmacokinetics of doxycycline and minocycline. *Clin Pharmacokinet.* 1988;15:355-366.

47. Grahnen A, Olsson B, Johansson G, Eckernas SA. Doxycycline carrageenate--an improved formulation providing more reliable absorption and plasma concentrations at high gastric pH than doxycycline monohydrate. *Eur J Clin Pharmacol.* 1994;46:143-146.

48. Raghuram TC, Krishnaswamy K. Pharmacokinetics and plasma steady state levels of doxycycline in undernutrition. *Br J Clin Pharmacol.* 1982;14:785-789.

49. Nguyen VX, Nix DE, Gillikin S, Schentag JJ. Effect of oral antacid administration on the pharmacokinetics of intravenous doxycycline. *Antimicrob Agents Chemother.* 1989;33:434-436.

50. White NJ, van Vugt M, Ezzet F. Clinical pharmacokinetics and pharmacodynamics and pharmacodynamics of artemether-lumefantrine. *Clin Pharmacokinet.* 1999;37:105-125.

51. Poulin P, Theil FP. Prediction of pharmacokinetics prior to in vivo studies. 1. Mechanism-based prediction of volume of distribution. *J Pharm Sci.* 2002;91:129-156.

52. Lefevre G, Carpenter P, Souppart C, et al. Pharmacokinetics and electrocardiographic pharmacodynamics of artemether-lumefantrine (Riamet) with concomitant administration of ketoconazole in healthy subjects. *Br J Clin Pharmacol.* 2002;54:485-492.

53. Hoglund RM, Byakika-Kibwika P, Lamorde M, et al. Artemether-lumefantrine co-administration with antiretrovirals: population pharmacokinetics and dosing implications. *Br J Clin Pharmacol.* 2015;79:636-649.

54. Zsila F, Visy J, Mady G, Fitos I. Selective plasma protein binding of antimalarial drugs to alpha1-acid glycoprotein. *Bioorg Med Chem.* 2008;16:3759-3772.

55. Crevoisier C, Handschin J, Barre J, Roumenov D, Kleinbloesem C. Food increases the bioavailability of mefloquine. *Eur J Clin Pharmacol.* 1997;53:135-139.

56. Charles BG, Blomgren A, Nasveld PE, et al. Population pharmacokinetics of mefloquine in military personnel for prophylaxis against malaria infection during field deployment. *Eur J Clin Pharmacol.* 2007;63:271-278.

57. Schwartz DE, Eckert G, Hartmann D, et al. Single dose kinetics of mefloquine in man. Plasma levels of the unchanged drug and of one of its metabolites. *Chemotherapy.* 1982;28:70-84.

58. Karbwang J, Back DJ, Bunnag D, Breckenridge AM. Inhibition of tolbutamide metabolism by antimalarial drugs. *Southeast Asian J Trop Med Public Health.* 1988;19:235-241.

59. Grace JM, Aguilar AJ, Trotman KM, Peggins JO, Brewer TG. Metabolism of beta-arteether to dihydroqinghaosu by human liver microsomes and recombinant cytochrome P450. *Drug Metab Dispos.* 1998;26:313-317.

60. Tarning J, Lindegardh N, Sandberg S, et al. Pharmacokinetics and metabolism of the antimalarial piperaquine after intravenous and oral single doses to the rat. *J Pharm Sci.* 2008;97:3400-3410.

61. Pybus BS, Sousa JC, Jin X, et al. CYP450 phenotyping and accurate mass identification of metabolites of the 8-aminoquinoline, anti-malarial drug primaquine. *Malar J.* 2012;11:259.

62. White NJ. Clinical pharmacokinetics of antimalarial drugs. *Clin Pharmacokinet.* 1985;10:187-215.

63. Wattanagoon Y, Taylor RB, Moody RR, et al. Single dose pharmacokinetics of proguanil and its metabolites in healthy subjects. *Br J Clin Pharmacol.* 1987;24:775-780.

64. Ke AB, Nallani SC, Zhao P, Rostami-Hodjegan A, Unadkat JD. Expansion of a PBPK model to predict disposition in pregnant women of drugs cleared via multiple CYP enzymes, including CYP2B6, CYP2C9 and CYP2C19. *Br J Clin Pharmacol.* 2014;77:554-570.

65. Cavallito JC, Nichol CA, Brenckman WD, Jr., et al. Lipid-soluble inhibitors of dihydrofolate reductase. I. Kinetics, tissue distribution, and extent of metabolism of pyrimethamine, metoprine, and etoprine in the rat, dog, and man. *Drug Metab Dispos.* 1978;6:329-337.

66. Ahmad RA, Rogers HJ. Pharmacokinetics and protein binding interactions of dapsone and pyrimethamine. *Br J Clin Pharmacol.* 1980;10:519-524.

67. Almond DS, Szwandt IS, Edwards G, Lee MG, Winstanley PA. Disposition of intravenous pyrimethamine in healthy volunteers. *Antimicrob Agents Chemother.* 2000;44:1691-1693.

68. Ito S, Kusuhara H, Kuroiwa Y, et al. Potent and specific inhibition of mMate1-mediated efflux of type I organic cations in the liver and kidney by pyrimethamine. *J Pharmacol Exp Ther.* 2010;333:341-350.

69. Panfen E, Chen W, Zhang Y, et al. Enhanced and Persistent Inhibition of Organic Cation Transporter 1 Activity by Preincubation of Cyclosporine A. *Drug Metab Dispos.* 2019;47:1352-1360.

70. Kusuhara H, Ito S, Kumagai Y, et al. Effects of a MATE protein inhibitor, pyrimethamine, on the renal elimination of metformin at oral microdose and at therapeutic dose in healthy subjects. *Clin Pharmacol Ther.* 2011;89:837-844.

71. Miyake T, Kimoto E, Luo L, et al. Identification of Appropriate Endogenous Biomarker for Risk Assessment of Multidrug and Toxin Extrusion Protein-Mediated Drug-Drug Interactions in Healthy Volunteers. *Clin Pharmacol Ther.* 2021;109:507-516.

72. Morris CA, Dueker SR, Lohstroh PN, et al. Mass balance and metabolism of the antimalarial pyronaridine in healthy volunteers. *Eur J Drug Metab Pharmacokinet.* 2015;40:75-86.

73. Jittamala P, Pukrittayakamee S, Ashley EA, et al. Pharmacokinetic interactions between primaquine and pyronaridine-artesunate in healthy adult Thai subjects. *Antimicrob Agents Chemother.* 2015;59:505-513.

74. Wattanavijitkul T. Population pharmacokinetics of pyronaridine in the treatment of malaria. PhD thesis, The University of Iowa, 2010.

75. Morris CA, Pokorny R, Lopez-Lazaro L, et al. Pharmacokinetic interaction between pyronaridine-artesunate and metoprolol. *Antimicrob Agents Chemother.* 2014;58:5900-5908.

76. Howgate E, Neuhoff S, Rowland-Yeo K. Sensitivity analysis of P-glycoprotein Ki values in dynamic DDI predictions. Page Meeting June 6, 2017; Budapest.

77. White NJ, Chanthavanich P, Krishna S, Bunch C, Silamut K. Quinine disposition kinetics. *Br J Clin Pharmacol.* 1983;16:399-403.

78. Paintaud G, Alvan G, Ericsson O. The reproducibility of quinine bioavailability. *Br J Clin Pharmacol.* 1993;35:305-307.

79. Wanwimolruk S, Paine MF, Pusek SN, Watkins PB. Is quinine a suitable probe to assess the hepatic drug-metabolizing enzyme CYP3A4? *Br J Clin Pharmacol.* 2002;54:643-651.

80. Mirghani RA, Yasar U, Zheng T, et al. Enzyme kinetics for the formation of 3-hydroxyquinine and three new metabolites of quinine in vitro; 3-hydroxylation by CYP3A4 is indeed the major metabolic pathway. *Drug Metab Dispos.* 2002;30:1368-1371.

81. Ho PC, Chalcroft SC, Coville PF, Wanwimolruk S. Grapefruit juice has no effect on quinine pharmacokinetics. *Eur J Clin Pharmacol.* 1999;55:393-398.

82. Mirghani RA, Hellgren U, Westerberg PA, et al. The roles of cytochrome P450 3A4 and 1A2 in the 3-hydroxylation of quinine in vivo. *Clin Pharmacol Ther.* 1999;66:454-460.

83. Edstein MD. Pharmacokinetics of sulfadoxine and pyrimethamine after Fansidar administration in man. *Chemotherapy.* 1987;33:229-233.

84. Dow GS, Luttick A, Fenner J, et al. Tafenoquine inhibits replication of SARS-Cov-2 at pharmacologically relevant concentrations in vitro. *bioRxiv.* 2020;199059.

85. Green JA, Mohamed K, Goyal N, et al. Pharmacokinetic Interactions between Tafenoquine and Dihydroartemisinin-Piperaquine or Artemether-Lumefantrine in Healthy Adult Subjects. *Antimicrob Agents Chemother.* 2016;60:7321-7332.

86. McCarthy JS, Lotharius J, Ruckle T, et al. Safety, tolerability, pharmacokinetics, and activity of the novel long-acting antimalarial DSM265: a two-part first-in-human phase 1a/1b randomised study. *Lancet Infect Dis.* 2017;17:626-635.

87. Sinxadi P, Donini C, Johnstone H, et al. Safety, Tolerability, Pharmacokinetics, and Antimalarial Activity of the Novel Plasmodium Phosphatidylinositol 4-Kinase Inhibitor MMV390048 in Healthy Volunteers. *Antimicrob Agents Chemother.* 2020;64.
