# Supplementary material for: Development and application of a PBPK modeling strategy to support antimalarial drug development
Source: CPT Pharmacometrics Syst Pharmacol. 2023 Aug 16;12(9):1335–46. doi: 10.1002/psp4.13013 (PMC10508484; doi:10.1002/psp4.13013)
Supplement: Supplementary file 2 — Table S2 [file PSP4-12-1335-s002.pdf]

Table S2. Comparison of simulated plasma AUC and C_{max} values to those observed clinically.

Compound (regimen)	C_{max} (ng/mL)			AUC window	AUC (ng/mL·h)			Reference
	Obs	Sim	Sim/Obs	(h)	Obs	Sim	Sim/Obs	
**Amodiaquine**								
600 mg QD (Day 3)[a]	24.6	28.4	1.16	0 – 24	204	251	1.23	Scarsi et al., 2014 [1]
540 mg SD[f]	41.2	47.6	1.16	0 – 48	331	521	1.57	Liu et al., 2014 [2]
600 mg SD[a]	58.0	23.5	0.40	0 – 48	748	227	0.30	Soyinka et al., 2010 [3]
600 mg SD[e]	24.9	22.9	0.92	0 – 24	234	194	0.83	Akande et al., 2015 [4]
	**Within 1.5-fold**		**75%**		**Within 1.5-fold**		**50%**	
	**Within 2-fold**		**75%**		**Within 2-fold**		**75%**	
**Artemether**								
80 mg SD[a,c]	104	80.5	0.77	0 – inf	320	333	1.04	Lefevre et al., 2002 [5]
80 mg SD[a,k]	113	74.7	0.66	0 – inf	408	327	0.80	Lefevre et al., 2013 [6]
80 mg BID (Day 3 PM)[a,c]	30.8	38.5	1.25	62 – 72	61.4	124	2.02	Lefevre et al., 2002 [7]
80 mg BID (Day 3 PM)[a,c]	21.2	35.0	1.65	60 – 72	59.5	150	2.53	Huang et al., 2012 [8]
	**Within 1.5-fold**		**75%**		**Within 1.5-fold**		**50%**	
	**Within 2-fold**		**100%**		**Within 2-fold**		**50%**	
**Atovaquone**								
500 mg SD[a,c]	4.4[#]	3.8[#]	0.9	Unknown	288[#]	214[#]	0.7	Rolan et al., 1994 [9]
250 mg SD[a,c]	3.7[*]	4.5[*]	1.2	0 – inf	295[*]	295[*]	1.0	Thapar et al., 2002 [10]
250 mg QD[a,c]	13.8[*]	13.9[*]	1.0	0 – 24	254[*]	260[*]	1.0	Thapar et al., 2002 [10]
	**Within 1.5-fold**		**100%**		**Within 1.5-fold**		**100%**	
	**Within 2-fold**		**100%**		**Within 2-fold**		**100%**	
**Azithromycin**								
500 mg SD[a]	410	394	0.96	0 – 12	1770	2235	1.26	Foulds et al., 1990 [11]
500 mg BID Day 1 then 500 mg QD (Day 6)[a]	620	500	0.81	0 – 12	3180	3381	1.06	Foulds et al., 1990 [11]
	**Within 1.5-fold**		**100%**		**Within 1.5-fold**		**100%**	
	**Within 2-fold**		**100%**		**Within 2-fold**		**100%**	

Compound (regimen)	C_{max} (ng/mL)			AUC window (h)	AUC (ng/mL·h)			Reference
	Obs	Sim	Sim/Obs		Obs	Sim	Sim/Obs	
**Carboxyprimaquine**								
15 mg SD[a]	NA	NA	NA	0 – 24	15100	14900	0.99	Mihaly et al., 1985 [12]
45 mg SD[a]	NA	NA	NA	0 – 24	39900	44800	1.12	Mihaly et al., 1985 [12]
30 mg SD[g]	959	1700	1.78	0 – inf	44000	53600	1.22	Hanboonkunupakarn et al., 2014 [13]
15 mg SD[g]	736	886	1.20	0 – inf	14200	15900	1.12	Ward et al., 1985 [14]
15 mg QD (Day 14)[g]	1240	1310	1.06	0 – inf	24700	25500	1.03	Ward et al., 1985 [14]
30 mg SD[f]	1040	1740	1.67	0 – inf	47200	50900	1.08	Jittamala et al., 2015 [15]
		**Within 1.5-fold**	**50%**			**Within 1.5-fold**	**100%**	
		**Within 2-fold**	**100%**			**Within 2-fold**	**100%**	
**Chloroquine**								
300 mg infusion[a,c] (over 23.5 mins) SD[a,c]	837	881	1.05	0 – inf	7511	8003	1.07	Gustafsson et al., 1983 [16]
300 mg tablet SD[a,c]	76.0	77.9	1.02	0 – inf	6111	7210	1.18	Gustafsson et al., 1983 [16]
300 mg soln. SD[a,c]	73.0	77.9	1.07	0 – inf	4990	7210	1.44	Gustafsson et al., 1983 [16]
		**Within 1.5-fold**	**100%**			**Within 1.5-fold**	**100%**	
		**Within 2-fold**	**100%**			**Within 2-fold**	**100%**	
**Cycloguanil**								
200 mg SD[a]	41	73	1.78	0 – inf	661	1080	1.63	Wattanagoon et al., 1987 [17]
200 mg SD EM[a]	NA	NA	NA	0 – inf	3396	1938	0.57	Helsby et al., 1990 [18]
200 mg SD PM[a]	NA	NA	NA	0 – inf	6563	3781	0.58	Helsby et al., 1990 [18]
200 mg SD EM[a]	NA	NA	NA	0 – 72	2350	2560	1.09	Helsby et al., 1993 [19]
200 mg QD (Day 14) EM[a]	NA	NA	NA	0 – 24	3170	2600	0.82	Helsby et al., 1993 [19]
200 mg SD PM[a]	NA	NA	NA	0 – 72	983	229	0.23	Helsby et al., 1993 [19]
200 mg QD (Day 14) PM[a]	NA	NA	NA	0 – 24	1190	314	0.26	Helsby et al., 1993 [19]
		**Within 1.5-fold**	**0%**			**Within 1.5-fold**	**29%**	
		**Within 2-fold**	**100%**			**Within 2-fold**	**71%**	

Compound (regimen)	C_{max} (ng/mL)			AUC window (h)	AUC (ng/mL·h)			Reference
	**Obs**	**Sim**	**Sim/Obs**		**Obs**	**Sim**	**Sim/Obs**	
**DEAQ**								
600 mg QD (Day 3)[a]	495	343.26	0.69	0 – 96	14571	17358	1.19	Scarsi et al., 2014 [1]
		**Within 1.5-fold**	**100%**			**Within 1.5-fold**	**100%**	
		**Within 2-fold**	**100%**			**Within 2-fold**	**100%**	
**DHA**								
4 mg/kg SD[f]	360	270	0.81	0 – inf	907	895	0.99	Na-Bangchang et al., 2004 [20]
300 mg SD[f]	646	428	0.63	0 – inf	2018	1316	0.68	Na-Bangchang et al., 1997 [21]
240 mg SD[g]	481	354	0.74	0 – inf	1980	1088	0.55	Le et al., 1999 [22]
120 mg SD[g]	159	145	0.91	0 – inf	370	488	1.32	Chinh et al., 2009 [23]
		**Within 1.5-fold**	**75%**			**Within 1.5-fold**	**75%**	
		**Within 2-fold**	**100%**			**Within 2-fold**	**100%**	
**DHA (administered as artesunate)**								
180 mg SD[f]	523	525	1.00	0 – inf	1400	754	0.54	Jittamala et al., 2015 [15]
		**Within 1.5-fold**	**100%**			**Within 1.5-fold**	**0%**	
		**Within 2-fold**	**100%**			**Within 2-fold**	**100%**	
**Doxycycline**								
100 mg SD[a]	1.75[#]	1.60[#]	0.914	0 – inf	29.9[#]	30.3[#]	1.01	Grahnen et al., 1994 [24]
100 mg SD[a]	NA	NA	NA	0 – inf	40.1[#]	30.8[#]	0.77	Malmborg, 1984 [25]
100 mg SD[a]	NA	NA	NA	0 – inf	37.4[#]	30.8[#]	0.82	Malmborg, 1984 [25]
200 mg SD[a]	2.61[#]	3.26[#]	1.25	0 – inf	40.9[#]	62.3[#]	1.52	Wojcicki et al., 1985 [26]
200 mg q24h[b,g]	3.17[#]	2.89[#]	0.912	0 – 24	32.0[#]	42.1[#]	1.32	Newton et al., 2005 [27]
200 mg q24h[b,g]	4.44[#]	4.05[#]	0.912	144 – 168	48.6[#]	58.6[#]	1.21	Newton et al., 2005 [27]
100 mg q24h[a,i]	3.26[#]	2.26[#]	0.70	312 – 408	37.3[#]	36.3[#]	0.97	Binh et al., 2009 [28]
		**Within 1.5-fold**	**100%**			**Within 1.5-fold**	**83%**	
		**Within 2-fold**	**100%**			**Within 2-fold**	**100%**	

Compound (regimen)	C_{max} (ng/mL)			AUC window (h)	AUC (ng/mL·h)			Reference
	Obs	Sim	Sim/Obs		Obs	Sim	Sim/Obs	
**Lumefantrine**								
480 mg SD[a,c]	7.91[#]	4.87[#]	0.62	0 – inf	207[#]	136[#]	0.66	Lefevre et al., 2002 [5]
480 mg SD[a,h]	8.92[#]	5.27[#]	0.59	0 – 264	236[#]	123[#]	0.52	Lefevre et al., 2013 [6]
480 mg BID (Day 3) PM[a,c]	10[#]	10.1[#]	1.01	62 – 480	383[#]	452[#]	1.18	Lefevre et al., 2002 [7]
480 mg BID (Day 3) PM[a,c]	11.6[#]	9.53[#]	0.82	60 – 324	418[#]	396[#]	0.95	Huang et al., 2012 [8]
	**Within 1.5-fold**		**50%**		**Within 1.5-fold**		**50%**	
	**Within 2-fold**		**100%**		**Within 2-fold**		**100%**	
**Mefloquine**								
250 mg SD[a,c]	0.31[#]	0.32[#]	1.02	0 – inf	116[#]	108[#]	0.93	Schwartz et al., 1982 [29]
1000 mg SD[a,c]	0.96[#]	1.27[#]	1.32	0 – inf	516[#]	430[#]	0.83	Schwartz et al., 1982 [29]
750 mg SD[a,g]	1.18[#]	1.18[#]	1.00	NA	NA	NA	NA	Boudreau et al., 1990 [30]
1500 mg SD[a,g]	1.44[#]	2.37[#]	1.64	NA	NA	NA	NA	Boudreau et al., 1990 [30]
750 mg SD[a,g]	1225	1169	0.95	0 – inf	393500	382473	0.97	Na-Bangchang et al., 1999 [31]
500 mg LD, 250 mg weekly (Day 140)[a,h]	1.42[#]	0.92[#]	0.65	3360 – 3528	174.9[#]	114[#]	0.65	Schwartz et al., 1987 [32]
	**Within 1.5-fold**		**66%**		**Within 1.5-fold**		**75%**	
	**Within 2-fold**		**100%**		**Within 2-fold**		**100%**	
**Piperaquine**								
1280 mg SD[n]	0.19[+]	0.24[+]	1.25	0 – 72	3.01[+]	3.46[+]	1.15	EMA Website, [33]
960 mg QD[o] (fasted, Day 3)	0.505[+]	0.37[+]	0.73	0 – 72	5.38[+]	3.62[+]	0.67	Eurartesim S06, Females <75 kg
960 mg QD[o] (fed, Day 3)	1.27[+]	0.97[+]	0.77	0 – 72	12.5[+]	10.6[+]	0.85	Eurartesim S06, Females <75 kg
960 mg QD[o] (fasted, Day 3)	0.28[+]	0.34[+]	1.11	0 – 72	3.93[+]	3.76[+]	0.96	Eurartesim S06, Males <75 kg
960 mg QD[o] (fed, Day 3)	1.12[+]	0.90[+]	0.80	0 – 72	10.7[+]	10.9[+]	1.02	Eurartesim S06, Males <75 kg
1280 mg QD[n] (fasted)	0.28[+]	0.32[+]	1.15	0 – 72	3.93[+]	3.46[+]	0.88	Eurartesim S06, Males ≥75 kg
1280 mg QD[n] (fed)	1.12[+]	1.05[+]	0.94	0 – 72	10.7[+]	12.9[+]	1.20	Eurartesim S06, Males ≥75 kg EMA Website, [33]
	**Within 1.5-fold**		**100%**		**Within 1.5-fold**		**100%**	
	**Within 2-fold**		**100%**		**Within 2-fold**		**100%**	

Compound (regimen)	C_{max} (ng/mL)			AUC window (h)	AUC (ng/mL·h)			Reference
	Obs	Sim	Sim/Obs		Obs	Sim	Sim/Obs	
**Primaquine**								
15 mg SD[a]	53	42	0.79	0 – inf	500	420	0.84	Mihaly et al., 1985 [12]
45 mg SD[a]	176	126	0.72	0 – inf	1700	1300	0.76	Mihaly et al., 1985 [12]
30 mg SD[g]	128	110	0.86	0 – inf	1130	1123	0.99	Hanboonkunupakarn et al., 2014 [13]
15 mg SD	65	53	0.82	0 – inf	468	509	1.09	Ward et al., 1985 [14]
15 mg QD (Day 14)	66	55	0.83	0 – inf	443	525	1.19	Ward et al., 1985 [14]
30 mg SD[f]	139	107	0.77	0 – inf	1180	1050	0.89	Jittamala et al., 2015 [15]
		**Within 1.5-fold**	**100%**			**Within 1.5-fold**	**100%**	
		**Within 2-fold**	**100%**			**Within 2-fold**	**100%**	
**Proguanil**								
200 mg SD[f,p]	170	176	1.04	0 – inf	2975	2878	0.97	Wattanagoon et al., 1987 [17]
200 mg SD EM[a,p]	NA	NA	NA	0 – inf	3396	1938	0.57	Helsby et al., 1990 [18]
200 mg SD PM[a,p]	NA	NA	NA	0 – inf	6563	3781	0.58	Helsby et al., 1990 [18]
200 mg SD EM[a,p]	209	149	0.71	NA	NA	NA	NA	Helsby et al., 1993 [19]
200 mg QD (Day 14) EM[a,p]	190	188	0.99	NA	NA	NA	NA	Helsby et al., 1993 [19]
200 mg SD PM[a]	273	187	0.68	NA	NA	NA	NA	Helsby et al., 1993 [19]
200 mg QD (Day 14) PM[a]	338	270	0.80	NA	NA	NA	NA	Helsby et al., 1993 [19]
		**Within 1.5-fold**	**100%**			**Within 1.5-fold**	**33%**	
		**Within 2-fold**	**100%**			**Within 2-fold**	**100%**	
**Pyrimethamine**								
100 mg SD[a]	771	594	0.77	0 – 336	76.2	67.8	0.89	Cavallito et al., 1978 [34]
25 mg SD[a,l]	214	145	0.68	0 – 264	19.1	17.5	0.91	Weidekamm et al., 1982 [35]
25 mg SD[a,l]	130	144	1.11	0 – inf	18.6	22.0	1.18	Edstein, 1987 [36]
25 mg (once weekly, 8th week)[a,m]	116	107	0.92	0 – inf	10.7	10.6	0.99	Edstein et al., 1990 [37]
		**Within 1.5-fold**	**100%**			**Within 1.5-fold**	**100%**	
		**Within 2-fold**	**100%**			**Within 2-fold**	**100%**	

Compound (regimen)	C_{max} (ng/mL)			AUC window (h)	AUC (ng/mL·h)			Reference
	Obs	Sim	Sim/Obs		Obs	Sim	Sim/Obs	
**Pyronaridine**								
6 mg/kg pyronaridine salt SD[a,j]	0.186	0.212	1.14	0 – inf	8.71	11.0	1.27	Wattanavijitkul, 2010 [38]
9 mg/kg pyronaridine salt SD[a,j]	0.262	0.318	1.21	0 – inf	7.67	16.5	2.15	Wattanavijitkul, 2010 [38]
12 mg/kg pyronaridine salt SD[a,j]	0.467	0.424	0.91	0 – inf	12.4	22.0	1.78	Wattanavijitkul, 2010 [38]
15 mg/kg pyronaridine salt SD[a,j]	0.792	0.530	0.67	0 – inf	20.8	27.6	1.33	Wattanavijitkul, 2010 [38]
410 free base, 3 daily doses[a,c]	0.370	0.489	1.32	48 – 960	25.9	40.6	1.57	Morris et al., 2014 [39]
		**Within 1.5-fold**	**100%**			**Within 1.5-fold**	**40%**	
		**Within 2-fold**	**100%**			**Within 2-fold**	**80%**	
**Quinine**								
600 mg SD	3100	3060	0.99	0 – inf	52000	44800	0.86	Ho et al., 1999 [40]
550 mg SD	2848	2884	1.01	0 – inf	50890	38390	0.75	Mirghani et al., 1999 [41]
600 mg SD	4600	3023	0.66	0 – inf	66000	44386	0.67	Wanwimolruk et al., 1995 [42]
		**Within 1.5-fold**	**67%**			**Within 1.5-fold**	**100%**	
		**Within 2-fold**	**100%**			**Within 2-fold**	**100%**	
**Sulfadoxine**								
500 mg SD[c]	63.2[#]	46.17[#]	0.73	0 – inf	14168[#]	10733[#]	0.76	Weidekamm et al., 1982 [35]
1500 mg SD[c]	142[#]	154[#]	1.09	0 – inf	33284[#]	32599[#]	0.98	Karunajeewa et al., 2009 [43]
500 mg weekly (20 weeks)[c]	121[#]	96.3[#]	0.79	3360 – 3528	12465[#]	11321[#]	0.91	Schwartz et al., 1987 [32]
500 mg SD[d]	77.0[#]	66.1[#]	0.86	0 – inf	26689[#]	14195[#]	0.53	Sarikabhuti et al., 1988 [44]
		**Within 1.5-fold**	**100%**			**Within 1.5-fold**	**75%**	
		**Within 2-fold**	**100%**			**Within 2-fold**	**100%**	
**Tafenoquine**								
300 mg SD	186	273	1.47	0 – 72	10611	15056	1.42	Green et al., 2014 [45]
600 mg SD	422	389	0.92	0 – 72	22986	23374	1.02	Green et al., 2014 [45]
400 mg QD (3 days)	724	686	0.95	0 – 72	41896	43990	1.05	Green et al., 2014 [45]
		**Within 1.5-fold**	**100%**			**Within 1.5-fold**	**100%**	
		**Within 2-fold**	**100%**			**Within 2-fold**	**100%**	

Compound (regimen)	C_{max} (ng/mL)			AUC window (h)	AUC (ng/mL·h)			Reference
	Obs	Sim	Sim/Obs		Obs	Sim	Sim/Obs	
**DSM265**								
400 mg SD[a]	11500	8309	0.72	0 – inf	1210000	1052927	0.87	McCarthy et al., 2017 [46]
600 mg SD[a]	15500	12464	0.80	0 – inf	2140000	1579395	0.74	McCarthy et al., 2017 [46]
800 mg SD[a]	19100	16619	0.87	0 – inf	2220000	2105845	0.95	McCarthy et al., 2017 [46]
1200 mg SD[a]	34800	24928	0.72	0 – inf	4720000	3158787	0.67	McCarthy et al., 2017 [46]
	**Within 1.5-fold**		**100%**		**Within 1.5-fold**		**100%**	
	**Within 2-fold**		**100%**		**Within 2-fold**		**100%**	
**DSM450**								
400 mg SD[a]	1070	873	0.82	0 – inf	271000	337751	1.25	McCarthy et al., 2017 [46]
600 mg SD[a]	1380	1309	0.95	0 – inf	477000	506626	1.06	McCarthy et al., 2017 [46]
800 mg SD[a]	1920	1746	0.91	0 – inf	454000	675501	1.49	McCarthy et al., 2017 [46]
1200 mg SD[a]	3070	2619	0.85	0 – inf	1170000	1013252	0.87	McCarthy et al., 2017 [46]
	**Within 1.5-fold**		**100%**		**Within 1.5-fold**		**100%**	
	**Within 2-fold**		**100%**		**Within 2-fold**		**100%**	
**MMV048**								
40 mg SD[a]	272.7	253	0.93	0 – inf	30320	55258	1.82	McCarthy et al., 2020 [47]
80 mg SD[a]	561.0	506	0.90	0 – inf	82680	110516	1.34	McCarthy et al., 2020 [47]
120 mg SD[a]	1094.0	759	0.69	0 – inf	137800	165774	1.20	McCarthy et al., 2020 [47]
	**Within 1.5-fold**		**100%**		**Within 1.5-fold**		**67%**	
	**Within 2-fold**		**100%**		**Within 2-fold**		**100%**	

[a]healthy volunteers; [b]malaria-infected individuals; [c]Caucasian; [d]South-East Asian; [e]African; [f]Chinese, [g]Thai, [h]Brazillian, [i]Vietnamese, [j]Korean, [k]Indian,

[l]Co-administered with 500 mg sulfadoxine, [m]Co-administered with 100 mg dapsone, [n]tetraphosphate salt, simulated free base dose = 739 mg, [o]tetraphosphate salt, simulated free base dose = 554 mg, [p]dose corrected to free base for simulations.

[#]µg/mL for C_{max} or µg·h/mL AUC; [+]mg/L for C_{max} or mg·h/L for AUC; [*]µM for C_{max} or µM·h AUC

SD, single dose; QD, once a day; BID, twice a day; EM, extensive metabolisers; PM, poor metabolisers; Sim, simulated; Obs, observed; Sim/Obs, the ratio of simulated to clinically observed PK parameters; inf, infinity

References

1. Scarsi KK, Fehintola FA, Ma Q, et al. Disposition of amodiaquine and desethylamodiaquine in HIV-infected Nigerian subjects on nevirapine-containing antiretroviral therapy. J Antimicrob Chemother. 2014;69:1370-1376.

2. Liu Y, Hu C, Liu G, et al. A replicate designed bioequivalence study to compare two fixed-dose combination products of artesunate and amodiaquine in healthy chinese volunteers. Antimicrob Agents Chemother. 2014;58:6009-6015.

3. Soyinka JO, Onyeji CO, Omoruyi SI, et al. Pharmacokinetic interactions between ritonavir and quinine in healthy volunteers following concurrent administration. Br J Clin Pharmacol. 2010;69:262-270.

4. Akande AA, Olugbenga SJ, Adebanjo AJ, Toyin ASa, Ogbona OC. Effects of co-trimoxazole co-administration on the pharmacokinetics of amodiaquine in healthy volunteers. International Journal of Pharmacy and Pharmaceutical Sciences. 2015;7:272-276.

5. Lefevre G, Carpenter P, Souppart C, et al. Pharmacokinetics and electrocardiographic pharmacodynamics of artemether-lumefantrine (Riamet) with concomitant administration of ketoconazole in healthy subjects. Br J Clin Pharmacol. 2002;54:485-492.

6. Lefevre G, Bhad P, Jain JP, et al. Evaluation of two novel tablet formulations of artemether-lumefantrine (Coartem) for bioequivalence in a randomized, open-label, two-period study. Malar J. 2013;12:312.

7. Lefevre G, Carpenter P, Souppart C, et al. Interaction trial between artemether-lumefantrine (Riamet) and quinine in healthy subjects. J Clin Pharmacol. 2002;42:1147-1158.

8. Huang L, Parikh S, Rosenthal PJ, et al. Concomitant efavirenz reduces pharmacokinetic exposure to the antimalarial drug artemether-lumefantrine in healthy volunteers. J Acquir Immune Defic Syndr. 2012;61:310-316.

9. Rolan PE, Mercer AJ, Weatherley BC, et al. Examination of some factors responsible for a food-induced increase in absorption of atovaquone. Br J Clin Pharmacol. 1994;37:13-20.

10. Thapar MM, Ashton M, Lindegardh N, et al. Time-dependent pharmacokinetics and drug metabolism of atovaquone plus proguanil (Malarone) when taken as chemoprophylaxis. Eur J Clin Pharmacol. 2002;58:19-27.

11. Foulds G, Shepard RM, Johnson RB. The pharmacokinetics of azithromycin in human serum and tissues. J Antimicrob Chemother. 1990;25 Suppl A:73-82.

12. Mihaly GW, Ward SA, Edwards G, et al. Pharmacokinetics of primaquine in man. I. Studies of the absolute bioavailability and effects of dose size. Br J Clin Pharmacol. 1985;19:745-750.

13. Hanboonkunupakarn B, Ashley EA, Jittamala P, et al. Open-label crossover study of primaquine and dihydroartemisinin-piperaquine pharmacokinetics in healthy adult thai subjects. Antimicrob Agents Chemother. 2014;58:7340-7346.

14. Ward SA, Mihaly GW, Edwards G, et al. Pharmacokinetics of primaquine in man. II. Comparison of acute vs chronic dosage in Thai subjects. Br J Clin Pharmacol. 1985;19:751-755.

15. Jittamala P, Pukrittayakamee S, Ashley EA, et al. Pharmacokinetic interactions between primaquine and pyronaridine-artesunate in healthy adult Thai subjects. Antimicrob Agents Chemother. 2015;59:505-513.

16. Gustafsson LL, Walker O, Alvan G, et al. Disposition of chloroquine in man after single intravenous and oral doses. Br J Clin Pharmacol. 1983;15:471-479.

17. Wattanagoon Y, Taylor RB, Moody RR, et al. Single dose pharmacokinetics of proguanil and its metabolites in healthy subjects. Br J Clin Pharmacol. 1987;24:775-780.

18. Helsby NA, Ward SA, Edwards G, Howells RE, Breckenridge AM. The pharmacokinetics and activation of proguanil in man: consequences of variability in drug metabolism. Br J Clin Pharmacol. 1990;30:593-598.

19. Helsby NA, Edwards G, Breckenridge AM, Ward SA. The multiple dose pharmacokinetics of proguanil. Br J Clin Pharmacol. 1993;35:653-656.

20. Na-Bangchang K, Krudsood S, Silachamroon U, et al. The pharmacokinetics of oral dihydroartemisinin and artesunate in healthy Thai volunteers. Southeast Asian J Trop Med Public Health. 2004;35:575-582.

21. Na-Bangchang K, Congpoung K, Ubalee R, et al. Pharmacokinetics and ex vivo anti-malarial activity of sera following a single oral dose of dihydroartemisinin in healthy Thai males. Southeast Asian J Trop Med Public Health. 1997;28:731-735.

22. Le NH, Na-Bangchang K, Le TD, Thrinh KA, Karbwang J. Phamacokinetics of a single oral dose of dihydroartemisinin in Vietnamese healthy volunteers. Southeast Asian J Trop Med Public Health. 1999;30:11-16.

23. Chinh NT, Quang NN, Thanh NX, et al. Pharmacokinetics and bioequivalence evaluation of two fixed-dose tablet formulations of dihydroartemisinin and piperaquine in Vietnamese subjects. Antimicrob Agents Chemother. 2009;53:828-831.

24. Grahnen A, Olsson B, Johansson G, Eckernas SA. Doxycycline carrageenate--an improved formulation providing more reliable absorption and plasma concentrations at high gastric pH than doxycycline monohydrate. Eur J Clin Pharmacol. 1994;46:143-146.

25. Malmborg A-S. Bioavailability of doxycycline monohydrate. Chemotherapy. 1984;30:76-80.

26. Wojcicki J, Kalinowski W, Gawronska-Szklarz B. Comparative pharmacokinetics of doxycycline and oxytetracycline in patients with hyperlipidemia. Arzneimittelforschung. 1985;35:991-993.

27. Newton PN, Chaulet JF, Brockman A, et al. Pharmacokinetics of oral doxycycline during combination treatment of severe falciparum malaria. Antimicrob Agents Chemother. 2005;49:1622-1625.

28. Binh VQ, Chinh NT, Thanh NX, et al. Sex affects the steady-state pharmacokinetics of primaquine but not doxycycline in healthy subjects. Am J Trop Med Hyg. 2009;81:747-753.

29. Schwartz DE, Eckert G, Hartmann D, et al. Single dose kinetics of mefloquine in man. Plasma levels of the unchanged drug and of one of its metabolites. Chemotherapy. 1982;28:70-84.

30. Boudreau EF, Fleckenstein L, Pang LW, et al. Mefloquine kinetics in cured and recrudescent patients with acute falciparum malaria and in healthy volunteers. Clin Pharmacol Ther. 1990;48:399-409.

31. Na-Bangchang K, Tippawangkosol P, Thanavibul A, Ubalee R, Karbwang J. Pharmacokinetic and pharmacodynamic interactions of mefloquine and dihydroartemisinin. Int J Clin Pharmacol Res. 1999;19:9-17.

32. Schwartz DE, Weidekamm E, Mimica I, Heizmann P, Portmann R. Multiple-dose pharmacokinetics of the antimalarial drug Fansimef (pyrimethamine + sulfadoxine + mefloquine) in healthy subjects. Chemotherapy. 1987;33:1-8.

33. EMA Website. Eurartesim: EPAR - Public assessment report. https://www.ema.europa.eu/en/documents/assessment-report/eurartesim-epar-public-assessment-report_en.pdf. Accessed December 8, 2022

34. Cavallito JC, Nichol CA, Brenckman WD, Jr., et al. Lipid-soluble inhibitors of dihydrofolate reductase. I. Kinetics, tissue distribution, and extent of metabolism of pyrimethamine, metoprine, and etoprine in the rat, dog, and man. Drug Metab Dispos. 1978;6:329-337.

35. Weidekamm E, Plozza-Nottebrock H, Forgo I, Dubach UC. Plasma concentrations in pyrimethamine and sulfadoxine and evaluation of pharmacokinetic data by computerized curve fitting. Bull World Health Organ. 1982;60:115-122.

36. Edstein MD. Pharmacokinetics of sulfadoxine and pyrimethamine after Fansidar administration in man. Chemotherapy. 1987;33:229-233.

37. Edstein MD, Rieckmann KH, Veenendaal JR. Multiple-dose pharmacokinetics and in vitro antimalarial activity of dapsone plus pyrimethamine (Maloprim) in man. Br J Clin Pharmacol. 1990;30:259-265.

38. Wattanavijitkul T. Population pharmacokinetics of pyronaridine in the treatment of malaria. PhD thesis, The University of Iowa, 2010.

39. Morris CA, Pokorny R, Lopez-Lazaro L, et al. Pharmacokinetic interaction between pyronaridine-artesunate and metoprolol. Antimicrob Agents Chemother. 2014;58:5900-5908.

40. Ho PC, Chalcroft SC, Coville PF, Wanwimolruk S. Grapefruit juice has no effect on quinine pharmacokinetics. Eur J Clin Pharmacol. 1999;55:393-398.

41. Mirghani RA, Hellgren U, Westerberg PA, et al. The roles of cytochrome P450 3A4 and 1A2 in the 3-hydroxylation of quinine in vivo. Clin Pharmacol Ther. 1999;66:454-460.

42. Wanwimolruk S, Kang W, Coville PF, Viriyayudhakorn S, Thitiarchakul S. Marked enhancement by rifampicin and lack of effect of isoniazid on the elimination of quinine in man. Br J Clin Pharmacol. 1995;40:87-91.

43. Karunajeewa HA, Salman S, Mueller I, et al. Pharmacokinetic properties of sulfadoxine-pyrimethamine in pregnant women. Antimicrob Agents Chemother. 2009;53:4368-4376.

44. Sarikabhuti B, Keschamrus N, Noeypatimanond S, et al. Plasma concentrations of sulfadoxine in healthy and malaria infected Thai subjects. Acta Trop. 1988;45:217-224.

45. Green JA, Patel AK, Patel BR, et al. Tafenoquine at therapeutic concentrations does not prolong Fridericia-corrected QT interval in healthy subjects. J Clin Pharmacol. 2014;54:995-1005.

46. McCarthy JS, Lotharius J, Ruckle T, et al. Safety, tolerability, pharmacokinetics, and activity of the novel long-acting antimalarial DSM265: a two-part first-in-human phase 1a/1b randomised study. Lancet Infect Dis. 2017;17:626-635.

47. McCarthy JS, Donini C, Chalon S, et al. A Phase 1, Placebo-controlled, Randomized, Single Ascending Dose Study and a Volunteer Infection Study to Characterize the Safety, Pharmacokinetics, and Antimalarial Activity of the Plasmodium Phosphatidylinositol 4-Kinase Inhibitor MMV390048. Clin Infect Dis. 2020;71:e657-e664.
