# Supplementary material for: Development and application of a PBPK modeling strategy to support antimalarial drug development
Source: CPT Pharmacometrics Syst Pharmacol. 2023 Aug 16;12(9):1335–46. doi: 10.1002/psp4.13013 (PMC10508484; doi:10.1002/psp4.13013)
Supplement: Supplementary file 3 — Table S3 [file PSP4-12-1335-s006.pdf]

Table S3. Comparison of simulated C_{max} and AUC ratios to those observed clinically in drug-drug interaction studies.

Victim (regimen)	Perpetrator (regimen)	C_{max} ratios			AUC ratios			Reference
		Obs	Sim	Sim/Obs	Obs	Sim	Sim/Obs	
**Amodiaquine**								
600 mg SD (Day 6)[e,i]	**Trimethoprim** 160 mg QD (7 Days)	1.62	1.67	1.03	1.56	1.54	0.99	Akande et al., 2015 [1]
			**Within 1.5-fold**	**100%**		**Within 1.5-fold**	**100%**	
			**Within 2-fold**	**100%**		**Within 2-fold**	**100%**	
**Artemether**								
80 mg SD (Day 1)[a,c]	**Ketoconazole** 400 mg SD (Day 1), 200 mg QD (Days 2 – 5)	2.24	3.23	1.44	2.51	2.89	1.15	Lefevre et al., 2002 [2]
80 mg BID (Day 22 - 24)[e,h]	**Rifampicin** 600 mg QD (Days 1 – 24)	0.18	0.11	0.59	0.11	0.11	1.00	Lamorde et al., 2013 [3]
80 mg BID (Days 13 - 15)[a,c]	**Efavirenz** 600 mg QD (Days 1 – 16)	0.79	0.54	0.69	0.49	0.53	1.08	Huang et al., 2012 [4]
			**Within 1.5-fold**	**67%**		**Within 1.5-fold**	**100%**	
			**Within 2-fold**	**100%**		**Within 2-fold**	**100%**	
**Atovaquone**	No DDI studies available							
**Midazolam**	**Azithromycin**							
15 mg SD (Day 5)	500 mg QD (Day 1), 250 mg (Days 2-5)	0.66	1.04	1.58	0.87	1.06	1.22	Backman et al., 1995 [5]
15 mg SD (Day 3)	500 mg QD (3 Days)	1.52	1.06	0.70	1.19	1.08	0.91	Yeates et al., 1996 [6]
15 mg SD (Day 3)	500 mg QD (3 Days)	1.29	1.04	0.81	1.27	1.06	0.83	Zimmermann et al., 1996 [7]
			**Within 1.5-fold**	**67%**		**Within 1.5-fold**	**100%**	
			**Within 2-fold**	**100%**		**Within 2-fold**	**100%**	
**Chloroquine**	No DDI studies available							
**DEAQ** 600 mg SD (Day 6)[e,i]	**Trimethoprim** 160 mg QD (7 Days)	0.75	0.75	1.00	0.874	0.779	0.89	Akande et al., 2015 [1]
			**Within 1.5-fold**	**100%**		**Within 1.5-fold**	**100%**	
			**Within 2-fold**	**100%**		**Within 2-fold**	**100%**	
**DHA**	No DDI studies available							
**DHA** (administered as artesunate)	No DDI studies available							

Victim (regimen)	Perpetrator (regimen)	C_{max} ratios			AUC ratios			Reference
		Obs	Sim	Sim/Obs	Obs	Sim	Sim/Obs	
**Doxycycline**	No DDI studies available							
**Lumefantrine**								
480 mg SD (Day 1)[a,c,i]	**Ketoconazole** 400 mg SD (Day 1), 200 mg QD (Days 2 – 5)	1.26	1.33	1.06	1.65	1.58	0.96	Lefevre et al., 2002 [2]
480 mg BID (Day 22 - 24)[e,h]	**Rifampicin** 600 mg QD (Days 1 – 46)	0.16[#]	0.036[#]	0.23[#]	0.32	0.12	0.38	Lamorde et al., 2013 [3]
480 mg BID (Days 13 - 15)[a,c]	**Efavirenz** 600 mg QD (Days 1 – 26)	1.04	0.63	0.61	0.79	0.39	0.49	Huang et al., 2012 [4]
480 mg BID (Days 13 - 15)[e,h]	**Efavirenz** 600 mg QD (Days 1 – 26)	0.72	0.66	0.92	0.44	0.43	0.97	Byakika-Kibwika et al., 2012 [8]
480 mg BID (Days 13 - 15)[a,c]	**Ritonavir** 100 mg BID (Days 1 – 26)	1.39	1.50	1.08	2.25	2.21	0.98	German et al., 2009 [9]
		**Within 1.5-fold**		**60%**	**Within 1.5-fold**		**60%**	
		**Within 2-fold**		**80%**	**Within 2-fold**		**60%**	
**Mefloquine**								
500 mg SD (Day 7)[a,f]	**Rifampicin** 600 mg QD (7 Days), 600 mg 2x/week (Days 8 – 56)	0.81	0.54	0.66	0.32	0.21	0.64	Ridtitid et al., 2000 [10]
500 mg SD (Day 5)[a,f]	**Ketoconazole** 400 mg QD (10 Days)	1.64	1.09	0.66	1.79	2.29	1.28	Ridtitid et al., 2005 [11]
		**Within 1.5-fold**		**0%**	**Within 1.5-fold**		**50%**	
		**Within 2-fold**		**100%**	**Within 2-fold**		**100%**	
**Primaquine**								
**Piperaquine** 960 mg SD	**Clarithromycin** 500 mg SD	≤ 2-fold	1.08	--	≤ 2-fold	1.17	--	Eurartesim - EMA Website, [12]
**Midazolam** 7.5 mg QD[a]	**Piperaquine** 960 mg QD	≤ 2-fold	1.46	--	≤ 2-fold	1.98	--	Eurartesim - EMA Website, [12]
**Proguanil**	**Fluvoxamine**							
200 mg SD (Day 6)[a, i]	100 mg QD (8 Days)	NA	NA	NA	0.99	1.01	1.01	Jeppesen et al., 1997 [13]
200 mg SD (Day 6)[a,i]	100 mg QD (8 Days)	NA	NA	NA	1.86	1.48	0.80	Jeppesen et al., 1997 [13]
		**Within 1.5-fold**		**100%**	**Within 1.5-fold**		**100%**	
		**Within 2-fold**		**100%**	**Within 2-fold**		**100%**	
**Pyrimethamine**	No DDI studies available							
**Pyronaridine**	No DDI studies available							

Victim (regimen)	Perpetrator (regimen)	C_{max} ratios			AUC ratios			Reference
		Obs	Sim	Sim/Obs	Obs	Sim	Sim/Obs	
**Quinine**								
500 mg SD (Day 1)[a]	**Ketoconazole** 200 mg BID (4 Days)	1.08	1.14	1.06	1.45	1.76	1.21	Mirghani et al., 1999 [14]
500 mg SD (Day 2)[a]	**Erythromycin** 600 mg QD (7 Days)	1.20	1.12	0.93	1.53	1.86	1.22	Orlando et al., 2009 [15]
600 mg SD (Day 14)[a]	**Rifampicin** 600 mg QD (15 Days)	0.48	0.50	1.04	0.17	0.19	1.11	Wanwimolruk et al., 1995 [16]
		**Within 1.5-fold**		**100%**	**Within 1.5-fold**		**100%**	
		**Within 2-fold**		**100%**	**Within 2-fold**		**100%**	
**Sulfadoxine**	No DDI studies available							
**Tafenoquine**	No DDI studies available							
**DSM265**	No DDI studies available							
**DSM450**	No DDI studies available							
**MMV048**	No DDI studies available							

[a]healthy volunteers; [b]malaria-infected individuals; [c]Caucasian; [d]South-East Asian; [e]African; [f]Thai, [g]Vietnamese, [h]HIV-infected individuals; [i]used in model development; [#]Day 7 concentrations

SD, single dose; QD, once a day; BID, twice a day; DDI, drug-drug interactions; Sim, simulated; Obs, observed; Sim/Obs, the ratio of simulated to clinically observed PK ratios.

References

1. Akande AA, Olugbenga SJ, Adebanjo AJ, Toyin ASa, Ogbona OC. Effects of co-trimoxazole co-administration on the pharmacokinetics of amodiaquine in healthy volunteers. *International Journal of Pharmacy and Pharmaceutical Sciences.* 2015;7:272-276.

2. Lefevre G, Carpenter P, Souppart C, et al. Pharmacokinetics and electrocardiographic pharmacodynamics of artemether-lumefantrine (Riamet) with concomitant administration of ketoconazole in healthy subjects. *Br J Clin Pharmacol.* 2002;54:485-492.

3. Lamorde M, Byakika-Kibwika P, Mayito J, et al. Lower artemether, dihydroartemisinin and lumefantrine concentrations during rifampicin-based tuberculosis treatment. *AIDS.* 2013;27:961-965.

4. Huang L, Parikh S, Rosenthal PJ, et al. Concomitant efavirenz reduces pharmacokinetic exposure to the antimalarial drug artemether-lumefantrine in healthy volunteers. *J Acquir Immune Defic Syndr.* 2012;61:310-316.

5. Backman JT, Olkkola KT, Neuvonen PJ. Azithromycin does not increase plasma concentrations of oral midazolam. *Int J Clin Pharmacol Ther.* 1995;33:356-359.

6. Yeates RA, Laufen H, Zimmermann T. Interaction between midazolam and clarithromycin: comparison with azithromycin. *Int J Clin Pharmacol Ther.* 1996;34:400-405.

7. Zimmermann T, Yeates RA, Laufen H, et al. Influence of the antibiotics erythromycin and azithromycin on the pharmacokinetics and pharmacodynamics of midazolam. *Arzneimittelforschung.* 1996;46:213-217.

8. Byakika-Kibwika P, Lamorde M, Mayito J, et al. Significant pharmacokinetic interactions between artemether/lumefantrine and efavirenz or nevirapine in HIV-infected Ugandan adults. *J Antimicrob Chemother.* 2012;67:2213-2221.

9. German P, Parikh S, Lawrence J, et al. Lopinavir/ritonavir affects pharmacokinetic exposure of artemether/lumefantrine in HIV-uninfected healthy volunteers. *J Acquir Immune Defic Syndr.* 2009;51:424-429.

10. Ridtitid W, Wongnawa M, Mahatthanatrakul W, Chaipol P, Sunbhanich M. Effect of rifampin on plasma concentrations of mefloquine in healthy volunteers. *J Pharm Pharmacol.* 2000;52:1265-1269.

11. Ridtitid W, Wongnawa M, Mahatthanatrakul W, Raungsri N, Sunbhanich M. Ketoconazole increases plasma concentrations of antimalarial mefloquine in healthy human volunteers. *J Clin Pharm Ther.* 2005;30:285-290.

12. EMA Website. Eurartesim: EPAR - Public assessment report. https://www.ema.europa.eu/en/documents/assessment-report/eurartesim-epar-public-assessment-report_en.pdf. Accessed December 8, 2022

13. Jeppesen U, Rasmussen BB, Brosen K. Fluvoxamine inhibits the CYP2C19-catalyzed bioactivation of chloroguanide. *Clin Pharmacol Ther.* 1997;62:279-286.

14. Mirghani RA, Hellgren U, Westerberg PA, et al. The roles of cytochrome P450 3A4 and 1A2 in the 3-hydroxylation of quinine in vivo. *Clin Pharmacol Ther.* 1999;66:454-460.

15. Orlando R, De Martin S, Pegoraro P, Quintieri L, Palatini P. Irreversible CYP3A inhibition accompanied by plasma protein-binding displacement: a comparative analysis in subjects with normal and impaired liver function. *Clin Pharmacol Ther.* 2009;85:319-326.

16. Wanwimolruk S, Kang W, Coville PF, Viriyayudhakorn S, Thitiarchakul S. Marked enhancement by rifampicin and lack of effect of isoniazid on the elimination of quinine in man. *Br J Clin Pharmacol.* 1995;40:87-91.
