# Supplementary material for: Development and application of a PBPK modeling strategy to support antimalarial drug development
Source: CPT Pharmacometrics Syst Pharmacol. 2023 Aug 16;12(9):1335–46. doi: 10.1002/psp4.13013 (PMC10508484; doi:10.1002/psp4.13013)
Supplement: Supplementary file 4 — Appendix S1 [file PSP4-12-1335-s005.docx]

**Development and Application of a PBPK modelling strategy to support antimalarial drug development**

Nada Abla, Eleanor Howgate, Karen Rowland-Yeo, Maurice Dickins, Mackenzie Kolev, Kuan-Fu Chen, Savannah McFeely, Jennifer Bonner, Laura Santos, Nathalie Gobeau, Howard Burt, Zoe Barter, Hannah M. Jones, David Wesche, Susan A. Charman, Jörg J. Möhrle, Jeremy N. Burrows and Lisa M. Almond

**Supplemental Supporting Information**

The PBPK models for all 20 antimalarial drugs (including two development antimalarial compounds MMV048 and DSM265) and 4 associated metabolites were developed in collaboration among Certara, Medicines for Malaria Venture (MMV) and Monash University and are freely available on the Simcyp Global Health Model Repository following a pre-registration step (https://members.certara.co.uk/Simcyp/GlobalHealthRepository). The repository contains slide decks summarising model development and verification for each compound. In this supplementary material we have provided the model inputs for all the drugs (Table S1) and have provided simulated concentration-time profiles that demonstrate the performance of the PBPK models for the drugs used in the 3 case studies (artemether, lumefantrine, pyronaridine, DSM265, DSM450 and MMV048) and a comparison of simulated and observed PK parameters (Table S2) and interaction ratios (Table S3), where clinical data are available. .

**Supplemental Methods**

Data searching and feasibility analysis

In order to develop PBPK models, various physicochemical, *in vitro* DMPK (e.g. plasma protein binding, metabolism) and clinical PK data are required. A feasibility analysis of PBPK model development for each of the prioritized compounds was performed to:

1. Identify data and knowledge gaps that would hinder the development of PBPK models
2. Consolidate the main purpose of the PBPK modelling for each case
3. Gain understanding of the main clinical issues for each compound

The analyses were based on information from multiple sources, predominantly the literature and public databases for clinical PK of the legacy compounds.

*In vitro data used for model development*

The majority of *in vitro* DMPK and physicochemical data were generated at Monash University^1^. *In vitro* cytochrome P450 (CYP) and UDP glucuronosyl transferases (UGT) reaction phenotyping experiments, as well as UGT and P-glycoprotein (P-gp) inhibition assays (conducted only on selected compounds), were carried out at a contract research organisation.

In nearly all cases the fraction of systemic elimination metabolised by a given enzyme (fm) determined *in vitro* was unavailable in the literature. As a critical parameter for DDI prediction, these data were assessed either using human liver microsomes (HLM) with specific inhibitors (for artemether) or, for antimalarials exhibiting a lower turnover, from substrate depletion assays in a panel of recombinant CYP (for mefloquine, (DEAQ, pyronaridine, lumefantrine, MMV048 and primaquine) or UGT (MMV048 and DHA) assays. Where available, clinical data in the literature were used to verify *in vitro* fm.

Specifically, physicochemical and *in vitro* DMPK data were generated at CDCO (University of Monash)^1^: log D at pH 7.4, pKa, apparent permeability (Papp) in Caco-2 cells, fraction unbound in plasma (fu), blood to plasma ratio (B:P), intrinsic clearance (CL_int_) in human liver microsomes (HLM), fraction unbound in human liver microsomes (fu_mic_), aqueous solubility at pH 7.4, solubility in Fasted State Simulated Intestinal Fluid (FaSSIF), Fed State Simulated Intestinal Fluid (FeSSIF) and Fasted State Simulated Gastric Fluid (FaSSGF), CYP inhibition (direct and time-dependent inhibition). Bactosomes^TM^ supplied by Cypex Ltd (CYP1A2, CYP2B6, CYP2C8, CYP2C9, CYP2C19, CYP2D6, CYP3A4 and CYP3A5) were used for CYP reaction phenotyping of lumefantrine, primaquine, pyronaridine, mefloquine, DEAQ and MMV048, whereas HLM with specific CYP inhibitors were used for artemether. For UGT reaction phenotyping, DHA and MMV048 were incubated with Supersomes supplied by BD Biosciences (UGT1A1, UGT1A3, UGT1A6, UGT1A9, UGT2B7 and UGT2B15). UGT1A1 inhibition data for DSM265 and DSM450 were generated as part of the drug packages.

Approach for Compound Model Development and Verification

Initial models were built based on available physicochemical and *in vitro* data to give a ‘bottom-up’ prediction. Areas of uncertainty were highlighted, and the potential impact of uncertainty associated with such parameters was investigated using sensitivity analysis. Available clinical PK data were then used to evaluate and, if required, refine the initial ‘bottom-up’ model. Care was taken to split data into training and test sets wherever possible. The model verification strategy differed across compounds based on availability of different types of clinical data. For example, where possible the fraction metabolised by a specific enzyme was verified using either a clinical DDI study with a strong inhibitor or using PK in individuals with pharmacogenetic differences. Clinical studies used for drug model development and verification are reported in Table S2 (PK) and Table S3 (DDIs). An overview of model development and verification for each drug model is provided (Supplemental Information, Model Overviews) and further details for those used in the cases studies presented in this manuscript are provided below (Supplemental Results).

As indicated in Table S1, in cases where metabolic intrinsic clearance (CL_int_) generated *in vitro* did not extrapolate to recover the clinically observed clearance, unbound hepatic intrinsic clearance (CLu_intH_) was back calculated from *in vivo* clearance using a retrograde approach. This approach was often coupled with estimates of fm_CYP_ derived *in vitro* or *in vivo* to assign CLu_intH_ to different enzymes. Depending on data availability, CLu_intH_ can be back calculated from either a systemic CL (Equation (1) and Equation (2)) or an oral CL (CL/F) as described in Equation (3)

$$CLmet=\left( \frac{{CL}_{iv}}{B:P} \right)-\left( \frac{{CL}_{R}}{B:P} \right)$$

Equation (1)

$${CLu}_{int,H}=\frac{(Q_{H}\times{CL}_{met})}{\left( {fu}_{b}\times(Q_{H}-{CL}_{met}) \right)}$$

Equation (2)

$${CLu}_{intH}=\frac{\frac{CL/F}{B:P}faF_{G}-\frac{{CL}_{R}}{B:P}}{{fu}_{b}\left( 1+\frac{\frac{{CL}_{R}}{B:P}}{Q_{H}} \right)}$$

Equation (3)

*Where CL_met_ is metabolic clearance; CL_iv_ is systemic clearance; CL_R_ is the renal clearance; B:P is the blood to plasma concentration ratio of drug; fu_b_ is fraction of unbound drug in blood (calculated from fu_p_/B:P); Q_H_ is the blood flow in the hepatic vein (90 L/h); CL/F is the observed oral clearance; F_G_ is fraction escaping first pass metabolism in the gut; fa is fraction absorbed.*

Acceptance criteria

When comparing simulated *versus* observed exposure of drugs, being within 2fold of observed data is considered to be‑ “a primary metric for assessment of model fidelity” ^2^. However, it should be noted that when clinical DDI data are available to optimise the PBPK model, this metric is often reduced to within 1.25‑ or within 1.5-fold. The challenge with this work was the varying abundance and heterogenous nature of published literature data where the full information, such as specific study design or subject data, are often missing. Furthermore, when relying on literature, clinical data for overlaying onto simulated data is often extracted from publication graphics as raw data is inaccessible. In light of these challenges, simulations within 2-fold of observed were deemed acceptable and verified for use, although specific parameters for each compound were considered on a case-by-case basis (See Supplemental Information, Model Overviews).

Simulation Design

All simulations, including the ones performed for the applications, were run using Version 19 of the Simcyp population-based PBPK simulator (Simcyp (Certara), Sheffield, UK) and unless stated otherwise, input parameters for all drugs are as specified (Supplementary Information Table S1). All simulations were run using ten virtual trials that were matched as closely as possible to the clinical study volunteers in terms of the number of subjects, their age, the proportion of males/females and according to the same study design.

Virtual Populations

The available clinical PK data for performance verification of simulations was largely in healthy volunteers of either Caucasian or South-East Asian (Thai or Vietnamese) descent. For Caucasian subjects the default Sim-Healthy Volunteers virtual population was used without modification. For East Asian subjects, the body weight of virtual subjects was reduced to better reflect the observed characteristics and Japanese genotype/phenotype frequencies of drug metabolising enzymes were taken as a surrogate. The case study simulations presented in this paper used the Sim-Healthy Volunteers virtual population.

**Supplemental Results**

A comparison of predicted and observed PK parameters and interaction ratios are provided for all drug models. More detailed information is provided for the 4 drug models used in the case studies presented in this paper (below).

*Independent Verification of the predicted concentration-time profiles of pyronaridine*

Ten virtual trials of 7 male and female subjects (41% female) aged 19 – 40 years who received a single oral dose of pyronaridine tetraphosphate A) *3.42 mg/kg free base; B) 5.12 mg/kg free base; C) 6.83 mg/kg free base and D) 8.54 mg/kg free base* were generated and the predicted and observed ^3^ pyronaridine blood exposures were compared. Simulated blood concentration time profiles were in good agreement with observed (Figure S1 A-D). There was a tendency to overpredict the mean AUC_0-inf_, although 3 out of 4 comparisons were within 2-fold of observed. It is also worth noting that the observed exposure at 9mg/kg dose was lower than at 6 mg/kg. The model recovered the observed data at the 6 mg/kg dose but then over predicted that at the higher dose. One challenge in the verification of the model is the diverse ethnicities of subjects in reported clinical data and how best to reflect this in simulations. In the absence of virtual Korean populations within the Simulator, the Caucasian population was modified in terms of bodyweight. In the absence of supporting information no changes to enzyme abundance (pmol/mg) were made to the population, although changes to liver weight (as a function of body weight) and hence total CYP abundance were propagated into the model. With these complexities in mind and seeing the performance of the simulated concentration-time profiles, the model was deemed verified.

*Independent Verification of the predicted concentration-time profiles of artemether*

Ten virtual trials of 16 Caucasian male and female subjects aged 19 – 49 years who received a single oral dose of 80 mg artemether were generated; predicted and observed ^4^ artemether exposures were compared. Predicted mean AUC_0-inf_ was in excellent agreement (1.04-fold) and C_max_ was in reasonable agreement (within 1.3-fold) with the clinically observed mean values. Overall, the predicted concentration-time profile was in good agreement with the observed data (Figure S2, Part A).

Ten virtual trials of 14 Caucasian male subjects aged 19 – 50 years who received six doses of 80 mg artemether by mouth were generated; predicted and observed ^5^ artemether exposures after the final dose were compared. Predicted mean C_max_ was in in good agreement with the clinically observed mean value (within 1.25-fold). The model over-predicted mean artemether AUC_62-72h,_ just outside of model acceptance criteria at 2.02-fold of observed (Figure S2, Part B).

Ten virtual trials of 12 healthy male (n=10) and female (n=2) subjects aged 24 – 53 years who received six doses of 80 mg artemether were generated and the predicted and observed ^6^ artemether exposures after the final dose were compared. Predicted mean C_max_ was in good agreement with the clinically observed mean value (within 1.25-fold), AUC_60-324h_ was over-predicted by 2.6-fold; (Figure S2, Part C).

*Independent Verification of fmCYP3A4 and CYP2B6 for artemether - simulated DDIs with CYP inhibitors and inducers*

For verification of fm_CYP_, the impact of ketoconazole (a strong CYP3A4 inhibitor), rifampicin (a strong CYP3A4 and moderate CYP2B6 inducer) and efavirenz (a moderate CYP3A4 and CYP2B6 inducer) were simulated.

Ten virtual trials of 16 Caucasian male and female subjects aged 19 – 49 years who received a single oral dose of 80 mg artemether co-administered with ketoconazole (400 mg on day 1; 200 mg QD days 2-5) were simulated ^4^. The predicted mean ratios of artemether in the absence and presence of ketoconazole were in good agreement (within 1.25-fold of observed) for AUC and reasonable agreement (within 1.5-fold of observed) for C_max_ (Table S3).

Twenty virtual trials of 5 male (n=1) and female (n=4) subjects who received six doses of 80 mg artemether by mouth in the absence and presence of rifampicin (600 mg QD for 3 weeks prior to and throughout artemether dosing) were generated. The predicted and observed ^7^ C_max_ and AUC ratios in the absence and presence of rifampicin for the final dose were in excellent agreement (within 1.05-fold of observed) for AUC and acceptable agreement (within 1.65-fold of observed) for C_max_ (Table S3).

The six doses of artemether co-administered with efavirenz (600 mg QD for 10 days prior to and throughout administration and measurement of artemether PK as described in Huang et al., 2012 ^6^) was also simulated. The predicted and observed C_max_ and AUC ratios in the absence and presence of efavirenz for the final dose were in excellent agreement (within 1.1-fold of observed) for AUC and reasonable agreement (within 1.5-fold of observed) for C_max_. This was also true for simulations of efavirenz in the absence and presence of artemether (within 0.94-fold of observed; Table S3).

As the DDIs with all 3 inhibitors/inducers were well predicted, the fm_CYP_ values were considered verified. There was a tendency towards over-prediction of artemether exposure upon multiple dosing, which could indicate a greater extent of induction is required. However, any increase in induction potency resulted in under-prediction of single dose exposure, which is of greater importance for the therapeutic effect of artemether. In addition, the CYP2B6 induction parameters used resulted in a good prediction of the impact of artemether on efavirenz PK. The observed ^6^ efavirenz C_max_ and AUC ratios in the absence and presence of artemether were 0.91 and 0.83, respectively. Corresponding simulated ratios were 0.85 and 0.79, which were in excellent agreement (within 1.1-fold of observed).

Although simulations show some over prediction of multiple dose exposure, prediction of AUC and C_max_ values for the first dose were within 1.5-fold fold of those observed. The exposure of the shorter acting artemisinin in fixed dose combination can be considered more important. Furthermore, the predicted magnitude of DDIs were in good agreement with those clinically observed. Overall, the artemether model was, therefore, deemed acceptable and verified for its intended use.

*Independent Verification of Lumefantrine plasma concentration-time profiles*

Ten virtual trials of 16 Caucasian male and female subjects aged 19 – 49 years who received a single oral dose of 480 mg lumefantrine were generated; predicted and observed ^4^ lumefantrine exposures were compared. Mean C_max_ and AUC_0-inf_ following single dose administration were under-predicted by the model; predicted C_max_ was approximately 62% of the observed value and predicted AUC_0-inf_ was approximately 66% of the observed value (Figure S3, Part A).

Ten virtual trials of 14 Caucasian male subjects aged 19 – 50 years who received six doses of 480 mg lumefantrine by mouth were generated; predicted and observed ^5^ lumefantrine exposures after the final dose were compared. Predicted mean C_max_ was in excellent agreement (1.01-fold) and AUC_62-480h_ was in good agreement (within 1.25-fold) with the clinically observed mean values. Overall, the predicted concentration-time profile was in good agreement with the observed data (Figure S3, Part B).

Ten virtual trials of 12 healthy male (n=10) and female (n=2) subjects aged 24 – 53 years who received six doses of 480 mg lumefantrine by mouth were generated and the predicted and observed ^6^ lumefantrine exposures after the final dose were compared. Predicted mean C_max_ and AUC_60-324h_ were in good agreement with the clinically observed mean values (within 1.25-fold); the predicted concentration-time profile was in good agreement with the observed data (Figure S3, Part C).

The concentration on Day 7 following repeat administration of lumefantrine is more critical (linked to cure rates) than Day 1 and hence the model performance was deemed acceptable.

*Independent Verification of the fmCYP3A4 of lumefantrine - simulated DDIs with CYP3A4 inhibitors and inducers*

A single dose of lumefantrine co-administered with ketoconazole (400 mg on day 1; 200 mg QD days 2-5 ^4^) was simulated. The predicted mean C_max_ and AUC ratios of lumefantrine in the absence and presence of ketoconazole were within 10% of the observed (Table S3).

Twenty virtual trials of 5 male (n=1) and female (n=4) subjects who received six doses of 480 mg lumefantrine by mouth in the absence and presence of rifampicin (600 mg QD for 3 weeks prior to and throughout lumefantrine dosing) were generated. The predicted and observed ^7^ ratio in the absence and presence of rifampicin for “Day 8” (7.3 days post first dose) concentrations and AUC for the final dose were compared. The extent of induction by rifampicin on lumefantrine AUC was over-predicted (Table S3).

In addition, the six doses of lumefantrine co-administered with efavirenz (600 mg QD for 10 days prior to and throughout administration and measurement of lumefantrine PK as reported by Huang et al., 2012 ^6^) was simulated. The extent of induction by efavirenz was over-predicted by ~2-fold (Table S4).

As the DDIs with efavirenz and rifampicin were over-predicted, further studies with these and other CYP3A4 inhibitors/inducers were assessed. There were no studies with rifampicin in healthy volunteers; the DDI reported by Lamorde et al., 2013 ^7^ was already in Ugandan HIV positive (antiretroviral naïve) patients with TB. A further study with efavirenz ^8^ was available in Ugandan HIV positive (antiretroviral naïve) patients, where the extent of induction was greater with lumefantrine AUC reduced by 56%, which agrees well with the model predictions (within 1.25-fold). Finally, a study with lopinavir/ritonavir ^9^ in healthy (US) volunteers, provided C_max_ and AUC GMR values of 1.39 and 2.25, respectively. The predicted impact of ritonavir (100 mg BID for 10 days prior to and throughout administration and measurement of lumefantrine PK) on lumefantrine C_max_ and AUC was GMRs of 1.50 (within 10% of observed) and 2.21 (within 2% of observed), respectively. As the effect of CYP3A4 inhibition was independently verified and there appeared to be variability in the extent of induction on lumefantrine PK, the fm_CYP3A4_ of 40% was considered verified.

*Independent Verification of the predicted concentration-time profiles of DSM265 and DSM450*

Ten virtual trials of 10 Caucasian male and female subjects aged 18-65 years who received a single oral dose of 400 mg DSM265 were generated; predicted and observed^10^ DSM265 and DSM450 exposures were compared. Mean C_max_ and AUC_0-inf_ following single dose administration were in reasonable agreement with the model; for DSM265, predicted C_max_ was approximately 72% of the observed value and predicted AUC_0-inf_ was approximately 87% of the observed value, while for DSM450 predicted C_max_ was approximately 82% of the observed value and predicted AUC_0-inf_ was approximately 125% of the observed value (Figure S4, Parts A and B, and Table S2).

Ten virtual trials of 10 Caucasian male and female subjects aged 18-65 years who received a single oral dose of 600 mg DSM265 were generated; predicted and observed^10^ DSM265 and DSM450 exposures were compared. Mean C_max_ and AUC_0-inf_ following single dose administration were in reasonable agreement with the model; for DSM265, predicted C_max_ was approximately 80% of the observed value and predicted AUC_0-inf_ was approximately 74% of the observed value, while for DSM450 predicted C_max_ was approximately 95% of the observed value and predicted AUC_0-inf_ was approximately 106% of the observed value (Figure S4, Parts C and D, and Table S2).

Ten virtual trials of 10 Caucasian male and female subjects aged 18-65 years who received a single oral dose of 800 mg DSM265 were generated; predicted and observed ^10^ DSM265 and DSM450 exposures were compared. Mean C_max_ and AUC_0-inf_ following single dose administration were in reasonable agreement with the model; for DSM265, predicted C_max_ was approximately 87% of the observed value and predicted AUC_0-inf_ was approximately 95% of the observed value, while for DSM450 predicted C_max_ was approximately 91% of the observed value and predicted AUC_0-inf_ was approximately 149% of the observed value (Table S2).

Ten virtual trials of 10 Caucasian male and female subjects aged 18-65 years who received a single oral dose of 1200 mg DSM265 were generated; predicted and observed ^10^ DSM265 and DSM450 exposures were compared. Mean C_max_ and AUC_0-inf_ following single dose administration were in reasonable agreement with the model; for DSM265, predicted C_max_ was approximately 72% of the observed value and predicted AUC_0-inf_ was approximately 67% of the observed value, while for DSM450 predicted C_max_ was approximately 85% of the observed value and predicted AUC_0-inf_ was approximately 87% of the observed value (Table S2).

It should be noted that dose specific fa should be used based on those derived from ascending dose studies. Simulations were verified for doses of 400 mg and above using fa = 0.7, while for doses between 25 and 250 mg fa=1 should be used.

*Independent Verification of the predicted concentration-time profiles of MMV048*

Ten virtual trials of 10 Caucasian male and female subjects aged 18-65 years who received a single oral dose of 40 mg MMV048 were generated; predicted and observed ^11^ MMV048 exposures were compared. Mean C_max_ and AUC_0-inf_ following single dose administration were in reasonable agreement with the model; predicted C_max_ was approximately 93% of the observed value and predicted AUC_0-inf_ was approximately 182% of the observed value (Figure S5-A and Table S2).

Ten virtual trials of 10 Caucasian male and female subjects aged 18-65 years who received a single oral dose of 80 mg MMV048 were generated; predicted and observed ^11^ MMV048 exposures were compared. Mean C_max_ and AUC_0-inf_ following single dose administration were in reasonable agreement with the model; predicted C_max_ was approximately 90% of the observed value and predicted AUC_0-inf_ was approximately 134% of the observed value (Figure S5-B and Table S2).

Ten virtual trials of 10 Caucasian male and female subjects aged 18-65 years who received a single oral dose of 120 mg MMV048 were generated; predicted and observed ^11^ MMV048 exposures were compared. Mean C_max_ and AUC_0-inf_ following single dose administration were in reasonable agreement with the model; predicted C_max_ was approximately 69% of the observed value and predicted AUC_0-inf_ was approximately 120% of the observed value (Figure S5-C and Table S2).

**References**

1. Charman SA, Andreu A, Barker H, et al. An in vitro toolbox to accelerate anti-malarial drug discovery and development. *Malar J.* 2020;19:1.

2. Wagner C, Pan Y, Hsu V, et al. Predicting the effect of cytochrome P450 inhibitors on substrate drugs: analysis of physiologically based pharmacokinetic modeling submissions to the US Food and Drug Administration. *Clin Pharmacokinet.* 2015;54:117-127.

3. Wattanavijitkul T. Population pharmacokinetics of pyronaridine in the treatment of malaria. PhD thesis, The University of Iowa, 2010.

4. Lefevre G, Carpenter P, Souppart C, et al. Pharmacokinetics and electrocardiographic pharmacodynamics of artemether-lumefantrine (Riamet) with concomitant administration of ketoconazole in healthy subjects. *Br J Clin Pharmacol.* 2002;54:485-492.

5. Lefevre G, Carpenter P, Souppart C, et al. Interaction trial between artemether-lumefantrine (Riamet) and quinine in healthy subjects. *J Clin Pharmacol.* 2002;42:1147-1158.

6. Huang L, Parikh S, Rosenthal PJ, et al. Concomitant efavirenz reduces pharmacokinetic exposure to the antimalarial drug artemether-lumefantrine in healthy volunteers. *J Acquir Immune Defic Syndr.* 2012;61:310-316.

7. Lamorde M, Byakika-Kibwika P, Mayito J, et al. Lower artemether, dihydroartemisinin and lumefantrine concentrations during rifampicin-based tuberculosis treatment. *AIDS.* 2013;27:961-965.

8. Byakika-Kibwika P, Lamorde M, Mayito J, et al. Significant pharmacokinetic interactions between artemether/lumefantrine and efavirenz or nevirapine in HIV-infected Ugandan adults. *J Antimicrob Chemother.* 2012;67:2213-2221.

9. German P, Parikh S, Lawrence J, et al. Lopinavir/ritonavir affects pharmacokinetic exposure of artemether/lumefantrine in HIV-uninfected healthy volunteers. *J Acquir Immune Defic Syndr.* 2009;51:424-429.

10. McCarthy JS, Lotharius J, Ruckle T, et al. Safety, tolerability, pharmacokinetics, and activity of the novel long-acting antimalarial DSM265: a two-part first-in-human phase 1a/1b randomised study. *Lancet Infect Dis.* 2017;17:626-635.

11. McCarthy JS, Donini C, Chalon S, et al. A Phase 1, Placebo-controlled, Randomized, Single Ascending Dose Study and a Volunteer Infection Study to Characterize the Safety, Pharmacokinetics, and Antimalarial Activity of the Plasmodium Phosphatidylinositol 4-Kinase Inhibitor MMV390048. *Clin Infect Dis.* 2020;71:e657-e664.
