# Supplementary material for: Development and application of a PBPK modeling strategy to support antimalarial drug development
Source: CPT Pharmacometrics Syst Pharmacol. 2023 Aug 16;12(9):1335–46. doi: 10.1002/psp4.13013 (PMC10508484; doi:10.1002/psp4.13013)
Supplement: Supplementary file 5 — Appendix S2 [file PSP4-12-1335-s010.pdf]

Drug Specific Summary of Model Development and Verification

Key information pertaining to model development and verification for each compound is provided below. This text serves as a companion to Table S1 (model inputs), Table S2 (model verification - exposure) and Table S3 (model verification – DDIs) in which full references are provided.

Amodiaquine

The absorption of amodiaquine was described in the model using the first order model. Distribution of amodiaquine was described using the full PBPK model. A Kp scalar of 1.5 was used to recover the clinical observed data. Clearance was back calculated from clinical data. The fraction metabolized by CYP2C8 was defined based on trimethoprim DDI study. A measured renal clearance was incorporated. Inhibition of CYP2D6 was incorporated into the model.

Four clinical studies describing single and multiple dose exposure of amodiaquine were used to verify the PBPK model. In comparison of predicted *vs.* observed AUC, 75% of the studies were 2-fold and 50% were within 1.5-fold. A clinical DDI study where amodiaquine was the victim of a CYP2C8-mediated DDI was accurately recovered using the PBPK model. Therefore, the model performance was deemed acceptable and the application of the model as a CYP2C8 victim to assess DDI liability is verified. In vitro parameters conferring CYP2D6 inhibition potency are included in the model. In the absence of clinical data to verify the model of a perpetrator of CYP2D6-mediated DDIs, use of the model for this application should be accompanied by appropriate sensitivity analysis.

Artemether

The absorption of artemether was described in the model using the first order model assuming complete absorption in the fed state. Distribution of artemether was described using the full PBPK model. A Kp scalar (= 0.5) was used in the model along with optimized partitioning into adipose tissue ($K_{p,adipose}$ = 0.5) to recover the clinical observed data. Clearance was back calculated from clinical data. The fractions metabolised by CYP2B6 and CYP3A4 were defined based on in vitro data. Induction of CYP2B6 was incorporated into the model.

Two clinical studies describing single dose exposure and two describing multiple dose exposure of artemether were used to verify the PBPK model. The single dose exposures were within 1.5-fold of observed for both studies. The multiple dose exposures were slightly over-predicted at 2.02 and 2.63-fold for the two studies. Clinical DDI studies with ketoconazole, rifampicin and efavirenz where artemether was the victim of CYP3A4 (and CYP2B6 for efavirenz)-mediated DDIs were accurately recovered (within 1.25-fold) using the PBPK model. A clinical DDI study with efavirenz, where artemether was the perpetrator of a CYP2B6-mediated DDI was accurately recovered (within 1.25-fold) using the PBPK model. As the DDIs with all 3 inhibitors/inducers were well predicted, the fmCYP values were considered verified. The tendency towards over-prediction of artemether exposure upon multiple dosing could indicate a greater extent of induction is required. However, any increase in induction potency resulted in under-prediction of single dose exposure, which is of greater importance for the therapeutic effect of artemether. In addition, the CYP2B6 induction parameters used resulted in a good prediction of the impact of artemether on efavirenz PK. Overall, the artemether model was deemed acceptable and verified for its intended use as a CYP3A4 and CYP2B6 victim to assess the DDI liability.

Atovaquone

The absorption of atovaquone was described in the model using the first order model. Distribution of atovaquone was described using the full PBPK model. A Kp scalar was used in the model to recover

the clinical observed data. Clearance was back calculated from clinical data. A biliary CL_{int} was input based on clinical data.

Two clinical studies describing single and multiple dose exposure of atovaquone were used to verify the PBPK model. 100% of studies were within 1.5-fold. Thus, the model performance was deemed acceptable. There are some data to suggest atovaquone is an inhibitor of BCRP. This is currently not included within the model.

Azithromycin

The absorption of azithromycin was described in the model using the first order model. Distribution of azithromycin was described using the full PBPK model. A Kp scalar was used in the model to recover the clinical observed data. Clearance was extrapolated from in vitro data/back calculated from clinical data. The fraction eliminated by biliary and renal excretion was defined using clinical data. Inhibition of CYP3A4 was incorporated into the model.

3 clinical studies describing single and multiple dose exposure of azithromycin were used to verify the PBPK model, of which 100% were within 1.5-fold. Three clinical DDI studies where azithromycin was the perpetrator of aCYP3A4-mediated DDI were accurately recovered using the PBPK model. Hence the model performance was deemed acceptable.

Carboxyprimaquine

The formation of carboxyprimaquine was described assuming that all primaquine metabolized by MAO resulted in the formation of carboxyprimaquine. Distribution of carboxyprimaquine was described using the full PBPK model. Clearance was assigned as an IV clearance and was manually refined to describe the clinical data.

Six clinical studies describing single and multiple dose exposure of carboxyprimaquine were used to verify the PBPK model. The AUC for all verification studies were within 1.5-fold of the observed values. Thus, the model performance was deemed acceptable.

Chloroquine

The absorption of chloroquine was described in the model using the first order model assuming complete absorption. Distribution of chloroquine was described using the full PBPK model (Method 3) with subcellular distribution selected. Clearance was back calculated from clinical data following an intravenous dose. The fraction metabolised by CYP3A4 (12%) and CYP2C8 (22%) were defined based on in vitro data alongside renal clearance (57%). Inhibition was incorporated into the model based on in vitro inhibition.

A clinical study describing single dose exposure of chloroquine following 3 formulations (IV, oral tablet and oral solution) were used to verify the PBPK model. 100% of studies were within 1.5-fold. No clinical DDI data are available to verify the victim or perpetrator liability of chloroquine. One complexity in chloroquine model development was the wide range in literature B:P values (ranging from 3.3 to 10 derived between 1984 and 2020. Here a value generated by Charman et al., 2020 was selected (3.5, derived under pH-controlled conditions) but clearly use of different values would alter parameters such as the predicted distribution within the model. This was assessed during model development and the most recent parameter which was in line with other recent literature values was assumed.

Cycloguanil

The formation of cycloguanil was described assuming that all cycloguanil was formed by CYP2C19 and CYP3A4. Distribution of cycloguanil was described using the full PBPK model. The fraction of proguanil metabolised by CYP2C19 and CYP3A4 was defined based on clinical data. Clearance was assigned as an IV clearance and was manually refined to describe the clinical data.

5 clinical studies describing single and multiple dose exposure of cycloguanil were used to verify the PBPK model. 60% of studies were within 2-fold, of which 40% were within 1.5-fold. Prediction of cycloguanil exposure was complicated by the polymorphism classification of subjects in each study, hence the model performance was deemed acceptable.

DHA

DHA can be administered itself or can be formed following the administration of artemesinins such as artesunate or artemether. Two models were developed, DHA for use when simulating administration of DHA and when formed from artemether, and a separate model 'DHA from artesunate' for use when simulating artesunate administration. 'DHA from artesunate' was also developed by altering the absorption properties to mimic the rapid formation of DHA from artesunate. Further details of both models are described below:

DHA: - The absorption of DHA was described in the model using the first order model. Distribution of DHA from artesunate was described using the full PBPK model. A Kp scalar was used in the model to recover the clinical observed data. Clearance was back calculated from clinical data. The fraction metabolised by UGT1A9 and UGT2B7 was defined based on in vitro data. Inhibition of CYP1A2 was incorporated into the model.

Four clinical studies describing single dose exposure of DHA were used to verify the PBPK model. 100% of studies were within 2-fold, of which 75% were within 1.5-fold. Thus, the model performance was deemed acceptable. The model does not account for the differences in plasma fraction unbound observed in patients compared to healthy volunteers.

DHA from Artesunate:- The absorption of 'DHA from artesunate' was described in the model using the first order model. The absorption model does not consider the formation of 'DHA from artesunate' mechanistically. Instead, an optimized ka and fa were applied to the DHA model to describe the observed plasma concentration-time curve of DHA. The remainder of the DHA model was identical to the DHA model which is described above.

One clinical study describing single dose exposure of DHA was used to verify the PBPK model. 100% of studies were within 2-fold, of which 100% were within 1.5-fold. Thus, the model performance was deemed acceptable.

DEAQ

DEAQ was modeled as the metabolite formed from amodiaquine via the CYP2C8 metabolic pathway. Distribution of DEAQ was described using the full PBPK model. No scaling was used in V_{SS} prediction. A measured renal clearance was incorporated. Inhibition of CYP2D6 was incorporated into the model despite not affecting amodiaquine PK.

Simulations based on 1 clinical study describing multiple dose exposure of DHEA and 1 DDI study where formation of DHEA was the victim of a CYP2C9 mediated DDI were both within 1.5-fold of the observed values. Therefore, the model performance was deemed acceptable.

Doxycycline

The absorption of doxycycline was described in the model using a first order model assuming a high fraction absorbed of 0.95. Distribution of doxycycline was described using a full PBPK model, with a Method 2 predicted Vss of 0.653 L/kg. A Kp scalar of 0.3 was used in the distribution component of the model to recover observed clinical data. Clearance was back calculated from clinical IV data and divided into biliary, hepatic, and renal components.

Seven clinical studies describing single and multiple dose exposure of doxycycline were used to verify the PBPK model. The model predicted AUC values in 86% of studies within 2-fold (100% if one simulated/observed ratio is rounded down from 1.52 to 1.5), of which 57% were within 1.5-fold. The model also predicted C_{max} values in 100% of studies within 2-fold, of which 80% were within 1.5-fold. Thus, the model performance was deemed acceptable for doses between 100 to 200 mg. It should be noted that dose non-linearity of doxycycline outside this range is not incorporated into the model.

Lumefantrine

The absorption of lumefantrine was described in the model using the first order model assuming complete absorption in the fed state. Distribution of lumefantrine was described using the full PBPK model. A Kp scalar (= 0.04) was used in the model along with optimized partitioning into adipose tissue ($K_{p,adipose}$ = 7) to recover the clinical observed data. Clearance was back calculated from clinical data. The fraction metabolised by CYP3A4 was defined based on the clinical DDI study with ketoconazole. Inhibition of CYP2D6 was incorporated into the model.

Two clinical studies describing single dose exposure and two describing multiple dose exposure of lumefantrine were used to verify the PBPK model. The single dose exposures were within 2-fold of observed for both studies. The multiple dose exposures were within 1.25-fold of observed for both studies. Clinical DDI studies with rifampicin and efavirenz in healthy volunteers where lumefantrine was the victim of CYP3A4-mediated DDIs were over-predicted (>2-fold) using the PBPK model, however, an alternative clinical efavirenz DDI study in HIV patients and a clinical DDI with ritonavir in healthy volunteers were well predicted (within 1.25-fold of observed). As the effect of CYP3A4 inhibition was independently verified and there appeared to be variability in the extent of induction on lumefantrine PK, the fmCYP3A4 of 40% was considered verified. In addition, the Day 7 plasma concentrations following repeat administration of lumefantrine, that were more accurately predicted than single dose data, are more critical (linked to cure rates) than Day 1 values and hence the model performance was deemed acceptable.

Mefloquine

The absorption of mefloquine was described in the model using the first order model. Distribution of mefloquine was described using the full PBPK model and the clearance was back calculated from clinical data. The fraction metabolized by CYP3A4 was defined based on in vitro data. Inhibition of CYP2C9, CYP2D6, and CYP3A4 was incorporated into the model.

Six clinical studies describing single and multiple dose exposure of mefloquine were used the verify the PBPK model. Most of the studies (83%) were within 1.5-fold, with all simulations falling within 2-fold of the observed values. Two clinical DDI studies where mefloquine was the victim of a CYP3A4-mediated DDI were accurately recovered using the PBPK model. The model performance was deemed acceptable based on the verification and performance as a victim of CYP3A4-mediated DDIs. Although in vitro inhibition potency for CYP2C9, CYP2D6 and CYP3A4 were incorporated into the model, clinical DDI data were not available to verify model performance and so applications of the model for this purpose should be accompanied by appropriate sensitivity analysis.

Piperaquine

The absorption of piperaquine was described in the model using the first order model. As dose and food-dependent changes in absorption have been noted, three files were created with the fraction absorbed (fa) for fed (fa = 1.0) or dose-dependent fasted (960 mg fa = 0.40, 1280 mg fa = 0.33) states. Distribution of piperaquine was described using the full PBPK model. A Kp scalar (= 3) was used in the model along with increased portioning into adipose ($K_{p,adipose}$ = 115) to recover the clinical observed data. Clearance was extrapolated from *in vitro* data. The fraction metabolized by CYP3A4, CYP2C9, and CYP2C19 was defined based on *in vitro* data. Inhibition of CYP3A4 was incorporated into the model.

As significant variability in the PK of piperaquine between formulations has been observed, the model development was focused on the available Eurartesim data in healthy Caucasian subjects. Two clinical studies with fasted and fed groups at varying dose levels describing single and multiple dose exposure of piperaquine were used to verify the PBPK model. All of the simulated studies were within 1.5-fold of the observed values. A clinical DDI study where piperaquine was the victim of a CYP3A4-mediated DDI was accurately recovered using the PBPK model as well as a CYP3A4 perpetrator DDI with the sensitive substrate midazolam. From these observations, the model performance was deemed acceptable.

Primaquine

The absorption of primaquine was described in the model using the first order model. Distribution of primaquine was described using the full PBPK model. A Kp scalar was used in the model to recover the clinical observed data. Clearance was back calculated from clinical data. The fraction metabolised by CYP2D6 and MAO was defined based on in vitro data. The 'user UGT' enzyme was used as a surrogate for MAO within the Simulator.

Six clinical studies describing single and multiple dose exposure of primaquine were used the verify the PBPK model. 100% of the studies were within 1.5-fold. Thus, the model performance was deemed acceptable. It should be noted that the active metabolite(s) for primaquine are not characterized due to their instability and so cannot be defined in their own right within the model. However, as CYP2D6 has been implicated in the success of primaquine therapy, simulations examining the effect of interactions on CYP2D6 activity may provide an indirect surrogate to some extent.

Proguanil

The absorption of proguanil was described in the model using the first order model. Distribution of proguanil was described using the full PBPK model. A Kp scalar was used in the model to recover the clinical observed data. Clearance was extrapolated from back calculated from clinical data. The fraction metabolised by CYP2C19 and CYP3A4 was defined based on clinical data. Inhibition of CYP2D6 was incorporated into the model.

3 clinical studies describing single and multiple dose exposure of proguanil were used to verify the PBPK model. 66% of studies were within 2-fold, of which 33% were within 1.5-fold. A clinical DDI study where proguanil was the victim of a CYP2C19-mediated DDI was accurately recovered using the PBPK model. Prediction of proguanil exposure was complicated by not knowing the polymorphism classification of subjects in each study, hence the model performance was deemed acceptable using the criteria of being within 2-fold of observed.

Pyrimethamine

The absorption of pyrimethamine was described in the model using the first order model. Distribution of pyrimethamine was described using the full PBPK model. A Kp scalar (= 0.5) was applied to recover the clinical observed data. Clearance was extrapolated from in vitro data and incorporated as a microsomal clearance as the enzymes involved in the metabolism of pyrimethamine have not yet been determined. Consequently, the pyrimethamine model cannot be used as a victim of DDIs or be used to simulate exposure in special populations where metabolic routes may be altered.

Three clinical studies were available for model verification. 100% of simulated Cmax and AUC were within 1.5-fold of observed and hence the model performance was deemed acceptable.

Pyronaridine

The absorption of pyronaridine was described in the model using the first order. Distribution of pyronaridine was described using the full PBPK model (Method 3) with a Kp scalar (= 0.7) and a Kp muscle (182) was applied to recover the clinical observed data. Clearance was back calculated from clinical data. The fraction metabolised by CYP1A2, CYP2B6, CYP2C8, CYP2D6 and CYP3A4 was defined based on in vitro data. Inhibition of CYP2D6 and P-gp was incorporated into the model.

Two clinical studies describing pyronaridine exposure were available for model verification. 100% of predicted Cmax were within 1.5-fold of those observed whereas 40% of AUC were predicted within 1.5-fold of observed. This can be explained as observed exposure at 9mg/kg dose was lower than at 6 mg/kg. The model recovered the observed data at the 6 mg/kg dose but then over predicted that at the higher dose. One challenge in the verification of the model is the diverse ethnicities of subjects in reported clinical data and how best to reflect this in simulations. In the absence of virtual Korean populations within the Simulator, the Caucasian population was modified in terms of bodyweight. In the absence of supporting information, no changes to enzyme abundance (pmol/mg) were made to the population, although changes to liver weight (as a function of body weight) and hence total CYP abundance were propagated into the model. With these complexities in mind and seeing the performance of the simulated concentration-time profiles, the model was deemed verified.

Quinine

The absorption of quinine was described in the model using the first order. Distribution of quinine was described using the minimal PBPK model and a Kp scalar (= 0.65) was applied to recover the clinical observed data. Clearance was back calculated from clinical data. The fraction metabolised by CYP3A4 was defined based on a clinical DDI study with troleandomycin. Inhibition of CYP2D6 was incorporated into the model.

Three clinical studies describing Quinine PK were identified for model verification. All studiesThree clinical DDI studies where quinine was the victim of CYP-mediated DDIs were used to verify the PBPK model. All studies were well recovered with simulated C_{max} and AUC GMRs within 1.5-fold of the observed. Therefore, the model performance was deemed acceptable.

Sulfadoxine

The absorption of sulfadoxine was described in the model using the first order model. Distribution of sulfadoxine was described using the minimal PBPK model. Clearance back calculated from clinical data, allocating 90% to renal clearance and 10% to a non-specific hepatic microsomal metabolism.

Four clinical studies describing single and multiple dose exposure of sulfadoxine were used to verify the PBPK model. In comparison of predicted *vs.* observed AUC, 100% of the studies were within 2-fold and 75% were within 1.5-fold. Thus, the model performance was deemed acceptable.

Four clinical studies describing single and multiple dose exposure of pyrimethamine were used to verify the PBPK model. All simulations were within 1.5-fold of the observed values; hence the model performance was deemed acceptable.

Tafenoquine

Tafenoquine is administered with food to increase its exposure and minimize gastrointestinal side effects. The PBPK model was therefore developed to recover the PK of tafenoquine in the fed state. The absorption of tafenoquine was described in the model using the first order model assuming complete absorption in the fed state. Distribution of tafenoquine was described using the full PBPK model (Method 3). A Kp scalar (1.5) was used in the model to recover the clinical observed data. Intrinsic clearance was back calculated from clinical data. Inhibition of CYP2C9 and CYP3A4 was included in the model. A complication with this model was that the range of fu values reported in the literature was extremely large, ranging from 0.0007 to 0.11 depending on the methodology used. After evaluation of the fu on the back calculated intrinsic clearance, a value of 0.005 was selected for modelling.

Four clinical studies describing single and multiple dose exposure of tafenoquine were used to verify the PBPK model, although some of these provided PK profiles and no PK parameters and *vice versa*. Of the clinical studies describing PK parameters, the model recovered 100% of the observed PK parameters within 1.5-fold (66% within 0.8-1.25-fold) and hence the model is considered predictive. It should be noted that in the absence of information defining the fm of drug metabolizing enzymes, an undefined liver intrinsic clearance is used in the model and hence the model is not able to simulate the liability of tafenoquine as a victim of DDIs.

DSM265

The absorption of DSM265 was described in the model using the first order model. The PK was dose proportional up to the 250 mg dose, suggesting a reduced absorption at higher doses. Therefore, a fraction absorbed of 0.7 was used for doses of 400 mg and above, while for lower doses a fraction absorbed of 1 is recommended. Distribution of DSM265 was described using the minimal PBPK model. Clearance was back calculated from clinical data, allocating ~70% to CYP2C8 and ~30% to CYP2C19 (based on in vitro data). DSM265 was assigned as a neutral compound, since a basic pKa could not be measured using potentiometric assay and 2 in silico packages did not find any basic centers.

The model was developed using clinical data in healthy volunteers (McCarthy et al. 2017), who received a single dose of 25 mg. It was verified using the data from the same study for healthy volunteers who received higher doses in the fasted state (400, 600, 800 and 1200 mg). The model recovered 100% of the observed PK parameters within 1.5-fold. Since the predicted efficacious dose was 400 mg, the model was deemed fit for purpose and was applied to predict the DDI liability for a single dose of 400 mg.

DSM265 inhibits CYP2C8 in vitro (Ki,u = 1.18 µM) and this information was incorporated into the model. However, simulations with amodiaquine (CYP2C8 substrate) did not show any impact on CYP2C8 (AUC and Cmax ratios = 1.01) even when sensitivity analysis was carried out on Ki suggesting that the risk as a perpetrator on CYP2C8 substrates is minimal.

DSM450

DSM450 is the major metabolite of DSM265. Distribution of DSM450 was described using the minimal PBPK model. Clearance was back calculated from clinical data. As no in vitro data were available to

define the metabolic pathways the risk of DSM450 as a victim cannot be assessed. However, DSM450 inhibits UGT1A1 in vitro (Ki,u = 0.9 µM) which was incorporated into the model which can be used as a perpetrator file.

The model development and validation were performed using clinical data in healthy volunteers from the same study and the same doses used for DSM265 (McCarthy et al. 2017). The simulated PK profiles at all doses were within 1.5-fold of those observed clinically.

MMV048

The absorption of MMV048 was described in the model using the first order model. Distribution of MMV048 was described using the minimal PBPK model. Clearance was back calculated from clinical data, allocating 90% to UGT1A1 and 10% to CYP3A4 (based on in vitro data). No risk has been identified as a perpetrator based on the available in vitro data (CYP inhibition and PXR activation assay).

The model development was performed using a 40 mg single dose administered as tartaric acid tablets to improve the fraction absorbed (Sinxadi et al. 2020). It was validated using a clinical study performed at 40, 80 and 120 mg single doses in tartaric acid tablets (McCarthy et al., 2020). All simulations vs observations were within 2-fold, with a majority being within 1.5-fold. The DDI simulations were performed with a single dose of 120 mg which is the clinically relevant dose.
