# Supplementary figures and images for: Development and application of a PBPK modeling strategy to support antimalarial drug development
Source: CPT Pharmacometrics Syst Pharmacol. 2023 Aug 16;12(9):1335–46. doi: 10.1002/psp4.13013 (PMC10508484; doi:10.1002/psp4.13013)

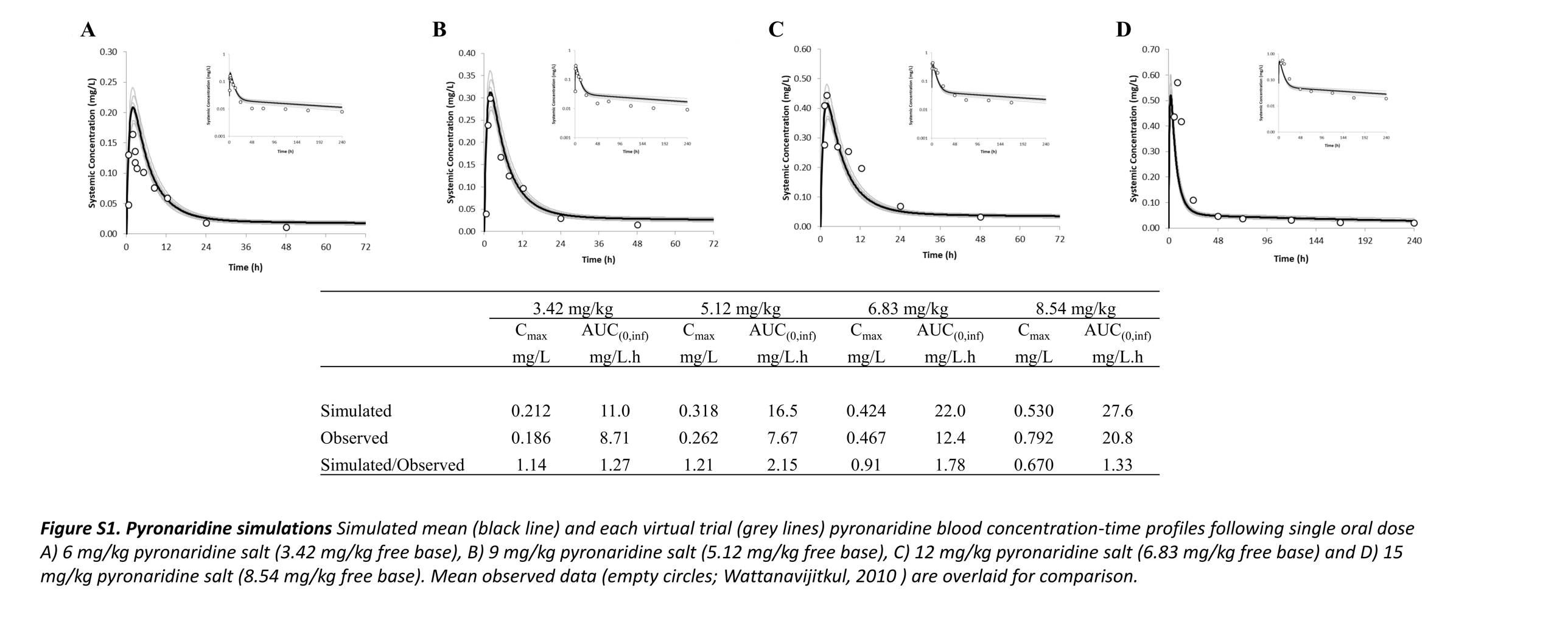

Supplement: Supplementary file 6 — Figure S1 [file PSP4-12-1335-s003.tif]

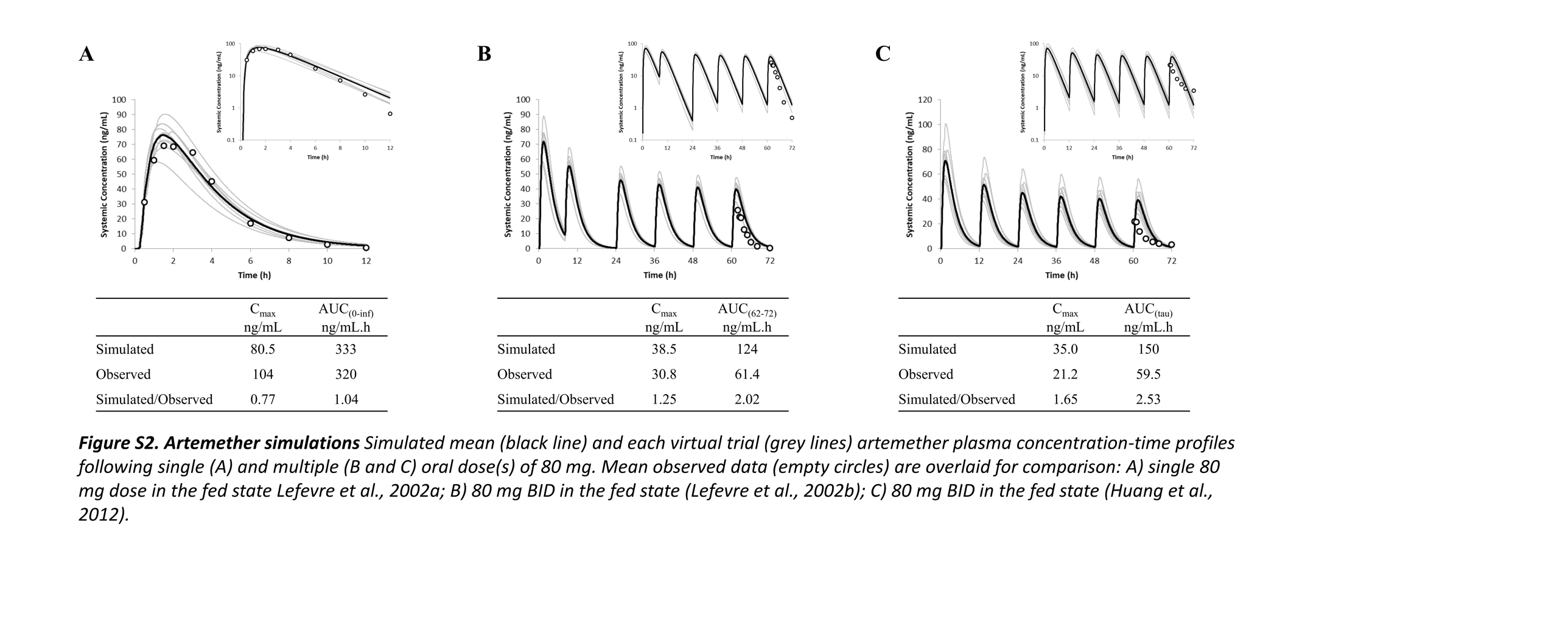

Supplement: Supplementary file 7 — Figure S2 [file PSP4-12-1335-s001.tif]

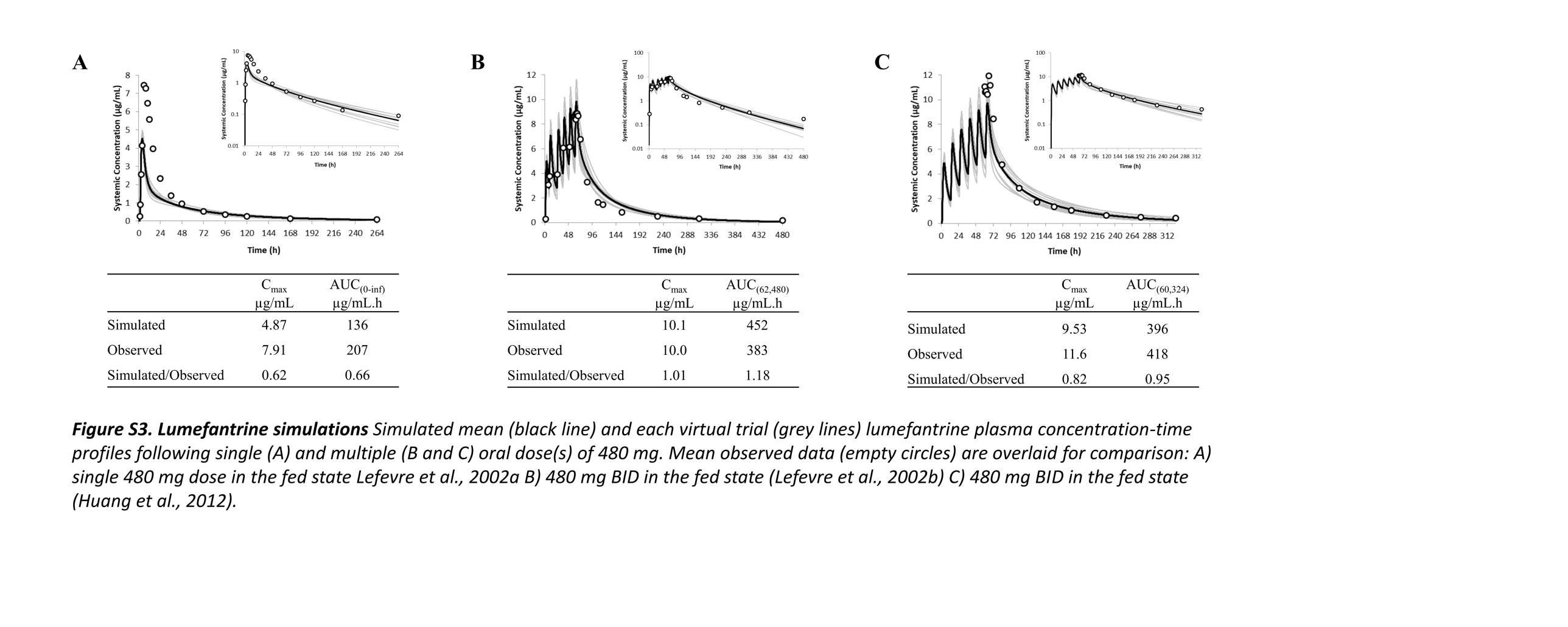

Supplement: Supplementary file 8 — Figure S3 [file PSP4-12-1335-s004.tif]

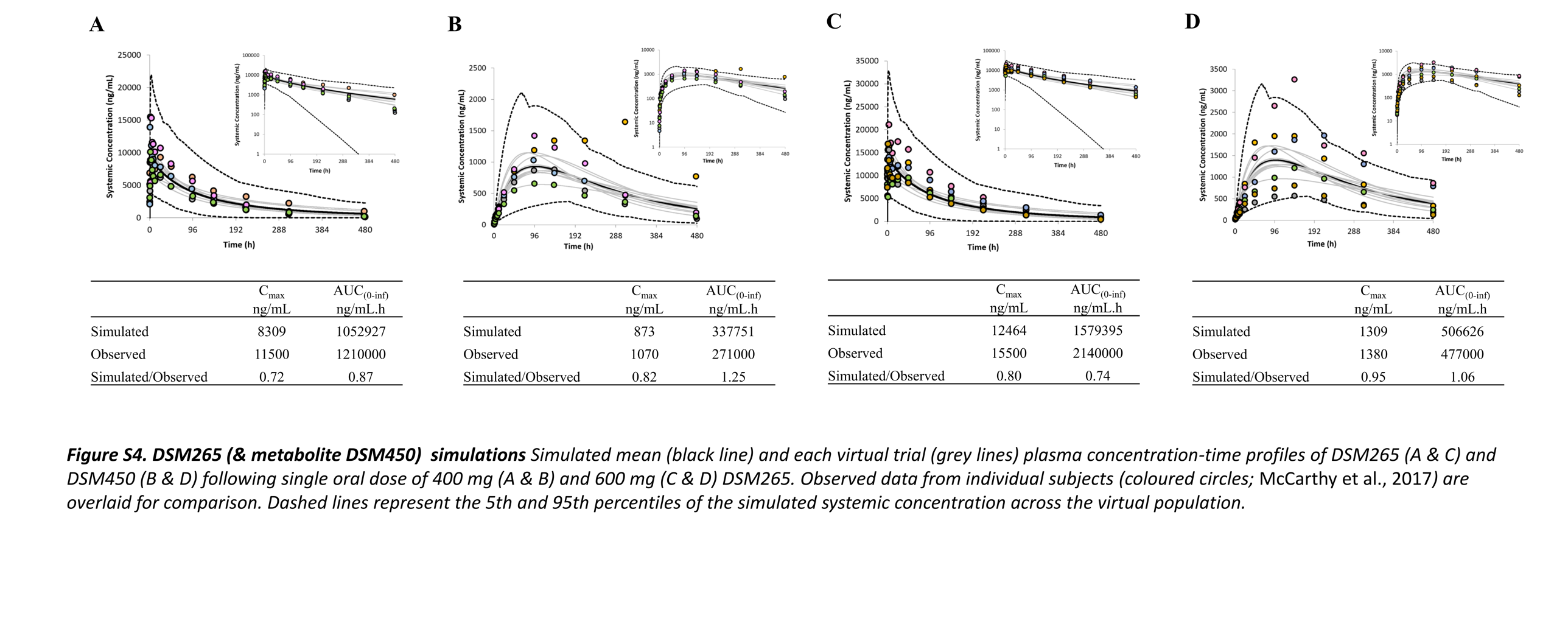

Supplement: Supplementary file 9 — Figure S4 [file PSP4-12-1335-s007.tif]

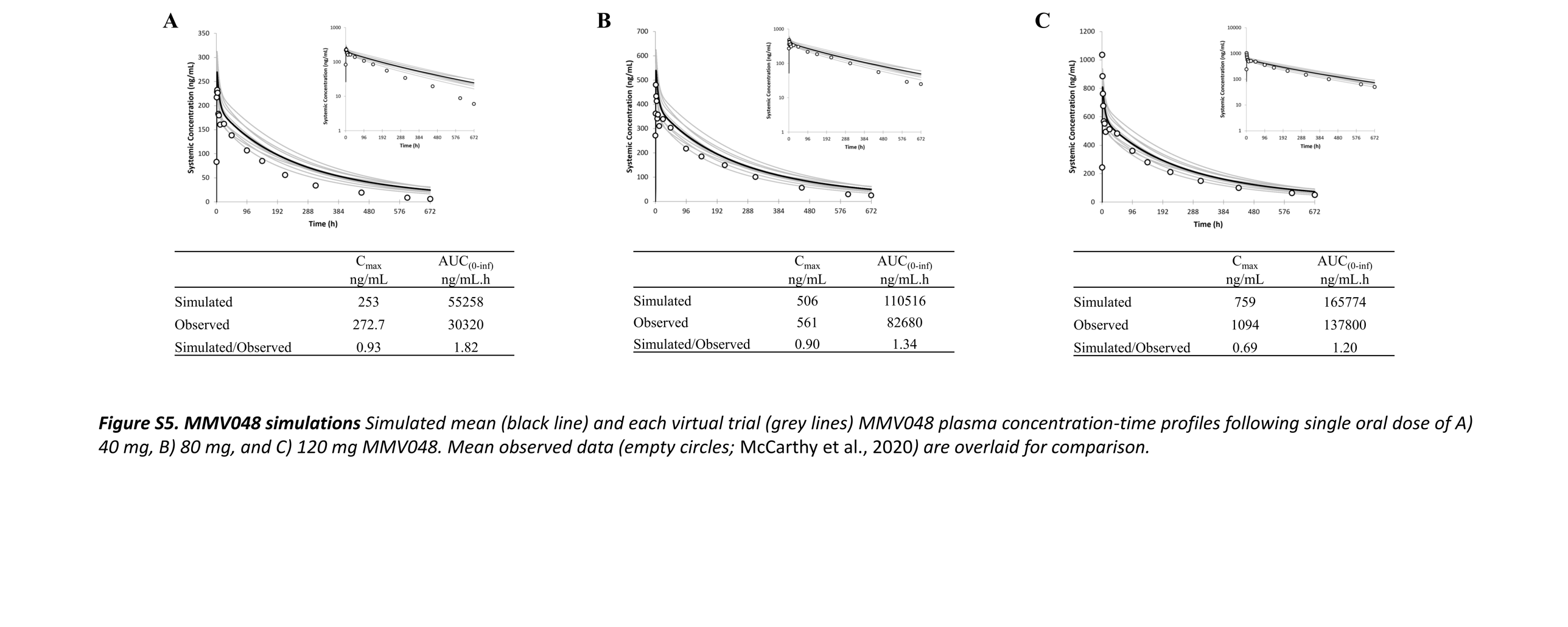

Supplement: Supplementary file 10 — Figure S5 [file PSP4-12-1335-s008.tif]
